# Network analyses based on comprehensive molecular interaction maps reveal robust control structures in yeast stress response pathways

**DOI:** 10.1038/npjsba.2015.18

**Published:** 2016-01-07

**Authors:** Eiryo Kawakami, Vivek K Singh, Kazuko Matsubara, Takashi Ishii, Yukiko Matsuoka, Takeshi Hase, Priya Kulkarni, Kenaz Siddiqui, Janhavi Kodilkar, Nitisha Danve, Indhupriya Subramanian, Manami Katoh, Yuki Shimizu-Yoshida, Samik Ghosh, Abhay Jere, Hiroaki Kitano

**Affiliations:** 1 Laboratory for Disease Systems Modeling, RIKEN-IMS, Kanagawa, Japan; 2 LABS, Persistent Systems Limited, Pune, India; 3 The Systems Biology Institute, Tokyo, Japan; 4 Sony Computer Science Laboratories, Inc., Tokyo, Japan; 5 Integrated Open Systems Unit, Okinawa Institute of Science and Technology, Okinawa, Japan

## Abstract

Cellular stress responses require exquisite coordination between intracellular signaling molecules to integrate multiple stimuli and actuate specific cellular behaviors. Deciphering the web of complex interactions underlying stress responses is a key challenge in understanding robust biological systems and has the potential to lead to the discovery of targeted therapeutics for diseases triggered by dysregulation of stress response pathways. We constructed large-scale molecular interaction maps of six major stress response pathways in *Saccharomyces cerevisiae* (baker’s or budding yeast). Biological findings from over 900 publications were converted into standardized graphical formats and integrated into a common framework. The maps are posted at http://www.yeast-maps.org/yeast-stress-response/ for browse and curation by the research community. On the basis of these maps, we undertook systematic analyses to unravel the underlying architecture of the networks. A series of network analyses revealed that yeast stress response pathways are organized in bow–tie structures, which have been proposed as universal sub-systems for robust biological regulation. Furthermore, we demonstrated a potential role for complexes in stabilizing the conserved core molecules of bow–tie structures. Specifically, complex-mediated reversible reactions, identified by network motif analyses, appeared to have an important role in buffering the concentration and activity of these core molecules. We propose complex-mediated reactions as a key mechanism mediating robust regulation of the yeast stress response. Thus, our comprehensive molecular interaction maps provide not only an integrated knowledge base, but also a platform for systematic network analyses to elucidate the underlying architecture in complex biological systems.

## Introduction

Living organisms are constantly affected by diverse internal and external stressors; for example, changes in nutrient and ion concentrations and temperature. They respond to such perturbations by orchestrating complex interactions between large numbers of intracellular molecules, including receptors, secondary messengers, modification enzymes, and transcription factors. This response to counteract stress stimuli is conserved throughout biology, from simple unicellular organisms to multicellular animals, and serves to maintain their homeostasis.

Stress response pathways are thought to allow organisms to transmit abrupt stimuli and trigger a range of cellular responses that enable the cell to respond properly to environmental challenges. Molecular mechanisms that can ensure stability of response include switch-like mechanisms, which generate threshold responses when stimuli reach a specific concentration.^1^ From the perspective of global network architectures, it has been argued that a bow–tie structure, where diverse stimuli sensing upstream signals converge into a limited number of ‘core’ molecules, which then trigger diverse effector molecules or genes, is an evolutionarily conserved core architecture of biological networks, a version of which can be observed in signaling networks.^2,3^

To understand the overall picture of molecular stress responses, we chose to investigate stress response pathways in *Saccharomyces cerevisiae* (baker’s or budding yeast). Budding yeast is a well-established model eukaryote organism, owing to its genetic and biochemical tractability, efficient growth ability, and the availability of extensive curated databases.^4,5^ It also shares stress response mechanisms, including MAPK cascades, heat shock chaperones, and redox proteins, with multicellular eukaryotes.^6–8^ Thus, insights obtained using budding yeast can be applied to multicellular organisms.

Dynamic modeling approaches are frequently used to understand the behaviors of signaling networks.^9–11^ However, such approaches are not scalable when networks contain more than a thousand states and interactions. Network analyses serve as powerful alternative tools to extract fundamental features from complex networks. Protein–protein interaction (PPI) and gene regulatory network^12–14^ are popular platforms for network analyses but often suffer from limited accuracy and a lack of detailed information. Therefore, we have taken a ‘deep curation’ approach to integrate experimentally derived network database data with information derived from publications.

Amassing a vast quantity of information from disparate sources into a common framework (namely, a large-scale molecular interaction map) can complement experimental efforts and provide a standardized format for subsequent network analyses. Current tools and standards in systems biology, such as CellDesigner, software designed to express various features of intracellular reactions in a graphical format,^15,16^ encoded using Systems Biology Markup Language (SBML; http://sbml.org/),^17^ provide computational platforms to collate and interpret such large-scale interaction maps. Using SBML and CellDesigner, signal transduction maps have been constructed for the epidermal growth factor receptor (EGFR), toll-like receptor (TLR), mammalian RB/E2F, and mammalian target of rapamycin (mTOR) signaling networks.^18–21^ In addition, consensus maps detailing the cell cycle processes and metabolic pathways of *S. cerevisiae* have been reported recently.^22,23^ However, to the best of our knowledge, no comprehensive molecular interaction map detailing a range of stress response pathways in *S. cerevisiae* currently exists.

In this paper, we first outline the creation of a map of yeast stress responses incorporating 26 different stimuli, grouped into six signaling pathways. On the basis of this map, we performed systematic, network-driven analyses to unravel three specific aspects of the underlying architecture of the network. First, bow–tie analyses demonstrated bow–tie structures for yeast stress responses, with a limited number of core molecules integrating multiple upstream signals and distributing these to downstream pathways. Second, controllability analyses indicated a characteristic control structure, with close correlations between bow–tie structures and experimental phenotypes. Finally, network motif analyses revealed characteristic motifs in stress response pathways, closely related to the core molecules within bow–tie structures.

Overall, we demonstrate a potent network-oriented strategy to elucidate underlying architectures in signaling networks, powered by comprehensive molecular maps.

## Results

### Comprehensive map characteristics

**Figure 1:**
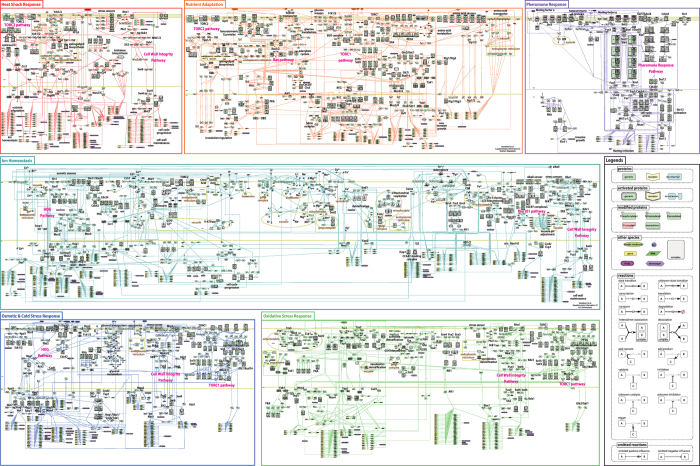
Comprehensive maps of the stress response pathways for the budding yeast, *Saccharomyces cerevisiae*, categorized into six groups: ion homeostasis; nutrient adaptation; osmotic and cold stress; oxidative stress; heat shock; and pheromone response. These maps were created with CellDesigner version 4.3.0. The SBML files and high-resolution image PDF files are available in Supplementary Information S2 and S3, respectively. The poster size version, integrating all six maps, is available in Supplementary Information S4.

In this study, we first constructed comprehensive yeast stress response maps (Figure 1) to capture biochemical reactions associated with the different biological events comprising stress responses in *S. cerevisiae*, including the binding of external ligands to specific receptors, signal transmission via kinase cascades, eventual delivery of signals to the cell nucleus, and gene transcriptional regulation.

Molecular species and their interactions were constructed using CellDesigner 4.3 editor by curating experimental evidence of functional interactions derived from published data (902 in total; Supplementary Information S1). The relevant publications relating to each interaction were stored in model notes and ‘MIRIAM (Minimum Information Required In the Annotation of Models),^24^ with links to the source database.

Using standardized notations from the Systems Biology Graphical Notation (SBGN) process description diagram,^25^ a mechanism-oriented view was included in the maps, capturing details such as state transition (phosphorylation, degradation, and transportation), complex formation, intracellular localization, and other biological features obtained from the literature. This detailed format not only enables informative summarization of data distributed across a vast literature, but also facilitates network analyses and mathematical model construction.

The source CellDesigner xml and PDF format files of the individual maps are provided in Supplementary Information S2.1, respectively. The poster size version of all six maps is also provided in Supplementary Information S4.

Twenty-six different signals (stimuli) are represented in the maps, including heat shock, dimethyl sulfoxide, cold shock, hyperosmolarity, hypo-osmolarity, zymolyase, alkali, H^+^, Na^+^, K^+^, Li^+^, Ca^2+^, Mg^2+^, Mn^2+^, Cu^2+^, Cd^2+^, Zn^2+^, Fe^3+^, oxidative stress, low glucose, amino acids, nitrogen source, glucose, citric acid, sorbic acid, and pheromone. According to standard biological classification,^26^ the pathways were categorized into the following six groups: (1) ion homeostasis; (2) nutrient adaptation; (3) osmotic stress; (4) oxidative stress; (5) heat shock; and (6) pheromone response. The biological features of each group are described in Supplementary Information (S5). The numbers of species and reactions in each of the six maps are provided in Table 1. The occurrence of specific entities in each of the six maps is summarized in Supplementary Information (S6).

### Bow–tie analyses

While there are reports of bow–tie structures in mammalian TLR and EGFR pathways,^18,19^ it is unclear whether such structures also exist in the stress response pathways of budding yeast. To evaluate whether yeast stress response pathways also contain bow–tie structures, we calculated bow–tie scores, (b(*m*)∈[0, 1]). The bow–tie score, b(*m*), represents the fraction of connecting paths between a source (*S*) and target (*T*) containing node (*m*).^27^ This score can be considered a specialized form of ‘betweenness centrality’, in which all possible connections from all vertices to all others are considered.^28^ Thus, nodes with high bow–tie scores are repeatedly used in various signaling pathways connecting sources and targets. We defined external stimuli (e.g., heat shock, ions, and osmotic stress) as sources and mRNAs expressed as a consequence of stress responses as targets.

In all six yeast stress response maps, we found that there were small numbers of nodes with high bow–tie scores, whereas the majority of other nodes had very low scores (Table 2), suggesting that bow–tie structures are indeed present in yeast stress response pathways. For instance, only four proteins, Hog1, Sho1, Pbs2, and Msn2, in the osmotic and cold stress response map had bow–tie scores >0.5. All of these molecules are core components of the high-osmolarlity glycerol (HOG) pathway, indicating that the majority of osmotic and cold response signals pass through the HOG pathway, as reported previously.^29^ Even when a threshold bow–tie score of >0.2 was considered high, only 20 molecular species (6.0%) were included in this category. By contrast, relatively large numbers of molecules with high bow–tie scores were identified in the pheromone response map. This suggests the presence of non-redundant core processes, composed of relatively large numbers of molecules, in the yeast pheromone response. In fact, in the pheromone response, mating signals are transmitted directly via multiple MAPK-related molecules, which are sequentially activated using Ste5 as a binding platform.^30^ Thus, bow–tie scores clearly provide important information about the architectures of signaling pathways in yeast stress responses. We defined molecules with bow–tie scores >0.2 as candidate bow–tie cores (Supplementary information S7). In all six maps, molecules with high bow–tie scores connected densely with each other, suggesting that these core molecules co-operate in central processes. Overall, bow–tie analyses revealed important bottlenecks in each stress response pathway.

As expected bow–tie scores demonstrated a degree of correlation with those for betweenness centrality in each map (Table 2; *R* ^2^=0.22–0.89), likely due to generic similarities between the two measures in capturing network structure. However, there are a number of connections that may be unrelated to signaling flow, particularly in signaling pathways rich in branched and reversible reactions, such as the ion homeostasis and the heat shock responses. In these instances, betweenness centrality does not clearly represent the importance of particular nodes in biological signaling pathways. Moreover, nodes with high bow–tie scores do not necessarily have a high degree, indicating that bow–tie cores are different from network hubs. Thus, the bow–tie score is suitable for investigating network structure and identifying core molecules, particularly in networks with directional signaling. The bow–tie and betweenness centrality scores, and degree, are summarized in Supplementary information S8.1 for all molecules. When we applied our analysis method to previously published signaling maps, we found that molecules that have been proposed as bow–tie cores in the TLR and EGFR pathways^18,19^ also had high bow–tie scores (in TLR pathway, NF-κB/CBP: b(*m*)=0.89 and MyD88: b(*m*)=0.45; and in EGFR pathway, Rac/Cdc42: b(*m*)=0.70 and PI3,4,5-P_3_: b(*m*)=0.63), supporting the validity of the bow–tie score as a method for identifying core molecules.

**Figure 2:**
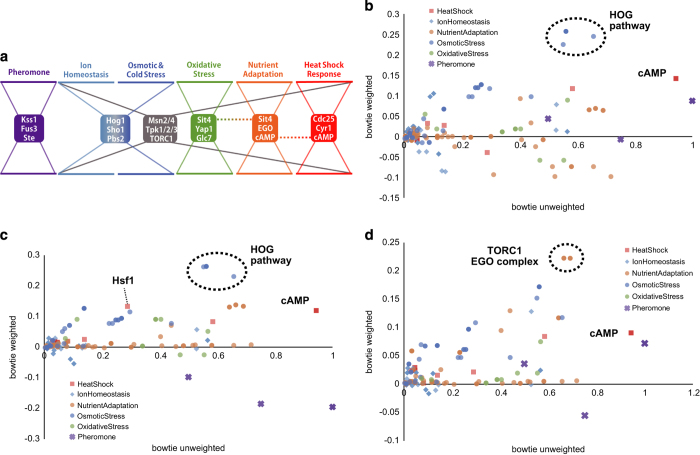
(**a**) An illustrative representation of bow–tie structures identified in yeast stress response pathways. In addition to core molecules in individual stress response pathways, some molecules, including Msn2/4, Tpk1/2/3, and TORC1, appeared repeatedly in multiple stress response pathways, indicating a global bow–tie structure throughout the yeast stress response (gray dotted lines). Plots of unweighted bow–tie scores against bow–tie scores using transcriptome data under conditions of (**b**) NaCl treatment, (**c**) heat shock treatment, and (**d**) a rapamycin treatment. Bow–tie core molecules with characteristically high-weighted bow–tie scores under each stress condition were indicated in the graph (e.g., HOG pathway and cAMP).

In addition to core molecules in the individual maps, we also found that a few molecules, including Msn2/4, Tpk1/2/3, and TORC1, had high bow–tie scores in the majority of maps analyzed. For example, Msn2 exhibited bow–tie scores of >0.20 in all stress response maps, with the exception of the pheromone response map. These molecules receive various different stress signals and broadly control stress-responding genes, indicating a global bow–tie structure throughout the yeast stress response (Figure 2a).

Next, we evaluated the maps we constructed as tools for estimation of the sites of action of drugs or stressors. To this end, each connecting path was weighted with differential expression change (log-fold change) of target mRNA. Weighting in this way allowed us to evaluate whether each bow–tie core molecule is also important as a signaling bottleneck under specific conditions. After NaCl treatment,^31^ components of the HOG pathway (Hog1, Pbs2, and Sho1) exhibited high ‘weighted’ bow–tie scores, indicating increased expression of genes downstream of the HOG pathway (Figure 2b). In addition, heat shock condition (measured at 45 min after a shift in culture conditions from 30 to 37 °C)^32^ induced a characteristic increase in the weighted bow–tie score of Hsf1 in the heat shock response map, whereas scores were ablated in core molecules related to the pheromone response (Figure 2c). Finally, we confirmed that TORC1 and EGO complexes displayed characteristic high bow–tie scores when stimulated with rapamycin^33^ (Figure 2d). This is consistent with the role of rapamycin in preventing activation of TORC1, which suppresses catabolic-related genes in nutrient-rich conditions. These results are consistent with previous studies indicating the efficacy of weighted bow–tie analyses for integrating transcriptome data to estimate important signaling bottlenecks under specific conditions. Our analyses indicate that our methods will be useful for elucidating the functional mechanisms of uncharacterized drugs.

### Controllability analyses

**Figure 3:**
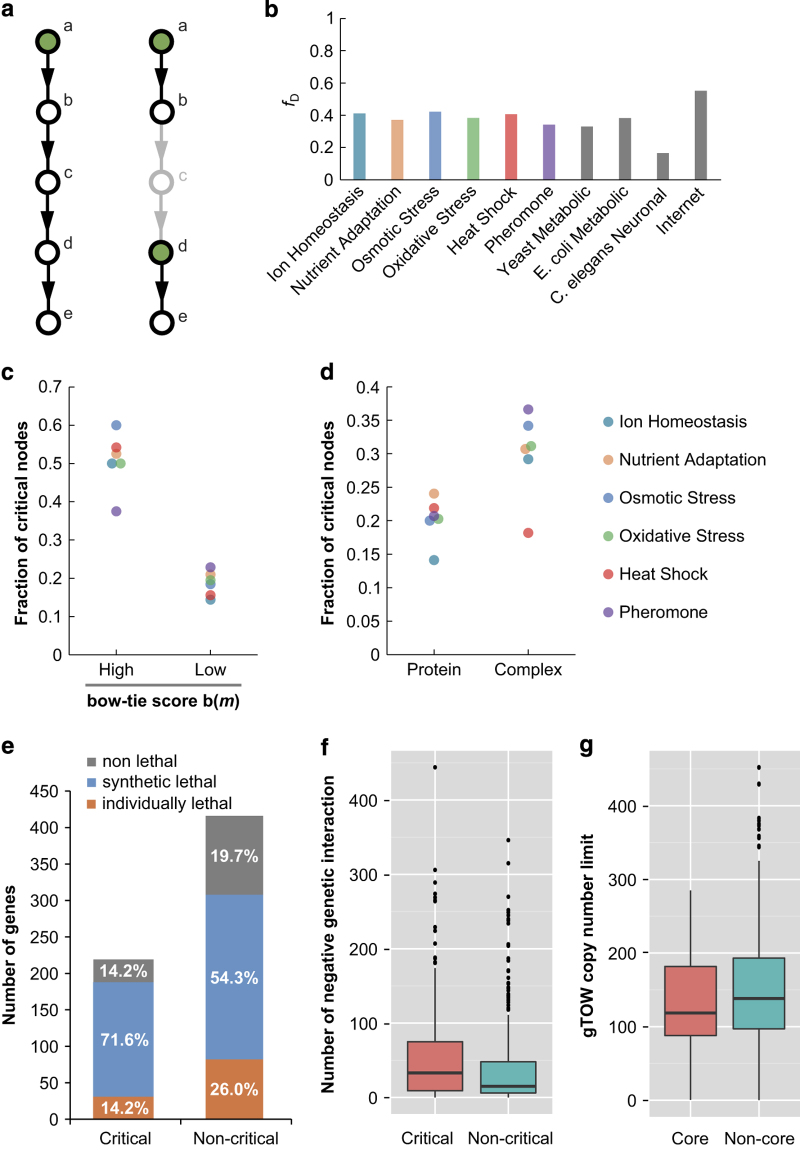
Controllability of the yeast stress response pathways. (**a**) Left is a simple example of a directed network model, in which driver node *a*, indicated in green, can control all other nodes. When node *c* is removed as depicted on the right, the regulatory path connecting *a* and its target nodes is disconnected. Consequently, nodes *d* and *e* need to be individually controlled and the number of driver nodes increase. Thus, node *c* is important in connecting a regulator and its target, and is defined as a critical node. (**b**) The fractions of driver nodes, *f*_D_, for the six yeast stress response maps and other reference networks. The data for the other reference networks were obtained from a controllability paper by Liu *et al*.^34^ (**c**) The fraction of critical nodes among those with high and low bow–tie scores, b(*m*), calculated for six yeast stress response maps. (**d**) The fraction of critical nodes among monomeric proteins and complexes calculated in six yeast stress response maps. (**e**) Number of proteins whose corresponding genes are individually lethal, with at least one synthetic lethal interaction, and non-lethal phenotypes, with respect to critical and non-critical nodes. The phenotype relating to each gene was obtained from the SGD database. (**f**) The distribution of negative genetic interactions of critical and non-critical nodes represented in a box-and-whisker plot. The bottom and top of the box represent the first and third quartiles (hinges), respectively. The line through the box shows the median. The whiskers extend from the hinges to the highest or lowest value within the 1.5 interquartile range. Data not included between the whiskers are plotted as dots. Negative genetic interactions were obtained from the SGD database. (**g**) The distribution of copy-number limit of overexpression measured using the genetic tug-of-war (gTOW) method^44^ with respect to bow–tie core-associated genes and non-bow–tie core genes represented in a box-and-whisker plot (as described for **f**).

Our bow–tie analyses indicated that the bow–tie structure may function as a central subsystem controlling the yeast stress response. To further investigate this phenomenon, we conducted controllability analyses to determine how individual molecules are controlled within bow–tie structures. Controllability analysis determines nodes required to control the network (driver nodes) by maximum matching in the network.^34^ A driver node can be considered as a regulator that controls downstream molecules via a directed path. If a large fraction of driver nodes (*f*_D_) are necessary for control, as in the case of the Internet,^35^ then the network does not have systematic regulation and each component node will be controlled separately. On the other hand, networks with a low *f*_D_, such as the neural network of *C. elegans*,^36^ are systematically controlled by a few master regulators. In addition to the driver nodes, we can also assess the importance of a node in regulatory signal transmission by removing it from a network and examining the effect on *f*_D_. If *f*_D_ increases when a node is removed, then the node is defined as a critical node, as the systematic network control is somewhat lost under conditions of node failure. In other words, losing a critical node means that some of the regulatory paths connecting regulators and their targets are disconnected, necessitating an increase in individual regulators (Figure 3a). Thus, critical nodes can be considered as important transmitters of regulatory signals. Biologically, this implies that inhibition of a molecule corresponding to a critical node by genetic knockout or treatment with an inhibitor would lead to dysregulation of several molecules controlled by using the critical node as a signal transmitter. Using controllability analysis, we investigated how systematically the yeast stress response is controlled, and explored the relationship between controllability and bow–tie structures.

As shown in Figure 3b, all of the stress response pathways examined exhibited moderate *f*_D_ values (~0.4) comparable to those of the metabolic networks of yeast and *E. coli*,^37^ suggesting that a moderate *f*_D_ is likely to be a common feature of self-assembled molecular biological networks, in which modular control is a widely observed mechanism effective for the local containment of perturbations and damage.^3^ Of interest, the majority of the bow–tie cores were not identified as driver nodes (Supplementary information S7 and S8). A substantial number of the driver nodes resided upstream of the bow–tie cores (Supplementary information S7). By contrast, we found that the fraction of critical nodes was significantly higher among those with high bow–tie scores (>0.20) compared with other nodes (*P*<0.001; paired *t*-test; Figure 3c). This indicates a potential role for bow–tie cores, not as regulators, but as important transmitters of regulatory signals. In addition, we found a significantly higher fraction of critical nodes among complexes than monomeric proteins (*P*=0.023, paired *t*-test; Figure 3d).

Next, we assessed whether controllability is correlated with experimental phenotypes created by genetic deletions. Systematic deletion analyses in the budding yeast have revealed that only a small fraction (~20%) of genes are individually indispensable, whereas combinations of mutations in two or more genes frequently lead to cell death, a phenomenon termed ‘synthetic lethality’. For comparison, genes whose corresponding proteins are included in at least one critical node (protein or complex) were designated ‘critical genes’. Although we did not find differences in the ratios of individually lethal genes between critical and non-critical gene categories, the ratio of genes with at least one synthetic lethal interaction was significantly higher for critical genes (*P*=8.3×10^−5^; *χ*^2^-test; Figure 3e). This indicates that critical proteins tend to increase the instability of other factors when they are deleted. We also evaluated whether controllability correlated with single-gene deletion phenotypes under specific stress conditions. In the ion homeostasis map, critical genes tended to have deletion phenotypes related to impaired stress responses to the ions (Supplementary Information S9). However, in the remaining maps, we did not observe similar phenomena. Instead, in the map relating to the oxidative stress response, a larger proportion of non-critical genes exhibited stress response defects. In summary, our results indicate that controllability does not correlate with single-gene deletion phenotypes under general or specific stress conditions. Recently, genome-wide synthetic genetic interactions have been quantitatively explored with high-throughput screens, including synthetic genetic arrays (SGAs).^38–41^ We next considered ‘negative genetic interactions’, which are defined as cases where synthetic genes combine to cause lethality or a negative effect on fitness, obtained using data derived from SGA screens. Genes determined to be critical in our controllability analyses had significantly more negative genetic interactions than non-critical genes (*P*=7.1×10^−5^; Mann–Whitney two-tailed *U-*test; Figure 3f), consistent with a correlation between these genes and synthetic lethality. These results suggest that the controllability of the molecular interaction maps reflects certain aspects of biological vulnerability to genetic deletion. The controllability and experimental phenotypes of proteins are summarized in Supplementary information S9.

As we have demonstrated the role of bow–tie cores in the signaling processes of yeast stress response pathways, it is interesting to compare these features with the bow–tie structures observed in metabolic networks. In metabolic processes, the bow–tie cores are tightly connected and exhibit robust small-world properties.^42^ This means that relatively small fluctuations in the molecules of bow–tie cores in metabolic processes, such as ATP and pyruvate, are lethal, and they are tightly regulated by allosteric and feedback mechanisms to inhibit fluctuations.^43^ By contrast, we found that the cores of signaling bow–tie structures do not appear to be as robustly regulated as those of metabolic networks. However, interestingly, most of the core molecules in yeast stress responses are robust against overexpression as measured using the genetic tug-of-war method.^44^ Of 76 bow–tie core-associated genes with bow–tie scores>0.20 in at least one yeast stress response map, only seven (BMH2, STE12, HSF1, TIP41, and TPK1/2/3; 9.2% of the total) are dosage sensitive, with a copy-number limit of 10 or less (Supplementary Information S9). This is higher than the dosage-sensitive gene ratio of non-bow–tie core genes (23 of 564 genes=4.1%; *P*=0.0739; Fisher’s exact test), but still limited. Also, we did not find a significant difference in the average copy-number limit between bow–tie core-associated genes and non-core genes (Figure 3g; *P*=0.156; Welch’s *t*-test). Therefore, we speculate that there may be some stabilization mechanisms to ensure robust signaling through bow–tie structures. One possible such mechanism is multistep activation of core molecules. For instance, to be functional, Msn2/4 must first be transported into the nucleus and then phosphorylated. It is thought that multistep activation mechanisms provide robustness to biological processes by the presence of several individual activation steps that insulate them from noise in the system. In addition, we consider that protein complexes are more likely to be critical nodes than monomers. Since the majority of the bow–tie cores observed in our study contained complexes, we constructed a working hypothesis that complexes have a specific role in the robust control of signaling networks.

Some complexes, for example, TORC1 (comprising of Tor1, Kog1, Lst8, and Tco89) and SBF (comprising of Swi4 and Swi6), become functional only when all components are assembled in a fixed ratio. This can prevent accidental activation of the complex because its overall function would be scarcely affected if one of the components was unexpectedly overexpressed or activated.^45^ Although the fixed stoichiometry of functional complexes is a potent mechanism for robust control, there are also a number of cores that function as monomeric proteins. Furthermore, it has been reported that dosage imbalance between complex components causes fragility,^46^ suggesting another mechanism of complex-mediated robust regulation.

### Network motif analyses

To elucidate the robust regulation of bow–tie cores in the yeast stress response, possibly mediated by protein complexes, network motif analyses were performed to identify local characteristic network structures. When biological networks are represented as graphs, a series of distinct substructures, namely, ‘network motifs’, appear in the graphs significantly more often than in randomized networks. Network motifs are thought to serve as the building blocks of the network, and the dynamic features and associated functions of some network motifs have been well investigated both theoretically and experimentally.^47–49^ Thus, network motifs closely relate to biological functions and, therefore, provide important clues to determine regulatory architectures.

**Figure 4:**
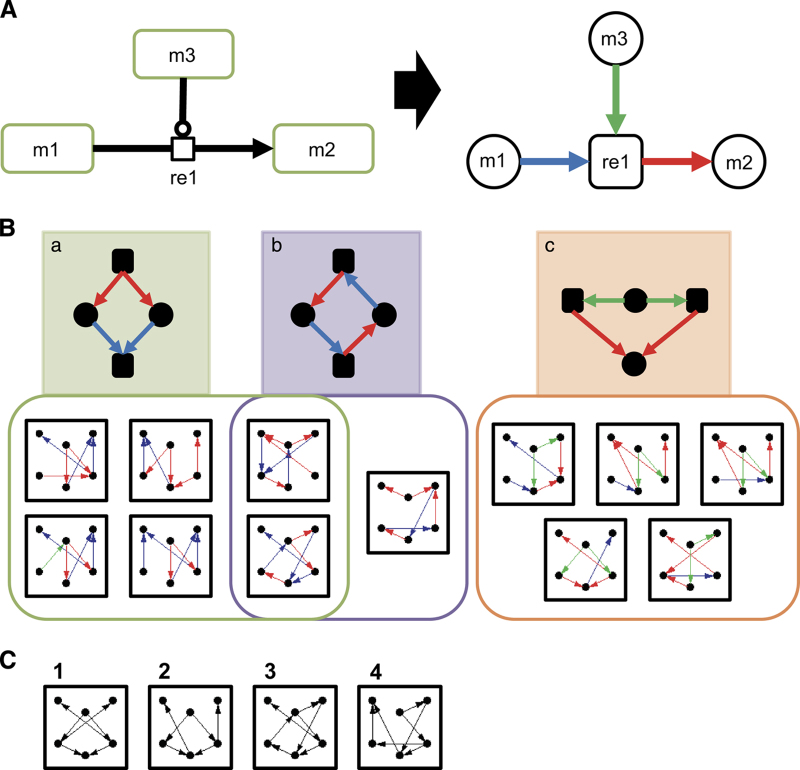
(**a**) Conversion of the maps to bipartite-directed graphs for network motif analyses. The map on the left is a representation of a typical reaction, in which a molecule (m1) transitions to another state (m2) through a reaction (re1) catalyzed by m3. We treated both molecules and reactions as nodes connected by directed edges. The types of molecules (e.g., proteins, complexes etc.) and reactions (e.g., modification, transport, and so on) were ignored. All edges from reaction nodes to molecule nodes are in red, indicating ‘product’ edge. Blue arrows indicate ‘reactant’ edge, from a reactant molecule node to a reaction node, whereas green arrows represent the ‘modifier’ edge, from an enzymatic molecule node, which acts as a modifier of the reaction, to reaction node. The types of modifier, such as positive catalysis and inhibition, were ignored. (**b**) Network motifs specific to stimuli response pathways. Among 30 motifs common to the six yeast stress response pathways, 12 also appeared in other stimuli response pathways (EGFR, TLR, and mTOR signaling pathway) but not in non-stimuli response pathways (yeast cell cycle and influenza replication). These 12 motifs can be categorized into three groups, based on substructures. Motif groups responsible for substructure (**a**, **b**, **c**) are represented in green, purple, and orange, respectively. (**c**) Network motifs specific to stimuli response pathways identified without regard to edge labels. Among 47 motifs common to six yeast stress response pathways, only four also appeared in other stimuli response pathways. All of these motifs contained some of the substructures described in **b**. For instance, monocolor motif 1 corresponds to substructures (**a**, **c**), whereas these are indistinguishable in the monocolor motif.

Using the comprehensive maps constructed in this study, we searched for network motifs specific to yeast stress response pathways. As depicted in Figure 4A, the maps were converted into bipartite-directed graphs, treating both molecules, and reactions as nodes. To distinguish the types of reactions, edges were labeled with three colors: blue arrows represent the ‘reactant’ edge, from reactant molecule to reaction nodes; red arrows represent the ‘product’ edge, from reaction to product molecule nodes; and green arrows represent the ‘catalysis’ edge, from enzymatic molecule to reaction nodes. Using this labeling method enabled us to make best use of the information contained in the detailed molecular interaction maps. In addition, to avoid a disproportionate emphasis on the trivial motifs, transcription, and translation, which occur most frequently in all biological networks, these were removed from the analyses. Consequently, 30 six-node motifs common in the yeast stress response maps were identified, which occurred with significant frequencies relative to 100 random networks generated by switching edges between nodes regarding the edge colors (*P*<0.05, *Z*-score>2, and occurred at least five times; Supplementary Information S10). To confirm specificity, we checked whether these motifs also appear in other established pathway maps. Interestingly, the majority of yeast stress response motifs (19–21 of 30 motifs) also appeared frequently in other pathways responding to external stimuli, including EGFR, TLR, and mTOR signaling pathways. By contrast, the motifs were less common in other networks, such as the yeast cell cycle and influenza replication pathways (7–14 of 30 motifs). These results suggest that some motifs are conserved among species and are characteristic of pathways responding to external stimuli.

Among the six-node motifs identified in the stimuli response pathways, 12 patterns were found, revealing three shared substructures (Figure 4B). The first substructure represented a ‘dissociation and recombination’ process (a), which constituted two different types of reaction, complex dissociation, and recombination (e.g., Tpk1/2/3-Bcy1, TORC1-Tap42, Gpa2-Gpb1-Gpg1, V-ATPase, and Msn2/4-Bmh2), and ionization equilibrium (e.g., MgHPO_4_⇔Mg^2+^+H^+^+PO_4_^3−^). The second substructure, ‘reversible reaction’ (b), is not a standalone motif, as it mostly occurred with substructure (a). Thus, these two substructures may have an important role in ‘reversible complex formation’ as part of the stress response pathways. The last substructure, ‘redundant reactions catalyzed by same molecule’ (c), was clearly a distinct category from the other two. Examples of this substructure include Crz1 dephosphorylation at distinct sites (all catalyzed by calcineurin); Bcy1 dissociation from Bcy1-Tpk1/2/3 complexes triggered by cAMP; and glutathione-dependent redox reaction catalyzed by Grx2. Instances of these motifs are provided in Supplementary Information S11.

To investigate the effect of edge labeling on motif analysis, we computed stimuli response pathway motifs disregarding the colored labels (i.e., following standard methods for network motif analysis). As shown in Figure 4C, four monocolor motifs were found using this analysis. Although all of these monocolor motifs appeared to contain the substructures identified by edge labeling, substructures (a) and (c) were not distinguished by this analysis. Thus, our method of colored motif analysis is capable of distinguishing biologically relevant substructures, which are not necessarily captured in standard monocolor motif analysis.

**Figure 5:**
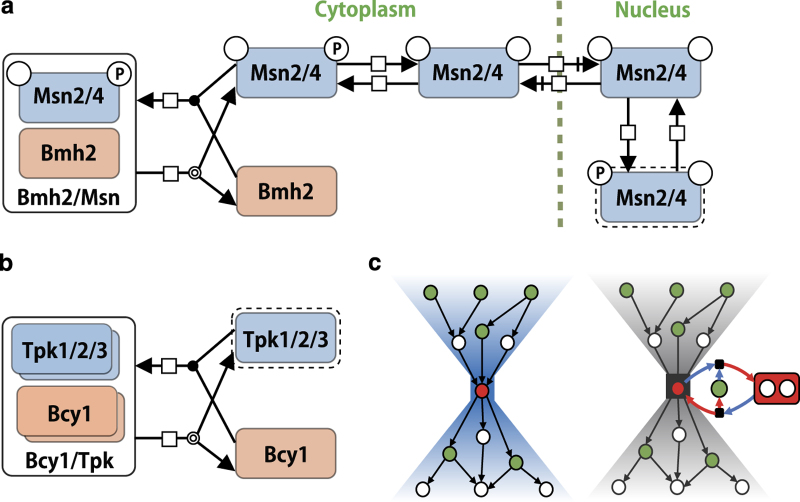
Diagrams representing regulation of (**a**) Msn2/4 and (**b**) Tpk1/2/3 by binding with their inhibitors, Bmh2 and Bcy1, respectively. Molecular interactions are described with graphical notations, complying with SBML and SBGN standards. Objects with dotted lines indicate ‘activated molecules’. (**c**) Simple congregative representation of the bow–tie structure, controllability, and complex-mediated network motifs. In the bow–tie structure, a limited number of core molecules integrate many input signals and regulate many downstream molecules. The bow–tie core connects many regulators (drivers represented in green) with their targets; thus it tends to be a critical node (red). We observed that the ‘reversible complex formation motif’ was often coupled with the bow–tie core (right). In such cases, the inhibitory complex is also a critical node, indicating the importance of the complex in control of the network.

In summary, network motif analyses applied to the detailed molecular interaction maps revealed characteristic motifs in stress response pathways. These rather simple motifs appeared repeatedly in the stress response pathways, with alterations in their components, suggesting that they represent ubiquitous principles of biological function. Interestingly, many of the network motifs related to core molecules of bow–tie structures. Whereas the ‘redundant reactions catalyzed by same molecule’ motif (Figures 4B, c) was clearly responsible for spreading the signal from the core, the ‘reversible complex formation’ motif (Figures 4B, a,b) appeared not to have roles in either signal integration or signal diffusion. Instead, complexes involved in this motif characteristically had roles in inactivating components. For instance, Bmh2 retains phosphorylated Msn2/4 in the cytoplasm, rendering it inactive (Figure 5a), whereas Bcy1 inhibits PKA activity by forming an inactive heterotetrameric complex with Tpk1/2/3 in the absence of cAMP (Figure 5b). According to the equilibrium principle, when the concentration of a core molecule is unexpectedly decreased, inhibitory complexes should dissociate to increase the amount of active core molecules. On the other hand, an unintended increase of core molecules would induce association of core molecules with their inhibitors, decreasing the concentration of active core molecules. Given that ionization equilibrium uses the same type of structure and is known to function as a buffer to stabilize the concentration of a particular ion, this type of complex formation may have a similar role in stabilizing the concentration of components against perturbations. In support of this theory, inhibitors, such as Bmh2 and Bcy1, are much more abundant than their targets (Bmh2:Msn2/4, 51–158:1 and Bcy1:Tpk1/2/3, 4.1–8.9:1),^50^ similar to buffer solutions, in which excess amounts of conjugate base are included. The copy-number limits of Msn2/4 are high (42.8 and 270.8 copies, respectively) compared with those of Tpk1/2/3 (0.9, 2.1, and 0.6 copies, respectively),^44^ suggesting a higher buffer capacity of Bmh2 than Bcy1. The great abundance of Bmh2 compared to its target is one reason for its high buffer capacity. Thus, the ‘reversible complex formation’ motif can be considered as a conserved stabilizer for molecules with a role in bow–tie cores. As shown in Figure 5c, the bow–tie structure, the controllability of a network, and the complex-mediated network motif are closely involved with one another. The importance of the buffering function involving complexes in the robust control of bow–tie cores in yeast stress response pathways is evident from the combined results of these three network analyses.

## Discussion

By virtue of vast numbers of experimental studies, many reactions comprising biological systems have been elucidated in terms of types of reactions and associated molecules. However, how reactions work together to mold a characteristic biological property, such as robustness, remains mostly unclear. This is partially because dispersed knowledge of biological processes makes it difficult to perform systematic analyses. Comprehensive maps built using standardized, computer-readable notations are potent tools for network analyses because they contain multiple layers of information, including protein modification, complex formation, and transportation, which are not included in simple interaction networks such as PPI. The maps can be easily converted into a simplified format suitable for specific analyses by acquiring certain layers of information. Conventional network analyses usually do not take detailed information, such as types of reactions and modifications of molecules, into account. In this study, we integrated detailed, multilayered information derived from the literature to construct maps of yeast stress response networks and used these to perform network analyses. These analyses revealed characteristic features of the networks, demonstrating the effectiveness of incorporating such detailed information.

By careful analysis of the network, we also provide answers to some key questions. Bow–tie scoring was used to quantitatively define bow–tie structures, demonstrating that yeast stress response pathways feature bow–tie architectures. Although bow–tie structures provide robustness against external perturbations, this robustness is entirely dependent on the stability of the core. Our results demonstrate the importance of bow–tie cores in connecting regulators to their targets. This suggests a universal architecture for signal transduction, in which hub molecules, such as bow–tie cores, do not directly regulate their downstream molecules, but act to transmit regulatory signals. This seems logical from a system design perspective as, if cores were regulators, the core molecules would have to change their concentration or activity to achieve regulation and their various targets would drastically change their activity in response to these core fluctuations. As signal transmitters, bow–tie cores need not, and should not, change their concentrations and activities to ensure the stable transmission of signals.

Furthermore, network motif analyses, using a unique labeling method, revealed conserved robust control, mediated by reversible complex formation. Interestingly, a potential role for complex formation in enhancing the robustness of key molecules regulating the cell cycle has been reported.^46^ That study concluded that heterodimer formation and associated regulation, such as phosphorylation, contributed to increased robustness against dose-level perturbations of molecules that would otherwise result in extreme fragility of the cell cycle process. In addition, complex formation and scaffolding were reported to potentially contribute to the robustness of HOG signaling in yeast using *in silico* sensitivity analysis.^51^ Combined with our findings, these data suggest that complex formation by key molecules may be a basic mechanism that contributes to the robustness of cellular functions against unexpected dose changes. This enables signaling bow–tie cores to function as robust, yet flexible, signal mediators that may represent a universal design principle within biological signaling systems.

In addition, we found specific correlations between the results from our controllability analyses and experimental phenotypes caused by genetic perturbations observed in other studies. Although controllability analysis can assess the effect of node failure on network control, whether controllability actually reflects biological properties has not been validated. Interestingly, the controllability of the molecular interaction map correlated closely with synergistic genetic interactions, but not with lethality after deletion of individual genes. Most local loss of function caused by gene deletion or overexpression can be compensated for by other genes with shared functions or alternative pathways, whereas this type of compensation can frequently be disturbed by combinatorial perturbation, such as that caused by synthetic lethality. This indicates that there are substantial numbers of factors that make impacts on the robustness of biological systems, rather than directly on their function. Thus, the controllability of molecular interaction maps, related to combinatorial genetic interactions, can be an index for evaluating biological robustness. As the high-throughput screens for synthetic genetic interactions were undertaken under non-stress condition, other synthetic interactions can appear under specific stress conditions. Specifically, PKC1, SLG1, SKN7 and calcineurin (CNA1, CNB1) were shown to have genetic interactions with numerous other proteins under osmotic stress condition.^52–55^ Interestingly, all of these were critical in our controllability analysis. It is not practical to perform high-throughput synthetic interaction screens for each stress condition. Moreover, in case of mammals, we should consider more complicated conditions. We assume that the controllability analysis based on molecular interaction maps will help to predict such condition-dependent synthetic interactions.

Finally, we would like to emphasize the complementarity of data-driven and knowledge-based approaches in building and utilizing biological networks. Recently, high-throughput omics data have enabled us to predict genetic and physical interactions. The data-driven approach is a very potent tool for implicating novel regulators, revealing unrecognized crosstalk between pathways, and elucidating the overall structures of the network, as shown by a recent study of the salt-responsive signaling network in yeast.^31^ At the same time, as described in this study, knowledge-based approaches have advantages in uncovering regulatory architectures, which consist of exquisite coordination of protein modification, complex formation, and transportation. We will be able to incorporate novel predictions obtained and validated by data-driven approaches into our knowledge-based maps, whereas these will also support data-driven predictions by providing detailed information about interactions and regulatory mechanisms, as demonstrated in the weighted bow–tie analysis. We envisage that comprehensive maps, powered by large-scale omics data and systematic network analyses, will provide a holistic, network-centric framework to organize and interpret the complexity of biological networks.

## Materials and methods

### Comprehensive maps

The maps were built using CellDesigner 4.3.0 (http://celldesigner.org) software, complying with standards SBML^17^ and SBGN.^25^ We employed a top–down approach focusing first on review papers and then on detailed original research articles. For community-based browse and curation, the maps are available at http://www.yeast-maps.org/yeast-stress-response/.

### Bow–tie analyses

Bow–tie scores (b(*m*)∈[0, 1]) were calculated to determine how ‘central’ molecules *m* were in the signaling pathways, as described previously,^27^ with some modifications. We defined external stimuli as source (*S*) and mRNAs as target (*T*). In addition, we used simple paths within 30 lengths from node, *s*, in the source, to node, *t*, in the target. Transcriptome data for the weighted bow–tie analysis were obtained from NCBI Gene Expression Omnibus (GEO; www.ncbi.nlm.nih.gov/geo) using the GEO Series Accession numbers GSE4584, GSE54528, and GSE60613.

### Controllability analyses

Controllability analysis was applied to the maps as described previously.^56^ The minimum set of driver nodes was determined using the Hopcroft–Karp ‘maximum matching’ algorithm.^57^ Next, we identified critical nodes by examining whether the absence of the node requires an increase in the number of driver nodes. Experimental phenotypes, including viability and synthetic lethality, were obtained for each gene from the SGD database (http://www.yeastgenome.org/).

### Network motif analyses

For network motif identification, the maps were converted into bipartite-directed graphs, treating both molecules and reactions as nodes. Edges were labeled with three colors. Six-node network motifs with labeled directed edges were extracted using FANMOD.^58^

Full Methods and any associated references are available in the Supplementary Materials and Methods.

Codes for analysis used in this paper will be made available at https://github.com/eiryo-kawakami/yeast-stress-response.

## Figures and Tables

**Table 1:** Statistical properties of yeast signal transduction maps constructed in this study

	*Ion homeostasis*	*Nutrient adaptation*	*Osmotic stress*	*Oxidative stress*	*Heat shock*	*Pheromone response*
Stimuli	H^+^, Na^+^, K^+^, Li^+^, Ca^2+^, Mg^2+^, Mn^2+^, Cu^2+^, Cd^2+^, Zn^2+^, Fe^3+^, alkali, citric acid, sorbic acid	Glucose, low glucose, amino acids, nitrogen source	Hyper-osmolarility, hypo-osmolarility, cold shock, DMSO, zymolyase	Oxidative stress	Heat shock	Pheromone
Number of species	1,082	580	586	517	481	217
Proteins	368	256	215	167	167	87
Complexes	137	110	79	61	49	71
Genes and RNAs	400	134	228	217	203	40
Other molecules^a^	121 (4)	49 (1)	40 (2)	42 (0)	19 (1)	6 (0)
Number of reactions	768	472	424	464	366	155
Associations and dissociations	105	99	62	83	36	46
State transitions	145	170	111	124	87	51
Transcriptions and translations	284	94	130	132	123	30
Transports	99	59	26	28	20	13
Number of References^b^	281	274	239	253	174	152

Abbreviation: DMSO, dimethyl sulfoxide.

a Other molecules include simple molecules (e.g., ATP, glucose, NADH), ions, and drugs. The number of drugs is indicated in brackets, since drugs are not naturally present in yeast cells.

b A full list of publications referred to in the maps is available in Supplementary Information S1.

**Table 2:** Distribution of bow–tie scores b(*m*) in each stress response pathway

Bow–tie *score b(m)*	*Ion homeostasis*	*Nutrient adaptation*	*Osmotic stress*	*Oxidative stress*	*Heat shock*	*Pheromone response*
0.5–1.0^a^	5 Sho1/Pbs2, Hog1, Pbs2, Hog1/Msn2, Hog1	12 cAMP, EGO complex, Sit4, TORC1, Tap42/TORC1, ATP, Sit4/Tap42/TORC1, fructose 1,6-biphosphate, fructose 6-phosphate, glucose 6-phosphate	6 Hog1/Msn2, Pbs2, Hog1, Sho1/Pbs2	8 Sit4, Msn2, Hyr1/Yap1/Ybp1, Yap1-Ox, Glc7/Reg1, Bmh2	5 cAMP, Cdc25, Cyr1/Srv2, Msn2, Bcy1	23 Cdc24/Far1/Ste4/Ste18, Ste4/Ste18, Bem1/GTP/Cdc42/Cdc24/Far1/Ste4/Ste18, Bem1/GTP/Cdc42/Cdc24/Far1/Ste4/Ste18/Ste20, GTP Gpa1/Ste4/Ste18,Ste5/Ste50/Ste11/Ste7/Kss1, Ste5/Ste50/Ste11/Ste7/Fus3, Ste12, Dig1/Dig2/Ste12, Kss1, a-factor/Ste3, alpha-factor/Ste2
0.2–0.5	3	28	14	30	19	7
<0.2	81	133	67	71	41	2
0	529	325	245	268	190	162
*R*^2^ coefficient^b^	0.3165	0.8881	0.6902	0.5531	0.2243	0.8096

a For molecules with very high bow–tie scores (0.5–1.0), the number of molecules in the map with bow–tie scores b(*m*) within this range are indicated above a list of molecules/complexes with these scores in each map. Molecules in different states (modification or localization) are represented without distinction. Instances separated with '/' indicate complexes.

b *R*^2^ coefficient between bow–tie score and betweenness centrality.
