# Supplementary material for: Network analyses based on comprehensive molecular interaction maps reveal robust control structures in yeast stress response pathways
Source: NPJ Syst Biol Appl. 2016 Jan 7;2:15018–. doi: 10.1038/npjsba.2015.18 (PMC5516916; doi:10.1038/npjsba.2015.18)
Supplement: Supplementary Information S1 [file npjsba201518-s1.doc]

**Bibliography for map construction**

Han S, Lone MA, Schneiter R, Chang A. [Orm1 and Orm2 are conserved endoplasmic reticulum membrane proteins regulating lipid homeostasis and protein quality control.](http://www.ncbi.nlm.nih.gov/pubmed/20212121) *Proc Natl Acad Sci U S A.* 2010; **107:** 5851-6.

Beck F, Unverdorben P, Bohn S, Schweitzer A, Pfeifer G, Sakata E, Nickell S, Plitzko JM, Villa E, Baumeister W, Förster F. [Near-atomic resolution structural model of the yeast 26S proteasome.](http://www.ncbi.nlm.nih.gov/pubmed/22927375) *Proc Natl Acad Sci U S A.* 2012; **109:**14870-5.

Lasker K, Förster F, Bohn S, Walzthoeni T, Villa E, Unverdorben P, Beck F, Aebersold R, Sali A, Baumeister W. [Molecular architecture of the 26S proteasome holocomplex determined by an integrative approach.](http://www.ncbi.nlm.nih.gov/pubmed/22307589) *Proc Natl Acad Sci U S A.*2012; **109:** 1380-7.

Audhya A, Emr SD. [Regulation of PI4,5P2 synthesis by nuclear-cytoplasmic shuttling of the Mss4 lipid kinase.](http://www.ncbi.nlm.nih.gov/pubmed/12912920) *EMBO J.*2003; **22:** 4223-36.

Bose S, Dutko JA, Zitomer RS. [Genetic factors that regulate the attenuation of the general stress response of yeast.](http://www.ncbi.nlm.nih.gov/pubmed/15545648)*Genetics.* 2005; **169:** 1215-26.

Lee P, Paik SM, Shin CS, Huh WK, Hahn JS. [Regulation of yeast Yak1 kinase by PKA and autophosphorylation-dependent 14-3-3 binding.](http://www.ncbi.nlm.nih.gov/pubmed/21255108) *Mol Microbiol.* 2011; **79:** 633-46.

Levin DE. [Regulation of cell wall biogenesis in Saccharomyces cerevisiae: the cell wall integrity signaling pathway.](http://www.ncbi.nlm.nih.gov/pubmed/22174182)*Genetics.* 2011; **189:** 1145-75.

Philip B, Levin DE. [Wsc1 and Mid2 are cell surface sensors for cell wall integrity signaling that act through Rom2, a guanine nucleotide exchange factor for Rho1.](http://www.ncbi.nlm.nih.gov/pubmed/11113201) *Mol Cell Biol.* 2001; **21:** 271-80.

Audhya A, Emr SD. [Stt4 PI 4-kinase localizes to the plasma membrane and functions in the Pkc1-mediated MAP kinase cascade.](http://www.ncbi.nlm.nih.gov/pubmed/12015967) *Dev Cell.* 2002; **2:** 593-605.

Levin DE, Bowers B, Chen CY, Kamada Y, Watanabe M. [Dissecting the protein kinase C/MAP kinase signalling pathway of Saccharomyces cerevisiae.](http://www.ncbi.nlm.nih.gov/pubmed/7874200) *Cell Mol Biol Res.* 1994; **40:** 229-39.

Irie K, Takase M, Lee KS, Levin DE, Araki H, Matsumoto K, Oshima Y. [MKK1 and MKK2, which encode Saccharomyces cerevisiae mitogen-activated protein kinase-kinase homologs, function in the pathway mediated by protein kinase C.](http://www.ncbi.nlm.nih.gov/pubmed/8386320)*Mol Cell Biol.* 1993; **13:** 3076-83.

Kamada Y, Jung US, Piotrowski J, Levin DE. [The protein kinase C-activated MAP kinase pathway of Saccharomyces cerevisiae mediates a novel aspect of the heat shock response.](http://www.ncbi.nlm.nih.gov/pubmed/7628692) *Genes Dev.* 1995; **9:** 1559-71.

Paravicini G, Friedli L. [Protein-protein interactions in the yeast PKC1 pathway: Pkc1p interacts with a component of the MAP kinase cascade.](http://www.ncbi.nlm.nih.gov/pubmed/8757399) *Mol Gen Genet.* 1996; **251:** 682-91.

Martín H, Rodríguez-Pachón JM, Ruiz C, Nombela C, Molina M. [Regulatory mechanisms for modulation of signaling through the cell integrity Slt2-mediated pathway in Saccharomyces cerevisiae.](http://www.ncbi.nlm.nih.gov/pubmed/10625705) *J Biol Chem.* 2000; **275:** 1511-9.

Levin DE. [Cell wall integrity signaling in Saccharomyces cerevisiae.](http://www.ncbi.nlm.nih.gov/pubmed/15944456) *Microbiol Mol Biol Rev.* 2005; **69:** 262-91.

Peterson J, Zheng Y, Bender L, Myers A, Cerione R, Bender A. [Interactions between the bud emergence proteins Bem1p and Bem2p and Rho-type GTPases in yeast.](http://www.ncbi.nlm.nih.gov/pubmed/7962098) *J Cell Biol.* 1994; **127:** 1395-406.

Martín H, Flández M, Nombela C, Molina M. [Protein phosphatases in MAPK signalling: we keep learning from yeast.](http://www.ncbi.nlm.nih.gov/pubmed/16164545) *Mol Microbiol.* 2005; **58:** 6-16.

Mattison CP, Spencer SS, Kresge KA, Lee J, Ota IM. [Differential regulation of the cell wall integrity mitogen-activated protein kinase pathway in budding yeast by the protein tyrosine phosphatases Ptp2 and Ptp3.](http://www.ncbi.nlm.nih.gov/pubmed/10523653) *Mol Cell Biol.* 1999; **19:**7651-60.

Hahn JS, Thiele DJ. [Regulation of the Saccharomyces cerevisiae Slt2 kinase pathway by the stress-inducible Sdp1 dual specificity phosphatase.](http://www.ncbi.nlm.nih.gov/pubmed/11923319) *J Biol Chem.* 2002; **277:** 21278-84.

Flández M, Cosano IC, Nombela C, Martín H, Molina M. [Reciprocal regulation between Slt2 MAPK and isoforms of Msg5 dual-specificity protein phosphatase modulates the yeast cell integrity pathway.](http://www.ncbi.nlm.nih.gov/pubmed/14703512) *J Biol Chem.* 2004; **279:** 11027-34.

Hashikawa N, Mizukami Y, Imazu H, Sakurai H. [Mutated yeast heat shock transcription factor activates transcription independently of hyperphosphorylation.](http://www.ncbi.nlm.nih.gov/pubmed/16361698) *J Biol Chem.* 2006; **281:** 3936-42.

Yamamoto N, Maeda Y, Ikeda A, Sakurai H. [Regulation of thermotolerance by stress-induced transcription factors in Saccharomyces cerevisiae.](http://www.ncbi.nlm.nih.gov/pubmed/18359875) *Eukaryot Cell.* 2008; **7:** 783-90.

Audhya A, Loewith R, Parsons AB, Gao L, Tabuchi M, Zhou H, Boone C, Hall MN, Emr SD. [Genome-wide lethality screen identifies new PI4,5P2 effectors that regulate the actin cytoskeleton.](http://www.ncbi.nlm.nih.gov/pubmed/15372071) *EMBO J.* 2004; **23:** 3747-57.

Fadri M, Daquinag A, Wang S, Xue T, Kunz J. [The pleckstrin homology domain proteins Slm1 and Slm2 are required for actin cytoskeleton organization in yeast and bind phosphatidylinositol-4,5-bisphosphate and TORC2.](http://www.ncbi.nlm.nih.gov/pubmed/15689497) *Mol Biol Cell.*2005; **16:** 1883-900.

Roelants FM, Torrance PD, Bezman N, Thorner J. [Pkh1 and Pkh2 differentially phosphorylate and activate Ypk1 and Ykr2 and define protein kinase modules required for maintenance of cell wall integrity.](http://www.ncbi.nlm.nih.gov/pubmed/12221112) *Mol Biol Cell.* 2002; **13:** 3005-28.

Kamada Y, Fujioka Y, Suzuki NN, Inagaki F, Wullschleger S, Loewith R, Hall MN, Ohsumi Y. [Tor2 directly phosphorylates the AGC kinase Ypk2 to regulate actin polarization.](http://www.ncbi.nlm.nih.gov/pubmed/16055732) *Mol Cell Biol.* 2005; **25:** 7239-48.

Niles BJ, Mogri H, Hill A, Vlahakis A, Powers T. [Plasma membrane recruitment and activation of the AGC kinase Ypk1 is mediated by target of rapamycin complex 2 (TORC2) and its effector proteins Slm1 and Slm2.](http://www.ncbi.nlm.nih.gov/pubmed/22307609) *Proc Natl Acad Sci U S A.* 2012; **109:** 1536-41.

Roelants FM, Breslow DK, Muir A, Weissman JS, Thorner J. [Protein kinase Ypk1 phosphorylates regulatory proteins Orm1 and Orm2 to control sphingolipid homeostasis in Saccharomyces cerevisiae.](http://www.ncbi.nlm.nih.gov/pubmed/22080611) *Proc Natl Acad Sci U S A.* 2011; **108:**19222-7.

Sun Y, Miao Y, Yamane Y, Zhang C, Shokat KM, Takematsu H, Kozutsumi Y, Drubin DG. [Orm protein phosphoregulation mediates transient sphingolipid biosynthesis response to heat stress via the Pkh-Ypk and Cdc55-PP2A pathways.](http://www.ncbi.nlm.nih.gov/pubmed/22535525) *Mol Biol Cell.* 2012; **23:** 2388-98.

Galan JM, Haguenauer-Tsapis R. [Ubiquitin lys63 is involved in ubiquitination of a yeast plasma membrane protein.](http://www.ncbi.nlm.nih.gov/pubmed/9312043) *EMBO J.* 1997; **16:** 5847-54.

Galan JM, Moreau V, Andre B, Volland C, Haguenauer-Tsapis R. [Ubiquitination mediated by the Npi1p/Rsp5p ubiquitin-protein ligase is required for endocytosis of the yeast uracil permease.](http://www.ncbi.nlm.nih.gov/pubmed/8631913) *J Biol Chem.* 1996; **271:** 10946-52.

Volland C, Urban-Grimal D, Géraud G, Haguenauer-Tsapis R. [Endocytosis and degradation of the yeast uracil permease under adverse conditions.](http://www.ncbi.nlm.nih.gov/pubmed/8144575) *J Biol Chem.* 1994; **269:** 9833-41.

Chung N, Mao C, Heitman J, Hannun YA, Obeid LM. [Phytosphingosine as a specific inhibitor of growth and nutrient import in Saccharomyces cerevisiae.](http://www.ncbi.nlm.nih.gov/pubmed/11468289) *J Biol Chem.* 2001; **276:** 35614-21.

Dickson RC. [Thematic review series: sphingolipids. New insights into sphingolipid metabolism and function in budding yeast.](http://www.ncbi.nlm.nih.gov/pubmed/18296751)*J Lipid Res.* 2008; **49:** 909-21.

Hearn JD, Lester RL, Dickson RC. [The uracil transporter Fur4p associates with lipid rafts.](http://www.ncbi.nlm.nih.gov/pubmed/12446707) *J Biol Chem.* 2003; **278:** 3679-86.

Chung N, Jenkins G, Hannun YA, Heitman J, Obeid LM. [Sphingolipids signal heat stress-induced ubiquitin-dependent proteolysis.](http://www.ncbi.nlm.nih.gov/pubmed/10764732) *J Biol Chem.* 2000; **275:** 17229-32.

Kamada Y, Qadota H, Python CP, Anraku Y, Ohya Y, Levin DE. [Activation of yeast protein kinase C by Rho1 GTPase.](http://www.ncbi.nlm.nih.gov/pubmed/8621575) *J Biol Chem.* 1996; **271:** 9193-6.

Inagaki M, Schmelzle T, Yamaguchi K, Irie K, Hall MN, Matsumoto K. [PDK1 homologs activate the Pkc1-mitogen-activated protein kinase pathway in yeast.](http://www.ncbi.nlm.nih.gov/pubmed/10567559) *Mol Cell Biol.* 1999; **19:** 8344-52.

Friant S, Lombardi R, Schmelzle T, Hall MN, Riezman H. [Sphingoid base signaling via Pkh kinases is required for endocytosis in yeast.](http://www.ncbi.nlm.nih.gov/pubmed/11726514) *EMBO J.* 2001; **20:** 6783-92.

Kim KY, Truman AW, Levin DE. [Yeast Mpk1 mitogen-activated protein kinase activates transcription through Swi4/Swi6 by a noncatalytic mechanism that requires upstream signal.](http://www.ncbi.nlm.nih.gov/pubmed/18268013) *Mol Cell Biol.* 2008; **28:** 2579-89.

Morano KA, Grant CM, Moye-Rowley WS. [The response to heat shock and oxidative stress in Saccharomyces cerevisiae.](http://www.ncbi.nlm.nih.gov/pubmed/22209905)*Genetics.* 2012; **190:** 1157-95.

Lee P, Kim MS, Paik SM, Choi SH, Cho BR, Hahn JS. [Rim15-dependent activation of Hsf1 and Msn2/4 transcription factors by direct phosphorylation in Saccharomyces cerevisiae.](http://www.ncbi.nlm.nih.gov/pubmed/24140345) *FEBS Lett.* 2013; **587:** 3648-55.

Duina AA, Kalton HM, Gaber RF. [Requirement for Hsp90 and a CyP-40-type cyclophilin in negative regulation of the heat shock response.](http://www.ncbi.nlm.nih.gov/pubmed/9668076) *J Biol Chem.* 1998; **273:** 18974-8.

Harris N, MacLean M, Hatzianthis K, Panaretou B, Piper PW. [Increasing Saccharomyces cerevisiae stress resistance, through the overactivation of the heat shock response resulting from defects in the Hsp90 chaperone, does not extend replicative life span but can be associated with slower chronological ageing of nondividing cells.](http://www.ncbi.nlm.nih.gov/pubmed/11361336) *Mol Genet Genomics.* 2001; **265:** 258-63.

Batista-Nascimento L, Neef DW, Liu PC, Rodrigues-Pousada C, Thiele DJ. [Deciphering human heat shock transcription factor 1 regulation via post-translational modification in yeast.](http://www.ncbi.nlm.nih.gov/pubmed/21253609) *PLoS One.* 2011; **6:** e15976.

Zou J, Guo Y, Guettouche T, Smith DF, Voellmy R. [Repression of heat shock transcription factor HSF1 activation by HSP90 (HSP90 complex) that forms a stress-sensitive complex with HSF1.](http://www.ncbi.nlm.nih.gov/pubmed/9727490) *Cell.* 1998; **94:** 471-80.

Shi Y, Mosser DD, Morimoto RI. [Molecular chaperones as HSF1-specific transcriptional repressors.](http://www.ncbi.nlm.nih.gov/pubmed/9499401) *Genes Dev.* 1998; **12:**654-66.

Valdivia RH, Schekman R. [The yeasts Rho1p and Pkc1p regulate the transport of chitin synthase III (Chs3p) from internal stores to the plasma membrane.](http://www.ncbi.nlm.nih.gov/pubmed/12928491) *Proc Natl Acad Sci U S A.* 2003; **100:** 10287-92.

Reyes A, Sanz M, Duran A, Roncero C. [Chitin synthase III requires Chs4p-dependent translocation of Chs3p into the plasma membrane.](http://www.ncbi.nlm.nih.gov/pubmed/17519287) *J Cell Sci.* 2007; **120:** 1998-2009.

Amorós M, Estruch F. [Hsf1p and Msn2/4p cooperate in the expression of Saccharomyces cerevisiae genes HSP26 and HSP104 in a gene- and stress type-dependent manner.](http://www.ncbi.nlm.nih.gov/pubmed/11260469) *Mol Microbiol.* 2001; **39:** 1523-32.

Estruch F. [Stress-controlled transcription factors, stress-induced genes and stress tolerance in budding yeast.](http://www.ncbi.nlm.nih.gov/pubmed/10978547) *FEMS Microbiol Rev.* 2000; **24:** 469-86.

Tamai KT, Liu X, Silar P, Sosinowski T, Thiele DJ. [Heat shock transcription factor activates yeast metallothionein gene expression in response to heat and glucose starvation via distinct signalling pathways.](http://www.ncbi.nlm.nih.gov/pubmed/7969152) *Mol Cell Biol.* 1994; **14:** 8155-65.

Hahn JS, Thiele DJ. [Activation of the Saccharomyces cerevisiae heat shock transcription factor under glucose starvation conditions by Snf1 protein kinase.](http://www.ncbi.nlm.nih.gov/pubmed/14612437) *J Biol Chem.* 2004; **279:** 5169-76.

Owsianik G, Balzi l L, Ghislain M. [Control of 26S proteasome expression by transcription factors regulating multidrug resistance in Saccharomyces cerevisiae.](http://www.ncbi.nlm.nih.gov/pubmed/11918814) *Mol Microbiol.* 2002; **43:** 1295-308.

Yokoyama H, Mizunuma M, Okamoto M, Yamamoto J, Hirata D, Miyakawa T. [Involvement of calcineurin-dependent degradation of Yap1p in Ca2+-induced G2 cell-cycle regulation in Saccharomyces cerevisiae.](http://www.ncbi.nlm.nih.gov/pubmed/16485023) *EMBO Rep.* 2006; **7:**519-24.

Hahn JS, Neef DW, Thiele DJ. [A stress regulatory network for co-ordinated activation of proteasome expression mediated by yeast heat shock transcription factor.](http://www.ncbi.nlm.nih.gov/pubmed/16556235) *Mol Microbiol.* 2006; **60:** 240-51.

Ruiz-Roig C, Viéitez C, Posas F, de Nadal E. [The Rpd3L HDAC complex is essential for the heat stress response in yeast.](http://www.ncbi.nlm.nih.gov/pubmed/20398213)*Mol Microbiol.* 2010; **76:** 1049-62.

Raitt DC, Johnson AL, Erkine AM, Makino K, Morgan B, Gross DS, Johnston LH. [The Skn7 response regulator of Saccharomyces cerevisiae interacts with Hsf1 in vivo and is required for the induction of heat shock genes by oxidative stress.](http://www.ncbi.nlm.nih.gov/pubmed/10888672) *Mol Biol Cell.* 2000; **11:** 2335-47.

Wanke V, Cameroni E, Uotila A, Piccolis M, Urban J, Loewith R, De Virgilio C. [Caffeine extends yeast lifespan by targeting TORC1.](http://www.ncbi.nlm.nih.gov/pubmed/18513215) *Mol Microbiol.* 2008; **69:** 277-85.

Bultynck G, Heath VL, Majeed AP, Galan JM, Haguenauer-Tsapis R, Cyert MS. [Slm1 and slm2 are novel substrates of the calcineurin phosphatase required for heat stress-induced endocytosis of the yeast uracil permease.](http://www.ncbi.nlm.nih.gov/pubmed/16738335) *Mol Cell Biol.*2006; **26:** 4729-45.

Pracheil T, Thornton J, Liu Z. [TORC2 signaling is antagonized by protein phosphatase 2A and the Far complex in Saccharomyces cerevisiae.](http://www.ncbi.nlm.nih.gov/pubmed/22298706) *Genetics.* 2012; **190:** 1325-39.

Luo G, Gruhler A, Liu Y, Jensen ON, Dickson RC. [The sphingolipid long-chain base-Pkh1/2-Ypk1/2 signaling pathway regulates eisosome assembly and turnover.](http://www.ncbi.nlm.nih.gov/pubmed/18296441) *J Biol Chem.* 2008; **283:** 10433-44.

Pagán-Mercado G, Santiago-Cartagena E, Akamine P, Rodríguez-Medina JR. [Functional and genetic interactions of TOR in the budding yeast Saccharomyces cerevisiae with myosin type II-deficiency (myo1Δ).](http://www.ncbi.nlm.nih.gov/pubmed/22646158) *BMC Cell Biol.* 2012; **13:** 13.

Erkina TY, Tschetter PA, Erkine AM. [Different requirements of the SWI/SNF complex for robust nucleosome displacement at promoters of heat shock factor and Msn2- and Msn4-regulated heat shock genes.](http://www.ncbi.nlm.nih.gov/pubmed/18070923) *Mol Cell Biol.* 2008;**28:** 1207-17.

Sadeh A, Movshovich N, Volokh M, Gheber L, Aharoni A. [Fine-tuning of the Msn2/4-mediated yeast stress responses as revealed by systematic deletion of Msn2/4 partners.](http://www.ncbi.nlm.nih.gov/pubmed/21757539) *Mol Biol Cell.* 2011; **22:** 3127-38.

Qadota H, Python CP, Inoue SB, Arisawa M, Anraku Y, Zheng Y, Watanabe T, Levin DE, Ohya Y. [Identification of yeast Rho1p GTPase as a regulatory subunit of 1,3-beta-glucan synthase.](http://www.ncbi.nlm.nih.gov/pubmed/8602515) *Science.* 1996; **272:** 279-81.

Jiménez-Sánchez M, Cid VJ, Molina M. [Retrophosphorylation of Mkk1 and Mkk2 MAPKKs by the Slt2 MAPK in the yeast cell integrity pathway.](http://www.ncbi.nlm.nih.gov/pubmed/17711850) *J Biol Chem.* 2007; **282:** 31174-85.

Chi Y, Huddleston MJ, Zhang X, Young RA, Annan RS, Carr SA, Deshaies RJ. [Negative regulation of Gcn4 and Msn2 transcription factors by Srb10 cyclin-dependent kinase.](http://www.ncbi.nlm.nih.gov/pubmed/11331604) *Genes Dev.* 2001; **15:** 1078-92.

Zhu Y, Xiao W. [Pdr3 is required for DNA damage induction of MAG1 and DDI1 via a bi-directional promoter element.](http://www.ncbi.nlm.nih.gov/pubmed/15452273)*Nucleic Acids Res.* 2004; **32:** 5066-75.

Liu XD, Morano KA, Thiele DJ. [The yeast Hsp110 family member, Sse1, is an Hsp90 cochaperone.](http://www.ncbi.nlm.nih.gov/pubmed/10480867) *J Biol Chem.* 1999;**274:** 26654-60.

Eastmond DL, Nelson HC. [Genome-wide analysis reveals new roles for the activation domains of the Saccharomyces cerevisiae heat shock transcription factor (Hsf1) during the transient heat shock response.](http://www.ncbi.nlm.nih.gov/pubmed/16926161) *J Biol Chem.* 2006; **281:**32909-21.

Sakurai H, Ota A. [Regulation of chaperone gene expression by heat shock transcription factor in Saccharomyces cerevisiae: importance in normal cell growth, stress resistance, and longevity.](http://www.ncbi.nlm.nih.gov/pubmed/21827755) *FEBS Lett.* 2011; **585:** 2744-8.

Truman AW, Millson SH, Nuttall JM, Mollapour M, Prodromou C, Piper PW. [In the yeast heat shock response, Hsf1-directed induction of Hsp90 facilitates the activation of the Slt2 (Mpk1) mitogen-activated protein kinase required for cell integrity.](http://www.ncbi.nlm.nih.gov/pubmed/17293484) *Eukaryot Cell.* 2007; **6:** 744-52.

Lee CT, Graf C, Mayer FJ, Richter SM, Mayer MP. [Dynamics of the regulation of Hsp90 by the co-chaperone Sti1.](http://www.ncbi.nlm.nih.gov/pubmed/22354036)*EMBO J.* 2012; **31:** 1518-28.

Schmid AB, Lagleder S, Gräwert MA, Röhl A, Hagn F, Wandinger SK, Cox MB, Demmer O, Richter K, Groll M, Kessler H, Buchner J. [The architecture of functional modules in the Hsp90 co-chaperone Sti1/Hop.](http://www.ncbi.nlm.nih.gov/pubmed/22227520) *EMBO J.* 2012; **31:** 1506-17.

Lee P, Shabbir A, Cardozo C, Caplan AJ. [Sti1 and Cdc37 can stabilize Hsp90 in chaperone complexes with a protein kinase.](http://www.ncbi.nlm.nih.gov/pubmed/14742721) *Mol Biol Cell.* 2004; **15:** 1785-92.

Boy-Marcotte E, Lagniel G, Perrot M, Bussereau F, Boudsocq A, Jacquet M, Labarre J. [The heat shock response in yeast: differential regulations and contributions of the Msn2p/Msn4p and Hsf1p regulons.](http://www.ncbi.nlm.nih.gov/pubmed/10411744) *Mol Microbiol.* 1999; **33:** 274-83.

Panaretou B, Siligardi G, Meyer P, Maloney A, Sullivan JK, Singh S, Millson SH, Clarke PA, Naaby-Hansen S, Stein R, Cramer R, Mollapour M, Workman P, Piper PW, Pearl LH, Prodromou C. [Activation of the ATPase activity of hsp90 by the stress-regulated cochaperone aha1.](http://www.ncbi.nlm.nih.gov/pubmed/12504007) *Mol Cell.* 2002; **10:** 1307-18.

Zuehlke AD, Johnson JL. [Chaperoning the chaperone: a role for the co-chaperone Cpr7 in modulating Hsp90 function in Saccharomyces cerevisiae.](http://www.ncbi.nlm.nih.gov/pubmed/22505624) *Genetics.* 2012; **191:** 805-14.

Zarzov P, Boucherie H, Mann C. [A yeast heat shock transcription factor (Hsf1) mutant is defective in both Hsc82/Hsp82 synthesis and spindle pole body duplication.](http://www.ncbi.nlm.nih.gov/pubmed/9296388) *J Cell Sci.* 1997; **110 ( Pt 16):** 1879-91.

Mollapour M, Tsutsumi S, Donnelly AC, Beebe K, Tokita MJ, Lee MJ, Lee S, Morra G, Bourboulia D, Scroggins BT, Colombo G, Blagg BS, Panaretou B, Stetler-Stevenson WG, Trepel JB, Piper PW, Prodromou C, Pearl LH, Neckers L.[Swe1Wee1-dependent tyrosine phosphorylation of Hsp90 regulates distinct facets of chaperone function.](http://www.ncbi.nlm.nih.gov/pubmed/20159553) *Mol Cell.*2010; **37:** 333-43.

Aligue R, Akhavan-Niak H, Russell P. [A role for Hsp90 in cell cycle control: Wee1 tyrosine kinase activity requires interaction with Hsp90.](http://www.ncbi.nlm.nih.gov/pubmed/7813446) *EMBO J.* 1994; **13:** 6099-106.

Wandinger SK, Suhre MH, Wegele H, Buchner J. [The phosphatase Ppt1 is a dedicated regulator of the molecular chaperone Hsp90.](http://www.ncbi.nlm.nih.gov/pubmed/16407978) *EMBO J.* 2006; **25:** 367-76.

Louvion JF, Abbas-Terki T, Picard D. [Hsp90 is required for pheromone signaling in yeast.](http://www.ncbi.nlm.nih.gov/pubmed/9802897) *Mol Biol Cell.* 1998; **9:** 3071-83.

Imai J, Yahara I. [Role of HSP90 in salt stress tolerance via stabilization and regulation of calcineurin.](http://www.ncbi.nlm.nih.gov/pubmed/11094077) *Mol Cell Biol.* 2000;**20:** 9262-70.

Liou ST, Cheng MY, Wang C. [SGT2 and MDY2 interact with molecular chaperone YDJ1 in Saccharomyces cerevisiae.](http://www.ncbi.nlm.nih.gov/pubmed/17441508) *Cell Stress Chaperones.* 2007; **12:** 59-70.

Mandal AK, Nillegoda NB, Chen JA, Caplan AJ. [Ydj1 protects nascent protein kinases from degradation and controls the rate of their maturation.](http://www.ncbi.nlm.nih.gov/pubmed/18443039) *Mol Cell Biol.* 2008; **28:** 4434-44.

Lenssen E, James N, Pedruzzi I, Dubouloz F, Cameroni E, Bisig R, Maillet L, Werner M, Roosen J, Petrovic K, Winderickx J, Collart MA, De Virgilio C. [The Ccr4-Not complex independently controls both Msn2-dependent transcriptional activation--via a newly identified Glc7/Bud14 type I protein phosphatase module--and TFIID promoter distribution.](http://www.ncbi.nlm.nih.gov/pubmed/15601868)*Mol Cell Biol.* 2005; **25:** 488-98.

Jacquet M, Renault G, Lallet S, De Mey J, Goldbeter A. [Oscillatory nucleocytoplasmic shuttling of the general stress response transcriptional activators Msn2 and Msn4 in Saccharomyces cerevisiae.](http://www.ncbi.nlm.nih.gov/pubmed/12732613) *J Cell Biol.* 2003; **161:** 497-505.

Schade B, Jansen G, Whiteway M, Entian KD, Thomas DY. [Cold adaptation in budding yeast.](http://www.ncbi.nlm.nih.gov/pubmed/15483057) *Mol Biol Cell.* 2004; **15:**5492-502.

Zähringer H, Thevelein JM, Nwaka S. [Induction of neutral trehalase Nth1 by heat and osmotic stress is controlled by STRE elements and Msn2/Msn4 transcription factors: variations of PKA effect during stress and growth.](http://www.ncbi.nlm.nih.gov/pubmed/10652100) *Mol Microbiol.* 2000; **35:** 397-406.

Mahmud SA, Hirasawa T, Shimizu H. [Differential importance of trehalose accumulation in Saccharomyces cerevisiae in response to various environmental stresses.](http://www.ncbi.nlm.nih.gov/pubmed/20159575) *J Biosci Bioeng.* 2010; **109:** 262-6.

Lv Y, Xiao D, He D, Guo X. [[Construction and stress tolerance of trehalase mutant in Saccharomyces cerevisiae].](http://www.ncbi.nlm.nih.gov/pubmed/19160808) *Wei Sheng Wu Xue Bao.* 2008; **48:** 1301-7.

De Virgilio C, Hottiger T, Dominguez J, Boller T, Wiemken A. [The role of trehalose synthesis for the acquisition of thermotolerance in yeast. I. Genetic evidence that trehalose is a thermoprotectant.](http://www.ncbi.nlm.nih.gov/pubmed/8306984) *Eur J Biochem.* 1994; **219:** 179-86.

Zähringer H, Burgert M, Holzer H, Nwaka S. [Neutral trehalase Nth1p of Saccharomyces cerevisiae encoded by the NTH1 gene is a multiple stress responsive protein.](http://www.ncbi.nlm.nih.gov/pubmed/9276477) *FEBS Lett.* 1997; **412:** 615-20.

Bell W, Sun W, Hohmann S, Wera S, Reinders A, De Virgilio C, Wiemken A, Thevelein JM. [Composition and functional analysis of the Saccharomyces cerevisiae trehalose synthase complex.](http://www.ncbi.nlm.nih.gov/pubmed/9837904) *J Biol Chem.* 1998; **273:** 33311-9.

Zähringer H, Holzer H, Nwaka S. [Stability of neutral trehalase during heat stress in Saccharomyces cerevisiae is dependent on the activity of the catalytic subunits of cAMP-dependent protein kinase, Tpk1 and Tpk2.](http://www.ncbi.nlm.nih.gov/pubmed/9738892) *Eur J Biochem.* 1998; **255:** 544-51.

Lan C, Lee HC, Tang S, Zhang L. [A novel mode of chaperone action: heme activation of Hap1 by enhanced association of Hsp90 with the repressed Hsp70-Hap1 complex.](http://www.ncbi.nlm.nih.gov/pubmed/15102838) *J Biol Chem.* 2004; **279:** 27607-12.

Zhang L, Hach A, Wang C. [Molecular mechanism governing heme signaling in yeast: a higher-order complex mediates heme regulation of the transcriptional activator HAP1.](http://www.ncbi.nlm.nih.gov/pubmed/9632766) *Mol Cell Biol.* 1998; **18:** 3819-28.

Hon T, Lee HC, Hach A, Johnson JL, Craig EA, Erdjument-Bromage H, Tempst P, Zhang L. [The Hsp70-Ydj1 molecular chaperone represses the activity of the heme activator protein Hap1 in the absence of heme.](http://www.ncbi.nlm.nih.gov/pubmed/11689685) *Mol Cell Biol.* 2001; **21:**7923-32.

Cyert MS. [Genetic analysis of calmodulin and its targets in Saccharomyces cerevisiae.](http://www.ncbi.nlm.nih.gov/pubmed/11700296) *Annu Rev Genet.* 2001; **35:** 647-72.

Roy J, Li H, Hogan PG, Cyert MS. [A conserved docking site modulates substrate affinity for calcineurin, signaling output, and in vivo function.](http://www.ncbi.nlm.nih.gov/pubmed/17386265) *Mol Cell.* 2007; **25:** 889-901.

Cyert MS, Philpott CC. [Regulation of cation balance in Saccharomyces cerevisiae.](http://www.ncbi.nlm.nih.gov/pubmed/23463800) *Genetics.* 2013; **193:** 677-713.

Conde R, Xavier J, McLoughlin C, Chinkers M, Ovsenek N. [Protein phosphatase 5 is a negative modulator of heat shock factor 1.](http://www.ncbi.nlm.nih.gov/pubmed/15967796) *J Biol Chem.* 2005; **280:** 28989-96.

Zu T, Verna J, Ballester R. [Mutations in WSC genes for putative stress receptors result in sensitivity to multiple stress conditions and impairment of Rlm1-dependent gene expression in Saccharomyces cerevisiae.](http://www.ncbi.nlm.nih.gov/pubmed/11589572) *Mol Genet Genomics.*2001; **266:** 142-55.

Green R, Lesage G, Sdicu AM, Ménard P, Bussey H. [A synthetic analysis of the Saccharomyces cerevisiae stress sensor Mid2p, and identification of a Mid2p-interacting protein, Zeo1p, that modulates the PKC1-MPK1 cell integrity pathway.](http://www.ncbi.nlm.nih.gov/pubmed/12949174)*Microbiology.* 2003; **149:** 2487-99.

Imazu H, Sakurai H. [Saccharomyces cerevisiae heat shock transcription factor regulates cell wall remodeling in response to heat shock.](http://www.ncbi.nlm.nih.gov/pubmed/15947197) *Eukaryot Cell.* 2005; **4:** 1050-6.

Samantaray S, Neubauer M, Helmschrott C, Wagener J. [Role of the guanine nucleotide exchange factor Rom2 in cell wall integrity maintenance of Aspergillus fumigatus.](http://www.ncbi.nlm.nih.gov/pubmed/23264643) *Eukaryot Cell.* 2013; **12:** 288-98.

Moseley JB, Maiti S, Goode BL. [Formin proteins: purification and measurement of effects on actin assembly.](http://www.ncbi.nlm.nih.gov/pubmed/16472660) *Methods Enzymol.* 2006; **406:** 215-34.

Tolliday N, VerPlank L, Li R. [Rho1 directs formin-mediated actin ring assembly during budding yeast cytokinesis.](http://www.ncbi.nlm.nih.gov/pubmed/12419188) *Curr Biol.*2002; **12:** 1864-70.

Guo W, Tamanoi F, Novick P. [Spatial regulation of the exocyst complex by Rho1 GTPase.](http://www.ncbi.nlm.nih.gov/pubmed/11283608) *Nat Cell Biol.* 2001; **3:** 353-60.

Pratt ZL, Drehman BJ, Miller ME, Johnston SD. [Mutual interdependence of MSI1 (CAC3) and YAK1 in Saccharomyces cerevisiae.](http://www.ncbi.nlm.nih.gov/pubmed/17321547) *J Mol Biol.* 2007; **368:** 30-43.

Toda T, Cameron S, Sass P, Zoller M, Wigler M. [Three different genes in S. cerevisiae encode the catalytic subunits of the cAMP-dependent protein kinase.](http://www.ncbi.nlm.nih.gov/pubmed/3036373) *Cell.* 1987; **50:** 277-87.

Budhwar R, Lu A, Hirsch JP. [Nutrient control of yeast PKA activity involves opposing effects on phosphorylation of the Bcy1 regulatory subunit.](http://www.ncbi.nlm.nih.gov/pubmed/20826609) *Mol Biol Cell.* 2010; **21:** 3749-58.

Mao C, Wadleigh M, Jenkins GM, Hannun YA, Obeid LM. [Identification and characterization of Saccharomyces cerevisiae dihydrosphingosine-1-phosphate phosphatase.](http://www.ncbi.nlm.nih.gov/pubmed/9353337) *J Biol Chem.* 1997; **272:** 28690-4.

Mandala SM, Thornton R, Tu Z, Kurtz MB, Nickels J, Broach J, Menzeleev R, Spiegel S. [Sphingoid base 1-phosphate phosphatase: a key regulator of sphingolipid metabolism and stress response.](http://www.ncbi.nlm.nih.gov/pubmed/9419344) *Proc Natl Acad Sci U S A.* 1998; **95:**150-5.

Nagiec MM, Skrzypek M, Nagiec EE, Lester RL, Dickson RC. [The LCB4 (YOR171c) and LCB5 (YLR260w) genes of Saccharomyces encode sphingoid long chain base kinases.](http://www.ncbi.nlm.nih.gov/pubmed/9677363) *J Biol Chem.* 1998; **273:** 19437-42.

Saba JD, Nara F, Bielawska A, Garrett S, Hannun YA. [The BST1 gene of Saccharomyces cerevisiae is the sphingosine-1-phosphate lyase.](http://www.ncbi.nlm.nih.gov/pubmed/9334171) *J Biol Chem.* 1997; **272:** 26087-90.

Buede R, Rinker-Schaffer C, Pinto WJ, Lester RL, Dickson RC. [Cloning and characterization of LCB1, a Saccharomyces gene required for biosynthesis of the long-chain base component of sphingolipids.](http://www.ncbi.nlm.nih.gov/pubmed/2066332) *J Bacteriol.* 1991; **173:** 4325-32.

Nagiec MM, Baltisberger JA, Wells GB, Lester RL, Dickson RC. [The LCB2 gene of Saccharomyces and the related LCB1 gene encode subunits of serine palmitoyltransferase, the initial enzyme in sphingolipid synthesis.](http://www.ncbi.nlm.nih.gov/pubmed/8058731) *Proc Natl Acad Sci U S A.* 1994; **91:** 7899-902.

Beeler T, Bacikova D, Gable K, Hopkins L, Johnson C, Slife H, Dunn T. [The Saccharomyces cerevisiae TSC10/YBR265w gene encoding 3-ketosphinganine reductase is identified in a screen for temperature-sensitive suppressors of the Ca2+-sensitive csg2Delta mutant.](http://www.ncbi.nlm.nih.gov/pubmed/9804843) *J Biol Chem.* 1998; **273:** 30688-94.

Perry DK. [Serine palmitoyltransferase: role in apoptotic de novo ceramide synthesis and other stress responses.](http://www.ncbi.nlm.nih.gov/pubmed/12531548) *Biochim Biophys Acta.* 2002; **1585:** 146-52.

Hanada K, Hara T, Nishijima M. [Purification of the serine palmitoyltransferase complex responsible for sphingoid base synthesis by using affinity peptide chromatography techniques.](http://www.ncbi.nlm.nih.gov/pubmed/10722674) *J Biol Chem.* 2000; **275:** 8409-15.

Gable K, Han G, Monaghan E, Bacikova D, Natarajan M, Williams R, Dunn TM. [Mutations in the yeast LCB1 and LCB2 genes, including those corresponding to the hereditary sensory neuropathy type I mutations, dominantly inactivate serine palmitoyltransferase.](http://www.ncbi.nlm.nih.gov/pubmed/11781309) *J Biol Chem.* 2002; **277:** 10194-200.

Breslow DK, Collins SR, Bodenmiller B, Aebersold R, Simons K, Shevchenko A, Ejsing CS, Weissman JS. [Orm family proteins mediate sphingolipid homeostasis.](http://www.ncbi.nlm.nih.gov/pubmed/20182505) *Nature.* 2010; **463:** 1048-53.

Roelants FM, Torrance PD, Thorner J. [Differential roles of PDK1- and PDK2-phosphorylation sites in the yeast AGC kinases Ypk1, Pkc1 and Sch9.](http://www.ncbi.nlm.nih.gov/pubmed/15470109) *Microbiology.* 2004; **150:** 3289-304.

Zhang X, Lester RL, Dickson RC. [Pil1p and Lsp1p negatively regulate the 3-phosphoinositide-dependent protein kinase-like kinase Pkh1p and downstream signaling pathways Pkc1p and Ypk1p.](http://www.ncbi.nlm.nih.gov/pubmed/15016821) *J Biol Chem.* 2004; **279:** 22030-8.

Boustany LM, Cyert MS. [Calcineurin-dependent regulation of Crz1p nuclear export requires Msn5p and a conserved calcineurin docking site.](http://www.ncbi.nlm.nih.gov/pubmed/11877380) *Genes Dev.* 2002; **16:** 608-19.

Kafadar KA, Zhu H, Snyder M, Cyert MS. [Negative regulation of calcineurin signaling by Hrr25p, a yeast homolog of casein kinase I.](http://www.ncbi.nlm.nih.gov/pubmed/14597664) *Genes Dev.* 2003; **17:** 2698-708.

Sopko R, Huang D, Preston N, Chua G, Papp B, Kafadar K, Snyder M, Oliver SG, Cyert M, Hughes TR, Boone C, Andrews B. [Mapping pathways and phenotypes by systematic gene overexpression.](http://www.ncbi.nlm.nih.gov/pubmed/16455487) *Mol Cell.* 2006; **21:** 319-30.

Williams KE, Cyert MS. [The eukaryotic response regulator Skn7p regulates calcineurin signaling through stabilization of Crz1p.](http://www.ncbi.nlm.nih.gov/pubmed/11432834) *EMBO J.* 2001; **20:** 3473-83.

Kafadar KA, Cyert MS. [Integration of stress responses: modulation of calcineurin signaling in Saccharomyces cerevisiae by protein kinase A.](http://www.ncbi.nlm.nih.gov/pubmed/15470242) *Eukaryot Cell.* 2004; **3:** 1147-53.

Polizotto RS, Cyert MS. [Calcineurin-dependent nuclear import of the transcription factor Crz1p requires Nmd5p.](http://www.ncbi.nlm.nih.gov/pubmed/11535618) *J Cell Biol.* 2001; **154:** 951-60.

Mulet JM, Martin DE, Loewith R, Hall MN. [Mutual antagonism of target of rapamycin and calcineurin signaling.](http://www.ncbi.nlm.nih.gov/pubmed/16959779) *J Biol Chem.* 2006; **281:** 33000-7.

Rusnak F, Mertz P. [Calcineurin: form and function.](http://www.ncbi.nlm.nih.gov/pubmed/11015619) *Physiol Rev.* 2000; **80:** 1483-521.

Cunningham KW, Fink GR. [Calcineurin inhibits VCX1-dependent H+/Ca2+ exchange and induces Ca2+ ATPases in Saccharomyces cerevisiae.](http://www.ncbi.nlm.nih.gov/pubmed/8628289) *Mol Cell Biol.* 1996; **16:** 2226-37.

Mendoza I, Rubio F, Rodriguez-Navarro A, Pardo JM. [The protein phosphatase calcineurin is essential for NaCl tolerance of Saccharomyces cerevisiae.](http://www.ncbi.nlm.nih.gov/pubmed/8132612) *J Biol Chem.* 1994; **269:** 8792-6.

Yoshimoto H, Saltsman K, Gasch AP, Li HX, Ogawa N, Botstein D, Brown PO, Cyert MS. [Genome-wide analysis of gene expression regulated by the calcineurin/Crz1p signaling pathway in Saccharomyces cerevisiae.](http://www.ncbi.nlm.nih.gov/pubmed/12058033) *J Biol Chem.* 2002;**277:** 31079-88.

Cyert MS. [Calcineurin signaling in Saccharomyces cerevisiae: how yeast go crazy in response to stress.](http://www.ncbi.nlm.nih.gov/pubmed/14623300) *Biochem Biophys Res Commun.* 2003; **311:** 1143-50.

Ruiz A, Serrano R, Ariño J. [Direct regulation of genes involved in glucose utilization by the calcium/calcineurin pathway.](http://www.ncbi.nlm.nih.gov/pubmed/18362157) *J Biol Chem.* 2008; **283:** 13923-33.

Jouandot D 2nd, Roy A, Kim JH. [Functional dissection of the glucose signaling pathways that regulate the yeast glucose transporter gene (HXT) repressor Rgt1.](http://www.ncbi.nlm.nih.gov/pubmed/21748783) *J Cell Biochem.* 2011; **112:** 3268-75.

Gadura N, Robinson LC, Michels CA. [Glc7-Reg1 phosphatase signals to Yck1,2 casein kinase 1 to regulate transport activity and glucose-induced inactivation of Saccharomyces maltose permease.](http://www.ncbi.nlm.nih.gov/pubmed/16361229) *Genetics.* 2006; **172:** 1427-39.

Marchal C, Haguenauer-Tsapis R, Urban-Grimal D. [Casein kinase I-dependent phosphorylation within a PEST sequence and ubiquitination at nearby lysines signal endocytosis of yeast uracil permease.](http://www.ncbi.nlm.nih.gov/pubmed/10811641) *J Biol Chem.* 2000; **275:** 23608-14.

Gualtieri T, Ragni E, Mizzi L, Fascio U, Popolo L. [The cell wall sensor Wsc1p is involved in reorganization of actin cytoskeleton in response to hypo-osmotic shock in Saccharomyces cerevisiae.](http://www.ncbi.nlm.nih.gov/pubmed/15484288) *Yeast.* 2004; **21:** 1107-20.

Jung US, Levin DE. [Genome-wide analysis of gene expression regulated by the yeast cell wall integrity signalling pathway.](http://www.ncbi.nlm.nih.gov/pubmed/10594829)*Mol Microbiol.* 1999; **34:** 1049-57.

Zhan XL, Deschenes RJ, Guan KL. [Differential regulation of FUS3 MAP kinase by tyrosine-specific phosphatases PTP2/PTP3 and dual-specificity phosphatase MSG5 in Saccharomyces cerevisiae.](http://www.ncbi.nlm.nih.gov/pubmed/9224718) *Genes Dev.* 1997; **11:** 1690-702.

Carmody SR, Tran EJ, Apponi LH, Corbett AH, Wente SR. [The mitogen-activated protein kinase Slt2 regulates nuclear retention of non-heat shock mRNAs during heat shock-induced stress.](http://www.ncbi.nlm.nih.gov/pubmed/20823268) *Mol Cell Biol.* 2010; **30:** 5168-79.

Madden K, Sheu YJ, Baetz K, Andrews B, Snyder M. [SBF cell cycle regulator as a target of the yeast PKC-MAP kinase pathway.](http://www.ncbi.nlm.nih.gov/pubmed/9065400) *Science.* 1997; **275:** 1781-4.

Baetz K, Moffat J, Haynes J, Chang M, Andrews B. [Transcriptional coregulation by the cell integrity mitogen-activated protein kinase Slt2 and the cell cycle regulator Swi4.](http://www.ncbi.nlm.nih.gov/pubmed/11533240) *Mol Cell Biol.* 2001; **21:** 6515-28.

Kim KY, Levin DE. [Transcriptional reporters for genes activated by cell wall stress through a non-catalytic mechanism involving Mpk1 and SBF.](http://www.ncbi.nlm.nih.gov/pubmed/20641022) *Yeast.* 2010; **27:** 541-8.

Koch C, Schleiffer A, Ammerer G, Nasmyth K. [Switching transcription on and off during the yeast cell cycle: Cln/Cdc28 kinases activate bound transcription factor SBF (Swi4/Swi6) at start, whereas Clb/Cdc28 kinases displace it from the promoter in G2.](http://www.ncbi.nlm.nih.gov/pubmed/8566747) *Genes Dev.* 1996; **10:** 129-41.

Harreman MT, Kline TM, Milford HG, Harben MB, Hodel AE, Corbett AH. [Regulation of nuclear import by phosphorylation adjacent to nuclear localization signals.](http://www.ncbi.nlm.nih.gov/pubmed/14998990) *J Biol Chem.* 2004; **279:** 20613-21.

Hsieh YY, Hung PH, Leu JY. [Hsp90 regulates nongenetic variation in response to environmental stress.](http://www.ncbi.nlm.nih.gov/pubmed/23434373) *Mol Cell.* 2013; **50:**82-92.

Abbas-Terki T, Donzé O, Picard D. [The molecular chaperone Cdc37 is required for Ste11 function and pheromone-induced cell cycle arrest.](http://www.ncbi.nlm.nih.gov/pubmed/10664467) *FEBS Lett.* 2000; **467:** 111-6.

Donzé O, Picard D. [Hsp90 binds and regulates Gcn2, the ligand-inducible kinase of the alpha subunit of eukaryotic translation initiation factor 2 [corrected].](http://www.ncbi.nlm.nih.gov/pubmed/10567567) *Mol Cell Biol.* 1999; **19:** 8422-32.

Reinders A, Bürckert N, Boller T, Wiemken A, De Virgilio C. [Saccharomyces cerevisiae cAMP-dependent protein kinase controls entry into stationary phase through the Rim15p protein kinase.](http://www.ncbi.nlm.nih.gov/pubmed/9744870) *Genes Dev.* 1998; **12:** 2943-55.

Wanke V, Pedruzzi I, Cameroni E, Dubouloz F, De Virgilio C. [Regulation of G0 entry by the Pho80-Pho85 cyclin-CDK complex.](http://www.ncbi.nlm.nih.gov/pubmed/16308562) *EMBO J.* 2005; **24:** 4271-8.

Kwast KE, Burke PV, Poyton RO. [Oxygen sensing and the transcriptional regulation of oxygen-responsive genes in yeast.](http://www.ncbi.nlm.nih.gov/pubmed/9510529)*J Exp Biol.* 1998; **201:** 1177-95.

Xie Y, Varshavsky A. [RPN4 is a ligand, substrate, and transcriptional regulator of the 26S proteasome: a negative feedback circuit.](http://www.ncbi.nlm.nih.gov/pubmed/11248031) *Proc Natl Acad Sci U S A.* 2001; **98:** 3056-61.

Zhang L, Bermingham-McDonogh O, Turcotte B, Guarente L. [Antibody-promoted dimerization bypasses the regulation of DNA binding by the heme domain of the yeast transcriptional activator HAP1.](http://www.ncbi.nlm.nih.gov/pubmed/8464899) *Proc Natl Acad Sci U S A.* 1993; **90:**2851-5.

Hon T, Hach A, Tamalis D, Zhu Y, Zhang L. [The yeast heme-responsive transcriptional activator Hap1 is a preexisting dimer in the absence of heme.](http://www.ncbi.nlm.nih.gov/pubmed/10428861) *J Biol Chem.* 1999; **274:** 22770-4.

Zhang L, Guarente L. [HAP1 is nuclear but is bound to a cellular factor in the absence of heme.](http://www.ncbi.nlm.nih.gov/pubmed/8182072) *J Biol Chem.* 1994; **269:**14643-7.

Beck T, Hall MN. [The TOR signalling pathway controls nuclear localization of nutrient-regulated transcription factors.](http://www.ncbi.nlm.nih.gov/pubmed/10604478)*Nature.* 1999; **402:** 689-92.

Evangelista M, Pruyne D, Amberg DC, Boone C, Bretscher A. [Formins direct Arp2/3-independent actin filament assembly to polarize cell growth in yeast.](http://www.ncbi.nlm.nih.gov/pubmed/11875440) *Nat Cell Biol.* 2002; **4:** 260-9.

Klis FM, Mol P, Hellingwerf K, Brul S. [Dynamics of cell wall structure in Saccharomyces cerevisiae.](http://www.ncbi.nlm.nih.gov/pubmed/12165426) *FEMS Microbiol Rev.*2002; **26:** 239-56.

Morano KA, Thiele DJ. [The Sch9 protein kinase regulates Hsp90 chaperone complex signal transduction activity in vivo.](http://www.ncbi.nlm.nih.gov/pubmed/10545107)*EMBO J.* 1999; **18:** 5953-62.

Hector RE, Nykamp KR, Dheur S, Anderson JT, Non PJ, Urbinati CR, Wilson SM, Minvielle-Sebastia L, Swanson MS. [Dual requirement for yeast hnRNP Nab2p in mRNA poly(A) tail length control and nuclear export.](http://www.ncbi.nlm.nih.gov/pubmed/11927564) *EMBO J.* 2002; **21:** 1800-10.

Platara M, Ruiz A, Serrano R, Palomino A, Moreno F, Ariño J. [The transcriptional response of the yeast Na(+)-ATPase ENA1 gene to alkaline stress involves three main signaling pathways.](http://www.ncbi.nlm.nih.gov/pubmed/17023428) *J Biol Chem.* 2006; **281:** 36632-42.

Mascarenhas C, Edwards-Ingram LC, Zeef L, Shenton D, Ashe MP, Grant CM. [Gcn4 is required for the response to peroxide stress in the yeast Saccharomyces cerevisiae.](http://www.ncbi.nlm.nih.gov/pubmed/18417611) *Mol Biol Cell.* 2008; **19:** 2995-3007.

Kimpe M, Voordeckers K, Thevelein JM, Van Zeebroeck G. [Pkh1 interacts with and phosphorylates components of the yeast Gcn2/eIF2α system.](http://www.ncbi.nlm.nih.gov/pubmed/22326914) *Biochem Biophys Res Commun.* 2012; **419:** 89-94.

Pavitt GD, Ramaiah KV, Kimball SR, Hinnebusch AG. [eIF2 independently binds two distinct eIF2B subcomplexes that catalyze and regulate guanine-nucleotide exchange.](http://www.ncbi.nlm.nih.gov/pubmed/9472020) *Genes Dev.* 1998; **12:** 514-26.

Cherkasova V, Qiu H, Hinnebusch AG. [Snf1 promotes phosphorylation of the alpha subunit of eukaryotic translation initiation factor 2 by activating Gcn2 and inhibiting phosphatases Glc7 and Sit4.](http://www.ncbi.nlm.nih.gov/pubmed/20404097) *Mol Cell Biol.* 2010; **30:** 2862-73.

Broach JR. [Nutritional control of growth and development in yeast.](http://www.ncbi.nlm.nih.gov/pubmed/22964838) *Genetics.* 2012; **192:** 73-105.

Netz DJ, Pierik AJ, Stümpfig M, Mühlenhoff U, Lill R. [The Cfd1-Nbp35 complex acts as a scaffold for iron-sulfur protein assembly in the yeast cytosol.](http://www.ncbi.nlm.nih.gov/pubmed/17401378) *Nat Chem Biol.* 2007; **3:** 278-86.

Xiao Z, Loughlin F, George GN, Howlett GJ, Wedd AG. [C-terminal domain of the membrane copper transporter Ctr1 from Saccharomyces cerevisiae binds four Cu(I) ions as a cuprous-thiolate polynuclear cluster: sub-femtomolar Cu(I) affinity of three proteins involved in copper trafficking.](http://www.ncbi.nlm.nih.gov/pubmed/15012137) *J Am Chem Soc.* 2004; **126:** 3081-90.

Lin SJ, Pufahl RA, Dancis A, O'Halloran TV, Culotta VC. [A role for the Saccharomyces cerevisiae ATX1 gene in copper trafficking and iron transport.](http://www.ncbi.nlm.nih.gov/pubmed/9083054) *J Biol Chem.* 1997; **272:** 9215-20.

Iida H, Nakamura H, Ono T, Okumura MS, Anraku Y. [MID1, a novel Saccharomyces cerevisiae gene encoding a plasma membrane protein, is required for Ca2+ influx and mating.](http://www.ncbi.nlm.nih.gov/pubmed/7526155) *Mol Cell Biol.* 1994; **14:** 8259-71.

Philpott CC, Protchenko O, Kim YW, Boretsky Y, Shakoury-Elizeh M. [The response to iron deprivation in Saccharomyces cerevisiae: expression of siderophore-based systems of iron uptake.](http://www.ncbi.nlm.nih.gov/pubmed/12196168) *Biochem Soc Trans.* 2002; **30:** 698-702.

Labbé S, Zhu Z, Thiele DJ. [Copper-specific transcriptional repression of yeast genes encoding critical components in the copper transport pathway.](http://www.ncbi.nlm.nih.gov/pubmed/9188496) *J Biol Chem.* 1997; **272:** 15951-8.

Saito H, Posas F. [Response to hyperosmotic stress.](http://www.ncbi.nlm.nih.gov/pubmed/23028184) *Genetics.* 2012; **192:** 289-318.

Doi K, Gartner A, Ammerer G, Errede B, Shinkawa H, Sugimoto K, Matsumoto K. [MSG5, a novel protein phosphatase promotes adaptation to pheromone response in S. cerevisiae.](http://www.ncbi.nlm.nih.gov/pubmed/8306972) *EMBO J.* 1994; **13:** 61-70.

Blackwell E, Kim HJ, Stone DE. [The pheromone-induced nuclear accumulation of the Fus3 MAPK in yeast depends on its phosphorylation state and on Dig1 and Dig2.](http://www.ncbi.nlm.nih.gov/pubmed/17963515) *BMC Cell Biol.* 2007; **8:** 44.

Serrano R, Martín H, Casamayor A, Ariño J. [Signaling alkaline pH stress in the yeast Saccharomyces cerevisiae through the Wsc1 cell surface sensor and the Slt2 MAPK pathway.](http://www.ncbi.nlm.nih.gov/pubmed/17088254) *J Biol Chem.* 2006; **281:** 39785-95.

Cid VJ, Cenamor R, Sánchez M, Nombela C. [A mutation in the Rho1-GAP-encoding gene BEM2 of Saccharomyces cerevisiae affects morphogenesis and cell wall functionality.](http://www.ncbi.nlm.nih.gov/pubmed/9467898) *Microbiology.* 1998; **144 ( Pt 1):** 25-36.

Roumanie O, Weinachter C, Larrieu I, Crouzet M, Doignon F. [Functional characterization of the Bag7, Lrg1 and Rgd2 RhoGAP proteins from Saccharomyces cerevisiae.](http://www.ncbi.nlm.nih.gov/pubmed/11591390) *FEBS Lett.* 2001; **506:** 149-56.

Schmidt A, Bickle M, Beck T, Hall MN. [The yeast phosphatidylinositol kinase homolog TOR2 activates RHO1 and RHO2 via the exchange factor ROM2.](http://www.ncbi.nlm.nih.gov/pubmed/9038344) *Cell.* 1997; **88:** 531-42.

Schmidt A, Schmelzle T, Hall MN. [The RHO1-GAPs SAC7, BEM2 and BAG7 control distinct RHO1 functions in Saccharomyces cerevisiae.](http://www.ncbi.nlm.nih.gov/pubmed/12207708) *Mol Microbiol.* 2002; **45:** 1433-41.

Watanabe D, Abe M, Ohya Y. [Yeast Lrg1p acts as a specialized RhoGAP regulating 1,3-beta-glucan synthesis.](http://www.ncbi.nlm.nih.gov/pubmed/11447600) *Yeast.*2001; **18:** 943-51.

Blackwell E, Halatek IM, Kim HJ, Ellicott AT, Obukhov AA, Stone DE. [Effect of the pheromone-responsive G(alpha) and phosphatase proteins of Saccharomyces cerevisiae on the subcellular localization of the Fus3 mitogen-activated protein kinase.](http://www.ncbi.nlm.nih.gov/pubmed/12556475) *Mol Cell Biol.* 2003; **23:** 1135-50.

Espinoza FH, Ogas J, Herskowitz I, Morgan DO. [Cell cycle control by a complex of the cyclin HCS26 (PCL1) and the kinase PHO85.](http://www.ncbi.nlm.nih.gov/pubmed/7973730) *Science.* 1994; **266:** 1388-91.

Bornaes C, Ignjatovic MW, Schjerling P, Kielland-Brandt MC, Holmberg S. [A regulatory element in the CHA1 promoter which confers inducibility by serine and threonine on Saccharomyces cerevisiae genes.](http://www.ncbi.nlm.nih.gov/pubmed/8246977) *Mol Cell Biol.* 1993; **13:** 7604-11.

Choudhary V, Schneiter R. [Pathogen-Related Yeast (PRY) proteins and members of the CAP superfamily are secreted sterol-binding proteins.](http://www.ncbi.nlm.nih.gov/pubmed/23027975) *Proc Natl Acad Sci U S A.* 2012; **109:** 16882-7.

Kuchin S, Vyas VK, Carlson M. [Snf1 protein kinase and the repressors Nrg1 and Nrg2 regulate FLO11, haploid invasive growth, and diploid pseudohyphal differentiation.](http://www.ncbi.nlm.nih.gov/pubmed/12024013) *Mol Cell Biol.* 2002; **22:** 3994-4000.

Vyas VK, Kuchin S, Carlson M. [Interaction of the repressors Nrg1 and Nrg2 with the Snf1 protein kinase in Saccharomyces cerevisiae.](http://www.ncbi.nlm.nih.gov/pubmed/11404322) *Genetics.* 2001; **158:** 563-72.

Lamb TM, Xu W, Diamond A, Mitchell AP. [Alkaline response genes of Saccharomyces cerevisiae and their relationship to the RIM101 pathway.](http://www.ncbi.nlm.nih.gov/pubmed/11050096) *J Biol Chem.* 2001; **276:** 1850-6.

Guo B, Styles CA, Feng Q, Fink GR. [A Saccharomyces gene family involved in invasive growth, cell-cell adhesion, and mating.](http://www.ncbi.nlm.nih.gov/pubmed/11027318) *Proc Natl Acad Sci U S A.* 2000; **97:** 12158-63.

Park SH, Koh SS, Chun JH, Hwang HJ, Kang HS. [Nrg1 is a transcriptional repressor for glucose repression of STA1 gene expression in Saccharomyces cerevisiae.](http://www.ncbi.nlm.nih.gov/pubmed/10022891) *Mol Cell Biol.* 1999; **19:** 2044-50.

Zhou H, Winston F. [NRG1 is required for glucose repression of the SUC2 and GAL genes of Saccharomyces cerevisiae.](http://www.ncbi.nlm.nih.gov/pubmed/11281938)*BMC Genet.* 2001; **2:** 5.

Berkey CD, Vyas VK, Carlson M. [Nrg1 and nrg2 transcriptional repressors are differently regulated in response to carbon source.](http://www.ncbi.nlm.nih.gov/pubmed/15075261) *Eukaryot Cell.* 2004; **3:** 311-7.

Varanasi US, Klis M, Mikesell PB, Trumbly RJ. [The Cyc8 (Ssn6)-Tup1 corepressor complex is composed of one Cyc8 and four Tup1 subunits.](http://www.ncbi.nlm.nih.gov/pubmed/8943325) *Mol Cell Biol.* 1996; **16:** 6707-14.

Obara K, Yamamoto H, Kihara A. [Membrane protein Rim21 plays a central role in sensing ambient pH in Saccharomyces cerevisiae.](http://www.ncbi.nlm.nih.gov/pubmed/23019326) *J Biol Chem.* 2012; **287:** 38473-81.

Lamb TM, Mitchell AP. [The transcription factor Rim101p governs ion tolerance and cell differentiation by direct repression of the regulatory genes NRG1 and SMP1 in Saccharomyces cerevisiae.](http://www.ncbi.nlm.nih.gov/pubmed/12509465) *Mol Cell Biol.* 2003; **23:** 677-86.

Maeda T. [The signaling mechanism of ambient pH sensing and adaptation in yeast and fungi.](http://www.ncbi.nlm.nih.gov/pubmed/22360598) *FEBS J.* 2012; **279:** 1407-13.

Babst M, Katzmann DJ, Estepa-Sabal EJ, Meerloo T, Emr SD. [Escrt-III: an endosome-associated heterooligomeric protein complex required for mvb sorting.](http://www.ncbi.nlm.nih.gov/pubmed/12194857) *Dev Cell.* 2002; **3:** 271-82.

Herrador A, Herranz S, Lara D, Vincent O. [Recruitment of the ESCRT machinery to a putative seven-transmembrane-domain receptor is mediated by an arrestin-related protein.](http://www.ncbi.nlm.nih.gov/pubmed/20028738) *Mol Cell Biol.* 2010; **30:** 897-907.

Görner W, Durchschlag E, Wolf J, Brown EL, Ammerer G, Ruis H, Schüller C. [Acute glucose starvation activates the nuclear localization signal of a stress-specific yeast transcription factor.](http://www.ncbi.nlm.nih.gov/pubmed/11782433) *EMBO J.* 2002; **21:** 135-44.

Görner W, Durchschlag E, Martinez-Pastor MT, Estruch F, Ammerer G, Hamilton B, Ruis H, Schüller C. [Nuclear localization of the C2H2 zinc finger protein Msn2p is regulated by stress and protein kinase A activity.](http://www.ncbi.nlm.nih.gov/pubmed/9472026) *Genes Dev.*1998; **12:** 586-97.

Livas D, Almering MJ, Daran JM, Pronk JT, Gancedo JM. [Transcriptional responses to glucose in Saccharomyces cerevisiae strains lacking a functional protein kinase A.](http://www.ncbi.nlm.nih.gov/pubmed/21827659) *BMC Genomics.* 2011; **12:** 405.

Vendrell A, Martínez-Pastor M, González-Novo A, Pascual-Ahuir A, Sinclair DA, Proft M, Posas F. [Sir2 histone deacetylase prevents programmed cell death caused by sustained activation of the Hog1 stress-activated protein kinase.](http://www.ncbi.nlm.nih.gov/pubmed/21836634) *EMBO Rep.* 2011; **12:** 1062-8.

Bang SY, Kim JH, Lee PY, Chi SW, Cho S, Yi GS, Myung PK, Park BC, Bae KH, Park SG. [Candidate target genes for the Saccharomyces cerevisiae transcription factor, Yap2.](http://www.ncbi.nlm.nih.gov/pubmed/23334931) *Folia Microbiol (Praha).* 2013; **58:** 403-8.

Bilsland E, Molin C, Swaminathan S, Ramne A, Sunnerhagen P. [Rck1 and Rck2 MAPKAP kinases and the HOG pathway are required for oxidative stress resistance.](http://www.ncbi.nlm.nih.gov/pubmed/15341652) *Mol Microbiol.* 2004; **53:** 1743-56.

Bilsland-Marchesan E, Ariño J, Saito H, Sunnerhagen P, Posas F. [Rck2 kinase is a substrate for the osmotic stress-activated mitogen-activated protein kinase Hog1.](http://www.ncbi.nlm.nih.gov/pubmed/10805732) *Mol Cell Biol.* 2000; **20:** 3887-95.

Wang TP, Quintanar L, Severance S, Solomon EI, Kosman DJ. [Targeted suppression of the ferroxidase and iron trafficking activities of the multicopper oxidase Fet3p from Saccharomyces cerevisiae.](http://www.ncbi.nlm.nih.gov/pubmed/12684851) *J Biol Inorg Chem.* 2003; **8:** 611-20.

Bermingham-McDonogh O, Gralla EB, Valentine JS. [The copper, zinc-superoxide dismutase gene of Saccharomyces cerevisiae: cloning, sequencing, and biological activity.](http://www.ncbi.nlm.nih.gov/pubmed/3290902) *Proc Natl Acad Sci U S A.* 1988; **85:** 4789-93.

Harvey SL, Charlet A, Haas W, Gygi SP, Kellogg DR. [Cdk1-dependent regulation of the mitotic inhibitor Wee1.](http://www.ncbi.nlm.nih.gov/pubmed/16096060) *Cell.* 2005;**122:** 407-20.

Miyakawa T, Mizunuma M. [Physiological roles of calcineurin in Saccharomyces cerevisiae with special emphasis on its roles in G2/M cell-cycle regulation.](http://www.ncbi.nlm.nih.gov/pubmed/17341827) *Biosci Biotechnol Biochem.* 2007; **71:** 633-45.

Wittenberg C, Sugimoto K, Reed SI. [G1-specific cyclins of S. cerevisiae: cell cycle periodicity, regulation by mating pheromone, and association with the p34CDC28 protein kinase.](http://www.ncbi.nlm.nih.gov/pubmed/2142620) *Cell.* 1990; **62:** 225-37.

Nasmyth K. [At the heart of the budding yeast cell cycle.](http://www.ncbi.nlm.nih.gov/pubmed/8909137) *Trends Genet.* 1996; **12:** 405-12.

Rep M, Krantz M, Thevelein JM, Hohmann S. [The transcriptional response of Saccharomyces cerevisiae to osmotic shock. Hot1p and Msn2p/Msn4p are required for the induction of subsets of high osmolarity glycerol pathway-dependent genes.](http://www.ncbi.nlm.nih.gov/pubmed/10722658) *J Biol Chem.* 2000; **275:** 8290-300.

López-Martínez G, Rodríguez-Porrata B, Margalef-Català M, Cordero-Otero R. [The STF2p hydrophilin from Saccharomyces cerevisiae is required for dehydration stress tolerance.](http://www.ncbi.nlm.nih.gov/pubmed/22442684) *PLoS One.* 2012; **7:** e33324.

Carman GM, Han GS. [Regulation of phospholipid synthesis in Saccharomyces cerevisiae by zinc depletion.](http://www.ncbi.nlm.nih.gov/pubmed/16807089) *Biochim Biophys Acta.* 2007; **1771:** 322-30.

Petelenz-Kurdziel E, Kuehn C, Nordlander B, Klein D, Hong KK, Jacobson T, Dahl P, Schaber J, Nielsen J, Hohmann S, Klipp E. [Quantitative analysis of glycerol accumulation, glycolysis and growth under hyper osmotic stress.](http://www.ncbi.nlm.nih.gov/pubmed/23762021) *PLoS Comput Biol.* 2013; **9:** e1003084.

Moser MJ, Geiser JR, Davis TN. [Ca2+-calmodulin promotes survival of pheromone-induced growth arrest by activation of calcineurin and Ca2+-calmodulin-dependent protein kinase.](http://www.ncbi.nlm.nih.gov/pubmed/8756641) *Mol Cell Biol.* 1996; **16:** 4824-31.

Yuzyuk T, Foehr M, Amberg DC. [The MEK kinase Ssk2p promotes actin cytoskeleton recovery after osmotic stress.](http://www.ncbi.nlm.nih.gov/pubmed/12181352) *Mol Biol Cell.* 2002; **13:** 2869-80.

Rolland F, De Winde JH, Lemaire K, Boles E, Thevelein JM, Winderickx J. [Glucose-induced cAMP signalling in yeast requires both a G-protein coupled receptor system for extracellular glucose detection and a separable hexose kinase-dependent sensing process.](http://www.ncbi.nlm.nih.gov/pubmed/11069660) *Mol Microbiol.* 2000; **38:** 348-58.

Gancedo JM. [The early steps of glucose signalling in yeast.](http://www.ncbi.nlm.nih.gov/pubmed/18559076) *FEMS Microbiol Rev.* 2008; **32:** 673-704.

Ansari K, Martin S, Farkasovsky M, Ehbrecht IM, Küntzel H. [Phospholipase C binds to the receptor-like GPR1 protein and controls pseudohyphal differentiation in Saccharomyces cerevisiae.](http://www.ncbi.nlm.nih.gov/pubmed/10514491) *J Biol Chem.* 1999; **274:** 30052-8.

Schuhmann R, Lehmann WD. [[The relationship between placental morphology and biochemical parameters in EPH-gestosis (toxemia) and diabetes mellitus. Dehydroepiandrosterone-test, in vitro conversion-rate of 4-14C-dehydroepiandrosterone to estrogens, maternal 24 h-urinary estrogen excertion (author's transl)].](http://www.ncbi.nlm.nih.gov/pubmed/4270275) *Arch Gynakol.* 1973;**215:** 72-84.

Capaldi AP, Kaplan T, Liu Y, Habib N, Regev A, Friedman N, O'Shea EK. [Structure and function of a transcriptional network activated by the MAPK Hog1.](http://www.ncbi.nlm.nih.gov/pubmed/18931682) *Nat Genet.* 2008; **40:** 1300-6.

Gallo CM, Smith DL Jr, Smith JS. [Nicotinamide clearance by Pnc1 directly regulates Sir2-mediated silencing and longevity.](http://www.ncbi.nlm.nih.gov/pubmed/14729974) *Mol Cell Biol.* 2004; **24:** 1301-12.

Hohmann S. [Osmotic stress signaling and osmoadaptation in yeasts.](http://www.ncbi.nlm.nih.gov/pubmed/12040128) *Microbiol Mol Biol Rev.* 2002; **66:** 300-72.

de Nadal E, Casadomé L, Posas F. [Targeting the MEF2-like transcription factor Smp1 by the stress-activated Hog1 mitogen-activated protein kinase.](http://www.ncbi.nlm.nih.gov/pubmed/12482976) *Mol Cell Biol.* 2003; **23:** 229-37.

Kandror O, Bretschneider N, Kreydin E, Cavalieri D, Goldberg AL. [Yeast adapt to near-freezing temperatures by STRE/Msn2,4-dependent induction of trehalose synthesis and certain molecular chaperones.](http://www.ncbi.nlm.nih.gov/pubmed/15053871) *Mol Cell.* 2004; **13:** 771-81.

Márquez JA, Pascual-Ahuir A, Proft M, Serrano R. [The Ssn6-Tup1 repressor complex of Saccharomyces cerevisiae is involved in the osmotic induction of HOG-dependent and -independent genes.](http://www.ncbi.nlm.nih.gov/pubmed/9564037) *EMBO J.* 1998; **17:** 2543-53.

Lin H, Nguyen P, Vancura A. [Phospholipase C interacts with Sgd1p and is required for expression of GPD1 and osmoresistance in Saccharomyces cerevisiae.](http://www.ncbi.nlm.nih.gov/pubmed/12073033) *Mol Genet Genomics.* 2002; **267:** 313-20.

Rep M, Reiser V, Gartner U, Thevelein JM, Hohmann S, Ammerer G, Ruis H. [Osmotic stress-induced gene expression in Saccharomyces cerevisiae requires Msn1p and the novel nuclear factor Hot1p.](http://www.ncbi.nlm.nih.gov/pubmed/10409737) *Mol Cell Biol.* 1999; **19:** 5474-85.

Martínez-Pastor MT, Marchler G, Schüller C, Marchler-Bauer A, Ruis H, Estruch F. [The Saccharomyces cerevisiae zinc finger proteins Msn2p and Msn4p are required for transcriptional induction through the stress response element (STRE).](http://www.ncbi.nlm.nih.gov/pubmed/8641288) *EMBO J.* 1996; **15:** 2227-35.

Schmitt AP, McEntee K. [Msn2p, a zinc finger DNA-binding protein, is the transcriptional activator of the multistress response in Saccharomyces cerevisiae.](http://www.ncbi.nlm.nih.gov/pubmed/8650168) *Proc Natl Acad Sci U S A.* 1996; **93:** 5777-82.

Akhtar N, Påhlman AK, Larsson K, Corbett AH, Adler L. [SGD1 encodes an essential nuclear protein of Saccharomyces cerevisiae that affects expression of the GPD1 gene for glycerol 3-phosphate dehydrogenase.](http://www.ncbi.nlm.nih.gov/pubmed/11042259) *FEBS Lett.* 2000; **483:**87-92.

Inoue Y, Tsujimoto Y, Kimura A. [Expression of the glyoxalase I gene of Saccharomyces cerevisiae is regulated by high osmolarity glycerol mitogen-activated protein kinase pathway in osmotic stress response.](http://www.ncbi.nlm.nih.gov/pubmed/9446611) *J Biol Chem.* 1998; **273:**2977-83.

Rep M, Albertyn J, Thevelein JM, Prior BA, Hohmann S. [Different signalling pathways contribute to the control of GPD1 gene expression by osmotic stress in Saccharomyces cerevisiae.](http://www.ncbi.nlm.nih.gov/pubmed/10217506) *Microbiology.* 1999; **145 ( Pt 3):** 715-27.

de Nadal E, Posas F. [Multilayered control of gene expression by stress-activated protein kinases.](http://www.ncbi.nlm.nih.gov/pubmed/19942851) *EMBO J.* 2010; **29:** 4-13.

Jacoby T, Flanagan H, Faykin A, Seto AG, Mattison C, Ota I. [Two protein-tyrosine phosphatases inactivate the osmotic stress response pathway in yeast by targeting the mitogen-activated protein kinase, Hog1.](http://www.ncbi.nlm.nih.gov/pubmed/9211927) *J Biol Chem.* 1997; **272:**17749-55.

Dix DR, Bridgham JT, Broderius MA, Byersdorfer CA, Eide DJ. [The FET4 gene encodes the low affinity Fe(II) transport protein of Saccharomyces cerevisiae.](http://www.ncbi.nlm.nih.gov/pubmed/7929320) *J Biol Chem.* 1994; **269:** 26092-9.

Hassett R, Dix DR, Eide DJ, Kosman DJ. [The Fe(II) permease Fet4p functions as a low affinity copper transporter and supports normal copper trafficking in Saccharomyces cerevisiae.](http://www.ncbi.nlm.nih.gov/pubmed/11023834) *Biochem J.* 2000; **351 Pt 2:** 477-84.

Urbanowski JL, Piper RC. [The iron transporter Fth1p forms a complex with the Fet5 iron oxidase and resides on the vacuolar membrane.](http://www.ncbi.nlm.nih.gov/pubmed/10608875) *J Biol Chem.* 1999; **274:** 38061-70.

Ellis CD, Macdiarmid CW, Eide DJ. [Heteromeric protein complexes mediate zinc transport into the secretory pathway of eukaryotic cells.](http://www.ncbi.nlm.nih.gov/pubmed/15961382) *J Biol Chem.* 2005; **280:** 28811-8.

Zheng Y, Hart MJ, Shinjo K, Evans T, Bender A, Cerione RA. [Biochemical comparisons of the Saccharomyces cerevisiae Bem2 and Bem3 proteins. Delineation of a limit Cdc42 GTPase-activating protein domain.](http://www.ncbi.nlm.nih.gov/pubmed/8227021) *J Biol Chem.* 1993; **268:**24629-34.

Smith GR, Givan SA, Cullen P, Sprague GF Jr. [GTPase-activating proteins for Cdc42.](http://www.ncbi.nlm.nih.gov/pubmed/12455995) *Eukaryot Cell.* 2002; **1:** 469-80.

Stevenson BJ, Ferguson B, De Virgilio C, Bi E, Pringle JR, Ammerer G, Sprague GF Jr. [Mutation of RGA1, which encodes a putative GTPase-activating protein for the polarity-establishment protein Cdc42p, activates the pheromone-response pathway in the yeast Saccharomyces cerevisiae.](http://www.ncbi.nlm.nih.gov/pubmed/7498791) *Genes Dev.* 1995; **9:** 2949-63.

Benton BK, Tinkelenberg A, Gonzalez I, Cross FR. [Cla4p, a Saccharomyces cerevisiae Cdc42p-activated kinase involved in cytokinesis, is activated at mitosis.](http://www.ncbi.nlm.nih.gov/pubmed/9271384) *Mol Cell Biol.* 1997; **17:** 5067-76.

Peter M, Neiman AM, Park HO, van Lohuizen M, Herskowitz I. [Functional analysis of the interaction between the small GTP binding protein Cdc42 and the Ste20 protein kinase in yeast.](http://www.ncbi.nlm.nih.gov/pubmed/9003780) *EMBO J.* 1996; **15:** 7046-59.

Balk J, Aguilar Netz DJ, Tepper K, Pierik AJ, Lill R. [The essential WD40 protein Cia1 is involved in a late step of cytosolic and nuclear iron-sulfur protein assembly.](http://www.ncbi.nlm.nih.gov/pubmed/16314508) *Mol Cell Biol.* 2005; **25:** 10833-41.

Li H, Mapolelo DT, Dingra NN, Naik SG, Lees NS, Hoffman BM, Riggs-Gelasco PJ, Huynh BH, Johnson MK, Outten CE.[The yeast iron regulatory proteins Grx3/4 and Fra2 form heterodimeric complexes containing a [2Fe-2S] cluster with cysteinyl and histidyl ligation.](http://www.ncbi.nlm.nih.gov/pubmed/19715344) *Biochemistry.* 2009; **48:** 9569-81.

Glerum DM, Shtanko A, Tzagoloff A. [SCO1 and SCO2 act as high copy suppressors of a mitochondrial copper recruitment defect in Saccharomyces cerevisiae.](http://www.ncbi.nlm.nih.gov/pubmed/8702795) *J Biol Chem.* 1996; **271:** 20531-5.

Beers J, Glerum DM, Tzagoloff A. [Purification, characterization, and localization of yeast Cox17p, a mitochondrial copper shuttle.](http://www.ncbi.nlm.nih.gov/pubmed/9407107) *J Biol Chem.* 1997; **272:** 33191-6.

Horng YC, Cobine PA, Maxfield AB, Carr HS, Winge DR. [Specific copper transfer from the Cox17 metallochaperone to both Sco1 and Cox11 in the assembly of yeast cytochrome C oxidase.](http://www.ncbi.nlm.nih.gov/pubmed/15199057) *J Biol Chem.* 2004; **279:** 35334-40.

Luk E, Yang M, Jensen LT, Bourbonnais Y, Culotta VC. [Manganese activation of superoxide dismutase 2 in the mitochondria of Saccharomyces cerevisiae.](http://www.ncbi.nlm.nih.gov/pubmed/15851472) *J Biol Chem.* 2005; **280:** 22715-20.

Ravindranath SD, Fridovich I. [Isolation and characterization of a manganese-containing superoxide dismutase from yeast.](http://www.ncbi.nlm.nih.gov/pubmed/238997)*J Biol Chem.* 1975; **250:** 6107-12.

Culotta VC, Yang M, Hall MD. [Manganese transport and trafficking: lessons learned from Saccharomyces cerevisiae.](http://www.ncbi.nlm.nih.gov/pubmed/16002642)*Eukaryot Cell.* 2005; **4:** 1159-65.

Portnoy ME, Jensen LT, Culotta VC. [The distinct methods by which manganese and iron regulate the Nramp transporters in yeast.](http://www.ncbi.nlm.nih.gov/pubmed/11829747) *Biochem J.* 2002; **362:** 119-24.

Sullivan JA, Lewis MJ, Nikko E, Pelham HR. [Multiple interactions drive adaptor-mediated recruitment of the ubiquitin ligase rsp5 to membrane proteins in vivo and in vitro.](http://www.ncbi.nlm.nih.gov/pubmed/17429078) *Mol Biol Cell.* 2007; **18:** 2429-40.

Liu XF, Culotta VC. [Post-translation control of Nramp metal transport in yeast. Role of metal ions and the BSD2 gene.](http://www.ncbi.nlm.nih.gov/pubmed/9988727) *J Biol Chem.* 1999; **274:** 4863-8.

Stimpson HE, Lewis MJ, Pelham HR. [Transferrin receptor-like proteins control the degradation of a yeast metal transporter.](http://www.ncbi.nlm.nih.gov/pubmed/16456538) *EMBO J.* 2006; **25:** 662-72.

Heredia J, Crooks M, Zhu Z. [Phosphorylation and Cu+ coordination-dependent DNA binding of the transcription factor Mac1p in the regulation of copper transport.](http://www.ncbi.nlm.nih.gov/pubmed/11134042) *J Biol Chem.* 2001; **276:** 8793-7.

Georgatsou E, Alexandraki D. [Regulated expression of the Saccharomyces cerevisiae Fre1p/Fre2p Fe/Cu reductase related genes.](http://www.ncbi.nlm.nih.gov/pubmed/10341420) *Yeast.* 1999; **15:** 573-84.

Mizunuma M, Hirata D, Miyaoka R, Miyakawa T. [GSK-3 kinase Mck1 and calcineurin coordinately mediate Hsl1 down-regulation by Ca2+ in budding yeast.](http://www.ncbi.nlm.nih.gov/pubmed/11230131) *EMBO J.* 2001; **20:** 1074-85.

Mapes J, Ota IM. [Nbp2 targets the Ptc1-type 2C Ser/Thr phosphatase to the HOG MAPK pathway.](http://www.ncbi.nlm.nih.gov/pubmed/14685261) *EMBO J.* 2004; **23:**302-11.

Maeda T, Takekawa M, Saito H. [Activation of yeast PBS2 MAPKK by MAPKKKs or by binding of an SH3-containing osmosensor.](http://www.ncbi.nlm.nih.gov/pubmed/7624781) *Science.* 1995; **269:** 554-8.

Lu JM, Deschenes RJ, Fassler JS. [Saccharomyces cerevisiae histidine phosphotransferase Ypd1p shuttles between the nucleus and cytoplasm for SLN1-dependent phosphorylation of Ssk1p and Skn7p.](http://www.ncbi.nlm.nih.gov/pubmed/14665464) *Eukaryot Cell.* 2003; **2:** 1304-14.

Kingsbury TJ, Cunningham KW. [A conserved family of calcineurin regulators.](http://www.ncbi.nlm.nih.gov/pubmed/10887154) *Genes Dev.* 2000; **14:** 1595-604.

MacDiarmid CW, Gaither LA, Eide D. [Zinc transporters that regulate vacuolar zinc storage in Saccharomyces cerevisiae.](http://www.ncbi.nlm.nih.gov/pubmed/10856230)*EMBO J.* 2000; **19:** 2845-55.

Nishimura K, Yasumura K, Igarashi K, Harashima S, Kakinuma Y. [Transcription of some PHO genes in Saccharomyces cerevisiae is regulated by spt7p.](http://www.ncbi.nlm.nih.gov/pubmed/10590460) *Yeast.* 1999; **15:** 1711-7.

Oshima Y. [The phosphatase system in Saccharomyces cerevisiae.](http://www.ncbi.nlm.nih.gov/pubmed/9544531) *Genes Genet Syst.* 1997; **72:** 323-34.

Jensen LT, Ajua-Alemanji M, Culotta VC. [The Saccharomyces cerevisiae high affinity phosphate transporter encoded by PHO84 also functions in manganese homeostasis.](http://www.ncbi.nlm.nih.gov/pubmed/12923174) *J Biol Chem.* 2003; **278:** 42036-40.

Gardarin A, Chédin S, Lagniel G, Aude JC, Godat E, Catty P, Labarre J. [Endoplasmic reticulum is a major target of cadmium toxicity in yeast.](http://www.ncbi.nlm.nih.gov/pubmed/20444096) *Mol Microbiol.* 2010; **76:** 1034-48.

Kren A, Mamnun YM, Bauer BE, Schüller C, Wolfger H, Hatzixanthis K, Mollapour M, Gregori C, Piper P, Kuchler K. [War1p, a novel transcription factor controlling weak acid stress response in yeast.](http://www.ncbi.nlm.nih.gov/pubmed/12588995) *Mol Cell Biol.* 2003; **23:** 1775-85.

Gregori C, Schüller C, Frohner IE, Ammerer G, Kuchler K. [Weak organic acids trigger conformational changes of the yeast transcription factor War1 in vivo to elicit stress adaptation.](http://www.ncbi.nlm.nih.gov/pubmed/18621731) *J Biol Chem.* 2008; **283:** 25752-64.

Piper P, Mahé Y, Thompson S, Pandjaitan R, Holyoak C, Egner R, Mühlbauer M, Coote P, Kuchler K. [The pdr12 ABC transporter is required for the development of weak organic acid resistance in yeast.](http://www.ncbi.nlm.nih.gov/pubmed/9687494) *EMBO J.* 1998; **17:** 4257-65.

Schüller C, Mamnun YM, Mollapour M, Krapf G, Schuster M, Bauer BE, Piper PW, Kuchler K. [Global phenotypic analysis and transcriptional profiling defines the weak acid stress response regulon in Saccharomyces cerevisiae.](http://www.ncbi.nlm.nih.gov/pubmed/14617816) *Mol Biol Cell.*2004; **15:** 706-20.

Gregori C, Bauer B, Schwartz C, Kren A, Schüller C, Kuchler K. [A genetic screen identifies mutations in the yeast WAR1 gene, linking transcription factor phosphorylation to weak-acid stress adaptation.](http://www.ncbi.nlm.nih.gov/pubmed/17509074) *FEBS J.* 2007; **274:** 3094-107.

Holyoak CD, Thompson S, Ortiz Calderon C, Hatzixanthis K, Bauer B, Kuchler K, Piper PW, Coote PJ. [Loss of Cmk1 Ca(2+)-calmodulin-dependent protein kinase in yeast results in constitutive weak organic acid resistance, associated with a post-transcriptional activation of the Pdr12 ATP-binding cassette transporter.](http://www.ncbi.nlm.nih.gov/pubmed/10931353) *Mol Microbiol.* 2000; **37:** 595-605.

Piper P, Calderon CO, Hatzixanthis K, Mollapour M. [Weak acid adaptation: the stress response that confers yeasts with resistance to organic acid food preservatives.](http://www.ncbi.nlm.nih.gov/pubmed/11577142) *Microbiology.* 2001; **147:** 2635-42.

Mollapour M, Fong D, Balakrishnan K, Harris N, Thompson S, Schüller C, Kuchler K, Piper PW. [Screening the yeast deletant mutant collection for hypersensitivity and hyper-resistance to sorbate, a weak organic acid food preservative.](http://www.ncbi.nlm.nih.gov/pubmed/15334557) *Yeast.* 2004; **21:** 927-46.

Mollapour M, Shepherd A, Piper PW. [Novel stress responses facilitate Saccharomyces cerevisiae growth in the presence of the monocarboxylate preservatives.](http://www.ncbi.nlm.nih.gov/pubmed/18240334) *Yeast.* 2008; **25:** 169-77.

Stratford M, Plumridge A, Archer DB. [Decarboxylation of sorbic acid by spoilage yeasts is associated with the PAD1 gene.](http://www.ncbi.nlm.nih.gov/pubmed/17766451) *Appl Environ Microbiol.* 2007; **73:** 6534-42.

Stratford M, Anslow PA. [Evidence that sorbic acid does not inhibit yeast as a classic 'weak acid preservative'.](http://www.ncbi.nlm.nih.gov/pubmed/9812395) *Lett Appl Microbiol.* 1998; **27:** 203-6.

Mira NP, Teixeira MC, Sá-Correia I. [Adaptive response and tolerance to weak acids in Saccharomyces cerevisiae: a genome-wide view.](http://www.ncbi.nlm.nih.gov/pubmed/20955006) *OMICS.* 2010; **14:** 525-40.

Russnak R, Konczal D, McIntire SL. [A family of yeast proteins mediating bidirectional vacuolar amino acid transport.](http://www.ncbi.nlm.nih.gov/pubmed/11274162) *J Biol Chem.* 2001; **276:** 23849-57.

Mielniczki-Pereira AA, Schuch AZ, Bonatto D, Cavalcante CF, Vaitsman DS, Riger CJ, Eleutherio EC, Henriques JA. [The role of the yeast ATP-binding cassette Ycf1p in glutathione and cadmium ion homeostasis during respiratory metabolism.](http://www.ncbi.nlm.nih.gov/pubmed/18602772) *Toxicol Lett.* 2008; **180:** 21-7.

Yenush L, Merchan S, Holmes J, Serrano R. [pH-Responsive, posttranslational regulation of the Trk1 potassium transporter by the type 1-related Ppz1 phosphatase.](http://www.ncbi.nlm.nih.gov/pubmed/16166647) *Mol Cell Biol.* 2005; **25:** 8683-92.

Forment J, Mulet JM, Vicente O, Serrano R. [The yeast SR protein kinase Sky1p modulates salt tolerance, membrane potential and the Trk1,2 potassium transporter.](http://www.ncbi.nlm.nih.gov/pubmed/12225850) *Biochim Biophys Acta.* 2002; **1565:** 36-40.

Erez O, Kahana C. [Deletions of SKY1 or PTK2 in the Saccharomyces cerevisiae trk1Deltatrk2Delta mutant cells exert dual effect on ion homeostasis.](http://www.ncbi.nlm.nih.gov/pubmed/12135613) *Biochem Biophys Res Commun.* 2002; **295:** 1142-9.

Luk EE, Culotta VC. [Manganese superoxide dismutase in Saccharomyces cerevisiae acquires its metal co-factor through a pathway involving the Nramp metal transporter, Smf2p.](http://www.ncbi.nlm.nih.gov/pubmed/11602606) *J Biol Chem.* 2001; **276:** 47556-62.

Luk E, Carroll M, Baker M, Culotta VC. [Manganese activation of superoxide dismutase 2 in Saccharomyces cerevisiae requires MTM1, a member of the mitochondrial carrier family.](http://www.ncbi.nlm.nih.gov/pubmed/12890866) *Proc Natl Acad Sci U S A.* 2003; **100:** 10353-7.

Dürr G, Strayle J, Plemper R, Elbs S, Klee SK, Catty P, Wolf DH, Rudolph HK. [The medial-Golgi ion pump Pmr1 supplies the yeast secretory pathway with Ca2+ and Mn2+ required for glycosylation, sorting, and endoplasmic reticulum-associated protein degradation.](http://www.ncbi.nlm.nih.gov/pubmed/9571246) *Mol Biol Cell.* 1998; **9:** 1149-62.

Supek F, Supekova L, Nelson H, Nelson N. [A yeast manganese transporter related to the macrophage protein involved in conferring resistance to mycobacteria.](http://www.ncbi.nlm.nih.gov/pubmed/8643535) *Proc Natl Acad Sci U S A.* 1996; **93:** 5105-10.

Nakai Y, Nakai M, Lill R, Suzuki T, Hayashi H. [Thio modification of yeast cytosolic tRNA is an iron-sulfur protein-dependent pathway.](http://www.ncbi.nlm.nih.gov/pubmed/17283054) *Mol Cell Biol.* 2007; **27:** 2841-7.

Nakai Y, Umeda N, Suzuki T, Nakai M, Hayashi H, Watanabe K, Kagamiyama H. [Yeast Nfs1p is involved in thio-modification of both mitochondrial and cytoplasmic tRNAs.](http://www.ncbi.nlm.nih.gov/pubmed/14722066) *J Biol Chem.* 2004; **279:** 12363-8.

Wiedemann N, Urzica E, Guiard B, Müller H, Lohaus C, Meyer HE, Ryan MT, Meisinger C, Mühlenhoff U, Lill R, Pfanner N.[Essential role of Isd11 in mitochondrial iron-sulfur cluster synthesis on Isu scaffold proteins.](http://www.ncbi.nlm.nih.gov/pubmed/16341089) *EMBO J.* 2006; **25:** 184-95.

Li L, Bagley D, Ward DM, Kaplan J. [Yap5 is an iron-responsive transcriptional activator that regulates vacuolar iron storage in yeast.](http://www.ncbi.nlm.nih.gov/pubmed/18070921) *Mol Cell Biol.* 2008; **28:** 1326-37.

Outten CE, Albetel AN. [Iron sensing and regulation in Saccharomyces cerevisiae: Ironing out the mechanistic details.](http://www.ncbi.nlm.nih.gov/pubmed/23962819) *Curr Opin Microbiol.* 2013; **16:** 662-8.

Martínez-Pastor M, Vergara SV, Puig S, Thiele DJ. [Negative feedback regulation of the yeast CTH1 and CTH2 mRNA binding proteins is required for adaptation to iron deficiency and iron supplementation.](http://www.ncbi.nlm.nih.gov/pubmed/23530061) *Mol Cell Biol.* 2013; **33:** 2178-87.

Marchler G, Schüller C, Adam G, Ruis H. [A Saccharomyces cerevisiae UAS element controlled by protein kinase A activates transcription in response to a variety of stress conditions.](http://www.ncbi.nlm.nih.gov/pubmed/8387917) *EMBO J.* 1993; **12:** 1997-2003.

Schüller C, Brewster JL, Alexander MR, Gustin MC, Ruis H. [The HOG pathway controls osmotic regulation of transcription via the stress response element (STRE) of the Saccharomyces cerevisiae CTT1 gene.](http://www.ncbi.nlm.nih.gov/pubmed/7523111) *EMBO J.* 1994;**13:** 4382-9.

Lee J, Godon C, Lagniel G, Spector D, Garin J, Labarre J, Toledano MB. [Yap1 and Skn7 control two specialized oxidative stress response regulons in yeast.](http://www.ncbi.nlm.nih.gov/pubmed/10347154) *J Biol Chem.* 1999; **274:** 16040-6.

Wu CY, Bird AJ, Chung LM, Newton MA, Winge DR, Eide DJ. [Differential control of Zap1-regulated genes in response to zinc deficiency in Saccharomyces cerevisiae.](http://www.ncbi.nlm.nih.gov/pubmed/18673560) *BMC Genomics.* 2008; **9:** 370.

Jungmann J, Reins HA, Lee J, Romeo A, Hassett R, Kosman D, Jentsch S. [MAC1, a nuclear regulatory protein related to Cu-dependent transcription factors is involved in Cu/Fe utilization and stress resistance in yeast.](http://www.ncbi.nlm.nih.gov/pubmed/8262047) *EMBO J.* 1993; **12:**5051-6.

Avendaño A, Deluna A, Olivera H, Valenzuela L, Gonzalez A. [GDH3 encodes a glutamate dehydrogenase isozyme, a previously unrecognized route for glutamate biosynthesis in Saccharomyces cerevisiae.](http://www.ncbi.nlm.nih.gov/pubmed/9287019) *J Bacteriol.* 1997; **179:** 5594-7.

Tang Y, Sieg A, Trotter PJ. [¹³C-metabolic enrichment of glutamate in glutamate dehydrogenase mutants of Saccharomyces cerevisiae.](http://www.ncbi.nlm.nih.gov/pubmed/21242068) *Microbiol Res.* 2011; **166:** 521-30.

Phalip V, Kuhn I, Lemoine Y, Jeltsch JM. [Characterization of the biotin biosynthesis pathway in Saccharomyces cerevisiae and evidence for a cluster containing BIO5, a novel gene involved in vitamer uptake.](http://www.ncbi.nlm.nih.gov/pubmed/10333520) *Gene.* 1999; **232:** 43-51.

Ugulava NB, Gibney BR, Jarrett JT. [Biotin synthase contains two distinct iron-sulfur cluster binding sites: chemical and spectroelectrochemical analysis of iron-sulfur cluster interconversions.](http://www.ncbi.nlm.nih.gov/pubmed/11444981) *Biochemistry.* 2001; **40:** 8343-51.

Weider M, Machnik A, Klebl F, Sauer N. [Vhr1p, a new transcription factor from budding yeast, regulates biotin-dependent expression of VHT1 and BIO5.](http://www.ncbi.nlm.nih.gov/pubmed/16533810) *J Biol Chem.* 2006; **281:** 13513-24.

Zhang Y, Lyver ER, Knight SA, Pain D, Lesuisse E, Dancis A. [Mrs3p, Mrs4p, and frataxin provide iron for Fe-S cluster synthesis in mitochondria.](http://www.ncbi.nlm.nih.gov/pubmed/16769722) *J Biol Chem.* 2006; **281:** 22493-502.

Conklin DS, McMaster JA, Culbertson MR, Kung C. [COT1, a gene involved in cobalt accumulation in Saccharomyces cerevisiae.](http://www.ncbi.nlm.nih.gov/pubmed/1508175) *Mol Cell Biol.* 1992; **12:** 3678-88.

Singh A, Kaur N, Kosman DJ. [The metalloreductase Fre6p in Fe-efflux from the yeast vacuole.](http://www.ncbi.nlm.nih.gov/pubmed/17681937) *J Biol Chem.* 2007; **282:**28619-26.

Protchenko O, Ferea T, Rashford J, Tiedeman J, Brown PO, Botstein D, Philpott CC. [Three cell wall mannoproteins facilitate the uptake of iron in Saccharomyces cerevisiae.](http://www.ncbi.nlm.nih.gov/pubmed/11673473) *J Biol Chem.* 2001; **276:** 49244-50.

Li L, Kaplan J. [Defects in the yeast high affinity iron transport system result in increased metal sensitivity because of the increased expression of transporters with a broad transition metal specificity.](http://www.ncbi.nlm.nih.gov/pubmed/9712830) *J Biol Chem.* 1998; **273:** 22181-7.

Dix D, Bridgham J, Broderius M, Eide D. [Characterization of the FET4 protein of yeast. Evidence for a direct role in the transport of iron.](http://www.ncbi.nlm.nih.gov/pubmed/9115232) *J Biol Chem.* 1997; **272:** 11770-7.

Cohen A, Nelson H, Nelson N. [The family of SMF metal ion transporters in yeast cells.](http://www.ncbi.nlm.nih.gov/pubmed/10930410) *J Biol Chem.* 2000; **275:** 33388-94.

Rees EM, Thiele DJ. [Identification of a vacuole-associated metalloreductase and its role in Ctr2-mediated intracellular copper mobilization.](http://www.ncbi.nlm.nih.gov/pubmed/17553781) *J Biol Chem.* 2007; **282:** 21629-38.

Yamaguchi-Iwai Y, Serpe M, Haile D, Yang W, Kosman DJ, Klausner RD, Dancis A. [Homeostatic regulation of copper uptake in yeast via direct binding of MAC1 protein to upstream regulatory sequences of FRE1 and CTR1.](http://www.ncbi.nlm.nih.gov/pubmed/9211922) *J Biol Chem.* 1997; **272:** 17711-8.

Martins LJ, Jensen LT, Simon JR, Keller GL, Winge DR. [Metalloregulation of FRE1 and FRE2 homologs in Saccharomyces cerevisiae.](http://www.ncbi.nlm.nih.gov/pubmed/9726978) *J Biol Chem.* 1998; **273:** 23716-21.

Buchman C, Skroch P, Welch J, Fogel S, Karin M. [The CUP2 gene product, regulator of yeast metallothionein expression, is a copper-activated DNA-binding protein.](http://www.ncbi.nlm.nih.gov/pubmed/2674688) *Mol Cell Biol.* 1989; **9:** 4091-5.

Huibregtse JM, Engelke DR, Thiele DJ. [Copper-induced binding of cellular factors to yeast metallothionein upstream activation sequences.](http://www.ncbi.nlm.nih.gov/pubmed/2643107) *Proc Natl Acad Sci U S A.* 1989; **86:** 65-9.

Szczypka MS, Thiele DJ. [A cysteine-rich nuclear protein activates yeast metallothionein gene transcription.](http://www.ncbi.nlm.nih.gov/pubmed/2651899) *Mol Cell Biol.*1989; **9:** 421-9.

Culotta VC, Howard WR, Liu XF. [CRS5 encodes a metallothionein-like protein in Saccharomyces cerevisiae.](http://www.ncbi.nlm.nih.gov/pubmed/7929222) *J Biol Chem.*1994; **269:** 25295-302.

Butt TR, Sternberg EJ, Gorman JA, Clark P, Hamer D, Rosenberg M, Crooke ST. [Copper metallothionein of yeast, structure of the gene, and regulation of expression.](http://www.ncbi.nlm.nih.gov/pubmed/6374656) *Proc Natl Acad Sci U S A.* 1984; **81:** 3332-6.

Yuan DS, Stearman R, Dancis A, Dunn T, Beeler T, Klausner RD. [The Menkes/Wilson disease gene homologue in yeast provides copper to a ceruloplasmin-like oxidase required for iron uptake.](http://www.ncbi.nlm.nih.gov/pubmed/7708696) *Proc Natl Acad Sci U S A.* 1995; **92:** 2632-6.

Gralla EB, Thiele DJ, Silar P, Valentine JS. [ACE1, a copper-dependent transcription factor, activates expression of the yeast copper, zinc superoxide dismutase gene.](http://www.ncbi.nlm.nih.gov/pubmed/1924315) *Proc Natl Acad Sci U S A.* 1991; **88:** 8558-62.

Cobine PA, Ojeda LD, Rigby KM, Winge DR. [Yeast contain a non-proteinaceous pool of copper in the mitochondrial matrix.](http://www.ncbi.nlm.nih.gov/pubmed/14729672) *J Biol Chem.* 2004; **279:** 14447-55.

Portnoy ME, Schmidt PJ, Rogers RS, Culotta VC. [Metal transporters that contribute copper to metallochaperones in Saccharomyces cerevisiae.](http://www.ncbi.nlm.nih.gov/pubmed/11523804) *Mol Genet Genomics.* 2001; **265:** 873-82.

Regenberg B, Holmberg S, Olsen LD, Kielland-Brandt MC. [Dip5p mediates high-affinity and high-capacity transport of L-glutamate and L-aspartate in Saccharomyces cerevisiae.](http://www.ncbi.nlm.nih.gov/pubmed/9508791) *Curr Genet.* 1998; **33:** 171-7.

Courchesne WE, Ozturk S. [Amiodarone induces a caffeine-inhibited, MID1-depedent rise in free cytoplasmic calcium in Saccharomyces cerevisiae.](http://www.ncbi.nlm.nih.gov/pubmed/12492866) *Mol Microbiol.* 2003; **47:** 223-34.

Gupta SS, Ton VK, Beaudry V, Rulli S, Cunningham K, Rao R. [Antifungal activity of amiodarone is mediated by disruption of calcium homeostasis.](http://www.ncbi.nlm.nih.gov/pubmed/12754197) *J Biol Chem.* 2003; **278:** 28831-9.

Zhang YQ, Rao R. [A spoke in the wheel: calcium spikes disrupt yeast cell cycle.](http://www.ncbi.nlm.nih.gov/pubmed/18414024) *Cell Cycle.* 2008; **7:** 870-3.

Matsuura I, Ishihara K, Nakai Y, Yazawa M, Toda H, Yagi K. [A site-directed mutagenesis study of yeast calmodulin.](http://www.ncbi.nlm.nih.gov/pubmed/2016268) *J Biochem.* 1991; **109:** 190-7.

Bonilla M, Cunningham KW. [Mitogen-activated protein kinase stimulation of Ca(2+) signaling is required for survival of endoplasmic reticulum stress in yeast.](http://www.ncbi.nlm.nih.gov/pubmed/14517337) *Mol Biol Cell.* 2003; **14:** 4296-305.

Kruegel U, Robison B, Dange T, Kahlert G, Delaney JR, Kotireddy S, Tsuchiya M, Tsuchiyama S, Murakami CJ, Schleit J, Sutphin G, Carr D, Tar K, Dittmar G, Kaeberlein M, Kennedy BK, Schmidt M. [Elevated proteasome capacity extends replicative lifespan in Saccharomyces cerevisiae.](http://www.ncbi.nlm.nih.gov/pubmed/21931558) *PLoS Genet.* 2011; **7:** e1002253.

Kaiser P, Sia RA, Bardes EG, Lew DJ, Reed SI. [Cdc34 and the F-box protein Met30 are required for degradation of the Cdk-inhibitory kinase Swe1.](http://www.ncbi.nlm.nih.gov/pubmed/9716410) *Genes Dev.* 1998; **12:** 2587-97.

Bouquin N, Johnson AL, Morgan BA, Johnston LH. [Association of the cell cycle transcription factor Mbp1 with the Skn7 response regulator in budding yeast.](http://www.ncbi.nlm.nih.gov/pubmed/10512874) *Mol Biol Cell.* 1999; **10:** 3389-400.

McMillan JN, Longtine MS, Sia RA, Theesfeld CL, Bardes ES, Pringle JR, Lew DJ. [The morphogenesis checkpoint in Saccharomyces cerevisiae: cell cycle control of Swe1p degradation by Hsl1p and Hsl7p.](http://www.ncbi.nlm.nih.gov/pubmed/10490630) *Mol Cell Biol.* 1999; **19:** 6929-39.

Asano S, Park JE, Sakchaisri K, Yu LR, Song S, Supavilai P, Veenstra TD, Lee KS. [Concerted mechanism of Swe1/Wee1 regulation by multiple kinases in budding yeast.](http://www.ncbi.nlm.nih.gov/pubmed/15920482) *EMBO J.* 2005; **24:** 2194-204.

Heath VL, Shaw SL, Roy S, Cyert MS. [Hph1p and Hph2p, novel components of calcineurin-mediated stress responses in Saccharomyces cerevisiae.](http://www.ncbi.nlm.nih.gov/pubmed/15189990) *Eukaryot Cell.* 2004; **3:** 695-704.

Takatsume Y, Ohdate T, Maeta K, Nomura W, Izawa S, Inoue Y. [Calcineurin/Crz1 destabilizes Msn2 and Msn4 in the nucleus in response to Ca(2+) in Saccharomyces cerevisiae.](http://www.ncbi.nlm.nih.gov/pubmed/20121702) *Biochem J.* 2010; **427:** 275-87.

Tatebayashi K, Tanaka K, Yang HY, Yamamoto K, Matsushita Y, Tomida T, Imai M, Saito H. [Transmembrane mucins Hkr1 and Msb2 are putative osmosensors in the SHO1 branch of yeast HOG pathway.](http://www.ncbi.nlm.nih.gov/pubmed/17627274) *EMBO J.* 2007; **26:** 3521-33.

Wu C, Whiteway M, Thomas DY, Leberer E. [Molecular characterization of Ste20p, a potential mitogen-activated protein or extracellular signal-regulated kinase kinase (MEK) kinase kinase from Saccharomyces cerevisiae.](http://www.ncbi.nlm.nih.gov/pubmed/7608157) *J Biol Chem.* 1995;**270:** 15984-92.

Ault AD, Fassler JS, Deschenes RJ. [Altered phosphotransfer in an activated mutant of the Saccharomyces cerevisiae two-component osmosensor Sln1p.](http://www.ncbi.nlm.nih.gov/pubmed/12455952) *Eukaryot Cell.* 2002; **1:** 174-80.

Ferrigno P, Posas F, Koepp D, Saito H, Silver PA. [Regulated nucleo/cytoplasmic exchange of HOG1 MAPK requires the importin beta homologs NMD5 and XPO1.](http://www.ncbi.nlm.nih.gov/pubmed/9755161) *EMBO J.* 1998; **17:** 5606-14.

Stade K, Ford CS, Guthrie C, Weis K. [Exportin 1 (Crm1p) is an essential nuclear export factor.](http://www.ncbi.nlm.nih.gov/pubmed/9323132) *Cell.* 1997; **90:** 1041-50.

Mattison CP, Ota IM. [Two protein tyrosine phosphatases, Ptp2 and Ptp3, modulate the subcellular localization of the Hog1 MAP kinase in yeast.](http://www.ncbi.nlm.nih.gov/pubmed/10817757) *Genes Dev.* 2000; **14:** 1229-35.

Warmka J, Hanneman J, Lee J, Amin D, Ota I. [Ptc1, a type 2C Ser/Thr phosphatase, inactivates the HOG pathway by dephosphorylating the mitogen-activated protein kinase Hog1.](http://www.ncbi.nlm.nih.gov/pubmed/11113180) *Mol Cell Biol.* 2001; **21:** 51-60.

Young C, Mapes J, Hanneman J, Al-Zarban S, Ota I. [Role of Ptc2 type 2C Ser/Thr phosphatase in yeast high-osmolarity glycerol pathway inactivation.](http://www.ncbi.nlm.nih.gov/pubmed/12477803) *Eukaryot Cell.* 2002; **1:** 1032-40.

Wurgler-Murphy SM, Maeda T, Witten EA, Saito H. [Regulation of the Saccharomyces cerevisiae HOG1 mitogen-activated protein kinase by the PTP2 and PTP3 protein tyrosine phosphatases.](http://www.ncbi.nlm.nih.gov/pubmed/9032256) *Mol Cell Biol.* 1997; **17:** 1289-97.

Brewster JL, de Valoir T, Dwyer ND, Winter E, Gustin MC. [An osmosensing signal transduction pathway in yeast.](http://www.ncbi.nlm.nih.gov/pubmed/7681220) *Science.*1993; **259:** 1760-3.

Posas F, Saito H. [Activation of the yeast SSK2 MAP kinase kinase kinase by the SSK1 two-component response regulator.](http://www.ncbi.nlm.nih.gov/pubmed/9482735) *EMBO J.* 1998; **17:** 1385-94.

Janiak-Spens F, West AH. [Functional roles of conserved amino acid residues surrounding the phosphorylatable histidine of the yeast phosphorelay protein YPD1.](http://www.ncbi.nlm.nih.gov/pubmed/10931311) *Mol Microbiol.* 2000; **37:** 136-44.

Muller EM, Locke EG, Cunningham KW. [Differential regulation of two Ca(2+) influx systems by pheromone signaling in Saccharomyces cerevisiae.](http://www.ncbi.nlm.nih.gov/pubmed/11779794) *Genetics.* 2001; **159:** 1527-38.

Andrews BJ, Moore LA. [Interaction of the yeast Swi4 and Swi6 cell cycle regulatory proteins in vitro.](http://www.ncbi.nlm.nih.gov/pubmed/1465410) *Proc Natl Acad Sci U S A.* 1992; **89:** 11852-6.

Koch C, Moll T, Neuberg M, Ahorn H, Nasmyth K. [A role for the transcription factors Mbp1 and Swi4 in progression from G1 to S phase.](http://www.ncbi.nlm.nih.gov/pubmed/8372350) *Science.* 1993; **261:** 1551-7.

Mizunuma M, Hirata D, Miyahara K, Tsuchiya E, Miyakawa T. [Role of calcineurin and Mpk1 in regulating the onset of mitosis in budding yeast.](http://www.ncbi.nlm.nih.gov/pubmed/9521328) *Nature.* 1998; **392:** 303-6.

Nishizawa M, Tanigawa M, Hayashi M, Maeda T, Yazaki Y, Saeki Y, Toh-e A. [Pho85 kinase, a cyclin-dependent kinase, regulates nuclear accumulation of the Rim101 transcription factor in the stress response of Saccharomyces cerevisiae.](http://www.ncbi.nlm.nih.gov/pubmed/20382759) *Eukaryot Cell.* 2010; **9:** 943-51.

Feldman RM, Correll CC, Kaplan KB, Deshaies RJ. [A complex of Cdc4p, Skp1p, and Cdc53p/cullin catalyzes ubiquitination of the phosphorylated CDK inhibitor Sic1p.](http://www.ncbi.nlm.nih.gov/pubmed/9346239) *Cell.* 1997; **91:** 221-30.

Skowyra D, Craig KL, Tyers M, Elledge SJ, Harper JW. [F-box proteins are receptors that recruit phosphorylated substrates to the SCF ubiquitin-ligase complex.](http://www.ncbi.nlm.nih.gov/pubmed/9346238) *Cell.* 1997; **91:** 209-19.

Griffioen G, Swinnen S, Thevelein JM. [Feedback inhibition on cell wall integrity signaling by Zds1 involves Gsk3 phosphorylation of a cAMP-dependent protein kinase regulatory subunit.](http://www.ncbi.nlm.nih.gov/pubmed/12704202) *J Biol Chem.* 2003; **278:** 23460-71.

Clipstone NA, Fiorentino DF, Crabtree GR. [Molecular analysis of the interaction of calcineurin with drug-immunophilin complexes.](http://www.ncbi.nlm.nih.gov/pubmed/7523407) *J Biol Chem.* 1994; **269:** 26431-7.

Cardenas ME, Hemenway C, Muir RS, Ye R, Fiorentino D, Heitman J. [Immunophilins interact with calcineurin in the absence of exogenous immunosuppressive ligands.](http://www.ncbi.nlm.nih.gov/pubmed/7529175) *EMBO J.* 1994; **13:** 5944-57.

Zhu D, Cardenas ME, Heitman J. [Myristoylation of calcineurin B is not required for function or interaction with immunophilin-immunosuppressant complexes in the yeast Saccharomyces cerevisiae.](http://www.ncbi.nlm.nih.gov/pubmed/7559604) *J Biol Chem.* 1995; **270:** 24831-8.

Cardenas ME, Lim E, Heitman J. [Mutations that perturb cyclophilin A ligand binding pocket confer cyclosporin A resistance in Saccharomyces cerevisiae.](http://www.ncbi.nlm.nih.gov/pubmed/7673124) *J Biol Chem.* 1995; **270:** 20997-1002.

Mazur P, Morin N, Baginsky W, el-Sherbeini M, Clemas JA, Nielsen JB, Foor F. [Differential expression and function of two homologous subunits of yeast 1,3-beta-D-glucan synthase.](http://www.ncbi.nlm.nih.gov/pubmed/7565718) *Mol Cell Biol.* 1995; **15:** 5671-81.

Zhao C, Jung US, Garrett-Engele P, Roe T, Cyert MS, Levin DE. [Temperature-induced expression of yeast FKS2 is under the dual control of protein kinase C and calcineurin.](http://www.ncbi.nlm.nih.gov/pubmed/9447998) *Mol Cell Biol.* 1998; **18:** 1013-22.

Tenney KA, Glover CV. [Transcriptional regulation of the S. cerevisiae ENA1 gene by casein kinase II.](http://www.ncbi.nlm.nih.gov/pubmed/10094405) *Mol Cell Biochem.*1999; **191:** 161-7.

Pausch MH, Kaim D, Kunisawa R, Admon A, Thorner J. [Multiple Ca2+/calmodulin-dependent protein kinase genes in a unicellular eukaryote.](http://www.ncbi.nlm.nih.gov/pubmed/2026147) *EMBO J.* 1991; **10:** 1511-22.

Cavinder B, Trail F. [Role of Fig1, a component of the low-affinity calcium uptake system, in growth and sexual development of filamentous fungi.](http://www.ncbi.nlm.nih.gov/pubmed/22635922) *Eukaryot Cell.* 2012; **11:** 978-88.

Muller EM, Mackin NA, Erdman SE, Cunningham KW. [Fig1p facilitates Ca2+ influx and cell fusion during mating of Saccharomyces cerevisiae.](http://www.ncbi.nlm.nih.gov/pubmed/12878605) *J Biol Chem.* 2003; **278:** 38461-9.

Zhang S, Zheng H, Long N, Carbó N, Chen P, Aguilar PS, Lu L. [FigA, a putative homolog of low-affinity calcium system member Fig1 in Saccharomyces cerevisiae, is involved in growth and asexual and sexual development in Aspergillus nidulans.](http://www.ncbi.nlm.nih.gov/pubmed/24376003) *Eukaryot Cell.* 2014; **13:** 295-303.

Wang H, Liang Y, Zhang B, Zheng W, Xing L, Li M. [Alkaline stress triggers an immediate calcium fluctuation in Candida albicans mediated by Rim101p and Crz1p transcription factors.](http://www.ncbi.nlm.nih.gov/pubmed/21457451) *FEMS Yeast Res.* 2011; **11:** 430-9.

Han GS, Johnston CN, Chen X, Athenstaedt K, Daum G, Carman GM. [Regulation of the Saccharomyces cerevisiae DPP1-encoded diacylglycerol pyrophosphate phosphatase by zinc.](http://www.ncbi.nlm.nih.gov/pubmed/11139591) *J Biol Chem.* 2001; **276:** 10126-33.

Han GS, Johnston CN, Carman GM. [Vacuole membrane topography of the DPP1-encoded diacylglycerol pyrophosphate phosphatase catalytic site from Saccharomyces cerevisiae.](http://www.ncbi.nlm.nih.gov/pubmed/14630917) *J Biol Chem.* 2004; **279:** 5338-45.

Oshiro J, Han GS, Carman GM. [Diacylglycerol pyrophosphate phosphatase in Saccharomyces cerevisiae.](http://www.ncbi.nlm.nih.gov/pubmed/14642771) *Biochim Biophys Acta.* 2003; **1635:** 1-9.

Rouillon A, Barbey R, Patton EE, Tyers M, Thomas D. [Feedback-regulated degradation of the transcriptional activator Met4 is triggered by the SCF(Met30 )complex.](http://www.ncbi.nlm.nih.gov/pubmed/10637232) *EMBO J.* 2000; **19:** 282-94.

Soto-Cardalda A, Fakas S, Pascual F, Choi HS, Carman GM. [Phosphatidate phosphatase plays role in zinc-mediated regulation of phospholipid synthesis in yeast.](http://www.ncbi.nlm.nih.gov/pubmed/22128164) *J Biol Chem.* 2012; **287:** 968-77.

Drewke C, Ciriacy M. [Overexpression, purification and properties of alcohol dehydrogenase IV from Saccharomyces cerevisiae.](http://www.ncbi.nlm.nih.gov/pubmed/3282541) *Biochim Biophys Acta.* 1988; **950:** 54-60.

Bird AJ, Gordon M, Eide DJ, Winge DR. [Repression of ADH1 and ADH3 during zinc deficiency by Zap1-induced intergenic RNA transcripts.](http://www.ncbi.nlm.nih.gov/pubmed/17139254) *EMBO J.* 2006; **25:** 5726-34.

Wu YH, Frey AG, Eide DJ. [Transcriptional regulation of the Zrg17 zinc transporter of the yeast secretory pathway.](http://www.ncbi.nlm.nih.gov/pubmed/21250939)*Biochem J.* 2011; **435:** 259-66.

Waters BM, Eide DJ. [Combinatorial control of yeast FET4 gene expression by iron, zinc, and oxygen.](http://www.ncbi.nlm.nih.gov/pubmed/12095998) *J Biol Chem.* 2002;**277:** 33749-57.

Jensen LT, Culotta VC. [Regulation of Saccharomyces cerevisiae FET4 by oxygen and iron.](http://www.ncbi.nlm.nih.gov/pubmed/12051835) *J Mol Biol.* 2002; **318:** 251-60.

Lyons TJ, Gasch AP, Gaither LA, Botstein D, Brown PO, Eide DJ. [Genome-wide characterization of the Zap1p zinc-responsive regulon in yeast.](http://www.ncbi.nlm.nih.gov/pubmed/10884426) *Proc Natl Acad Sci U S A.* 2000; **97:** 7957-62.

Zhao H, Eide DJ. [Zap1p, a metalloregulatory protein involved in zinc-responsive transcriptional regulation in Saccharomyces cerevisiae.](http://www.ncbi.nlm.nih.gov/pubmed/9271382) *Mol Cell Biol.* 1997; **17:** 5044-52.

Bird AJ, Blankman E, Stillman DJ, Eide DJ, Winge DR. [The Zap1 transcriptional activator also acts as a repressor by binding downstream of the TATA box in ZRT2.](http://www.ncbi.nlm.nih.gov/pubmed/14976557) *EMBO J.* 2004; **23:** 1123-32.

Zhao H, Eide D. [The yeast ZRT1 gene encodes the zinc transporter protein of a high-affinity uptake system induced by zinc limitation.](http://www.ncbi.nlm.nih.gov/pubmed/8637895) *Proc Natl Acad Sci U S A.* 1996; **93:** 2454-8.

Zhao H, Eide D. [The ZRT2 gene encodes the low affinity zinc transporter in Saccharomyces cerevisiae.](http://www.ncbi.nlm.nih.gov/pubmed/8798516) *J Biol Chem.*1996; **271:** 23203-10.

Harashima T, Heitman J. [Galpha subunit Gpa2 recruits kelch repeat subunits that inhibit receptor-G protein coupling during cAMP-induced dimorphic transitions in Saccharomyces cerevisiae.](http://www.ncbi.nlm.nih.gov/pubmed/16030250) *Mol Biol Cell.* 2005; **16:** 4557-71.

Graczyk D, Debski J, Muszyńska G, Bretner M, Lefebvre O, Boguta M. [Casein kinase II-mediated phosphorylation of general repressor Maf1 triggers RNA polymerase III activation.](http://www.ncbi.nlm.nih.gov/pubmed/21383183) *Proc Natl Acad Sci U S A.* 2011; **108:** 4926-31.

Fernández-Tornero C, Böttcher B, Rashid UJ, Müller CW. [Analyzing RNA polymerase III by electron cryomicroscopy.](http://www.ncbi.nlm.nih.gov/pubmed/21881405) *RNA Biol.* 2011; **8:** 760-5.

Schramm L, Hernandez N. [Recruitment of RNA polymerase III to its target promoters.](http://www.ncbi.nlm.nih.gov/pubmed/12381659) *Genes Dev.* 2002; **16:** 2593-620.

Omnus DJ, Pfirrmann T, Andréasson C, Ljungdahl PO. [A phosphodegron controls nutrient-induced proteasomal activation of the signaling protease Ssy5.](http://www.ncbi.nlm.nih.gov/pubmed/21653827) *Mol Biol Cell.* 2011; **22:** 2754-65.

Park TS, Ostrander DB, Pappas A, Carman GM. [Identification of Ser424 as the protein kinase A phosphorylation site in CTP synthetase from Saccharomyces cerevisiae.](http://www.ncbi.nlm.nih.gov/pubmed/10393561) *Biochemistry.* 1999; **38:** 8839-48.

Nadkarni AK, McDonough VM, Yang WL, Stukey JE, Ozier-Kalogeropoulos O, Carman GM. [Differential biochemical regulation of the URA7- and URA8-encoded CTP synthetases from Saccharomyces cerevisiae.](http://www.ncbi.nlm.nih.gov/pubmed/7559626) *J Biol Chem.* 1995;**270:** 24982-8.

Yang WL, Bruno ME, Carman GM. [Regulation of yeast CTP synthetase activity by protein kinase C.](http://www.ncbi.nlm.nih.gov/pubmed/8626655) *J Biol Chem.* 1996;**271:** 11113-9.

Tu J, Carlson M. [REG1 binds to protein phosphatase type 1 and regulates glucose repression in Saccharomyces cerevisiae.](http://www.ncbi.nlm.nih.gov/pubmed/8846786) *EMBO J.* 1995; **14:** 5939-46.

Alms GR, Sanz P, Carlson M, Haystead TA. [Reg1p targets protein phosphatase 1 to dephosphorylate hexokinase II in Saccharomyces cerevisiae: characterizing the effects of a phosphatase subunit on the yeast proteome.](http://www.ncbi.nlm.nih.gov/pubmed/10428955) *EMBO J.*1999; **18:** 4157-68.

Sanz P, Alms GR, Haystead TA, Carlson M. [Regulatory interactions between the Reg1-Glc7 protein phosphatase and the Snf1 protein kinase.](http://www.ncbi.nlm.nih.gov/pubmed/10648618) *Mol Cell Biol.* 2000; **20:** 1321-8.

Klein C, Struhl K. [Protein kinase A mediates growth-regulated expression of yeast ribosomal protein genes by modulating RAP1 transcriptional activity.](http://www.ncbi.nlm.nih.gov/pubmed/8114723) *Mol Cell Biol.* 1994; **14:** 1920-8.

Kataoka T, Broek D, Wigler M. [DNA sequence and characterization of the S. cerevisiae gene encoding adenylate cyclase.](http://www.ncbi.nlm.nih.gov/pubmed/2934138)*Cell.* 1985; **43:** 493-505.

Colombo S, Ronchetti D, Thevelein JM, Winderickx J, Martegani E. [Activation state of the Ras2 protein and glucose-induced signaling in Saccharomyces cerevisiae.](http://www.ncbi.nlm.nih.gov/pubmed/15339905) *J Biol Chem.* 2004; **279:** 46715-22.

Broek D, Toda T, Michaeli T, Levin L, Birchmeier C, Zoller M, Powers S, Wigler M. [The S. cerevisiae CDC25 gene product regulates the RAS/adenylate cyclase pathway.](http://www.ncbi.nlm.nih.gov/pubmed/3545497) *Cell.* 1987; **48:** 789-99.

Créchet JB, Poullet P, Mistou MY, Parmeggiani A, Camonis J, Boy-Marcotte E, Damak F, Jacquet M. [Enhancement of the GDP-GTP exchange of RAS proteins by the carboxyl-terminal domain of SCD25.](http://www.ncbi.nlm.nih.gov/pubmed/2188363) *Science.* 1990; **248:** 866-8.

Jones S, Vignais ML, Broach JR. [The CDC25 protein of Saccharomyces cerevisiae promotes exchange of guanine nucleotides bound to ras.](http://www.ncbi.nlm.nih.gov/pubmed/2017169) *Mol Cell Biol.* 1991; **11:** 2641-6.

Tanaka K, Matsumoto K, Toh-E A. [IRA1, an inhibitory regulator of the RAS-cyclic AMP pathway in Saccharomyces cerevisiae.](http://www.ncbi.nlm.nih.gov/pubmed/2540426) *Mol Cell Biol.* 1989; **9:** 757-68.

Tanaka K, Nakafuku M, Satoh T, Marshall MS, Gibbs JB, Matsumoto K, Kaziro Y, Toh-e A. [S. cerevisiae genes IRA1 and IRA2 encode proteins that may be functionally equivalent to mammalian ras GTPase activating protein.](http://www.ncbi.nlm.nih.gov/pubmed/2178777) *Cell.* 1990; **60:**803-7.

Tanaka K, Nakafuku M, Tamanoi F, Kaziro Y, Matsumoto K, Toh-e A. [IRA2, a second gene of Saccharomyces cerevisiae that encodes a protein with a domain homologous to mammalian ras GTPase-activating protein.](http://www.ncbi.nlm.nih.gov/pubmed/2164637) *Mol Cell Biol.* 1990;**10:** 4303-13.

Mattila PK, Quintero-Monzon O, Kugler J, Moseley JB, Almo SC, Lappalainen P, Goode BL. [A high-affinity interaction with ADP-actin monomers underlies the mechanism and in vivo function of Srv2/cyclase-associated protein.](http://www.ncbi.nlm.nih.gov/pubmed/15356265) *Mol Biol Cell.* 2004; **15:** 5158-71.

Jiang R, Carlson M. [The Snf1 protein kinase and its activating subunit, Snf4, interact with distinct domains of the Sip1/Sip2/Gal83 component in the kinase complex.](http://www.ncbi.nlm.nih.gov/pubmed/9121458) *Mol Cell Biol.* 1997; **17:** 2099-106.

Vincent O, Townley R, Kuchin S, Carlson M. [Subcellular localization of the Snf1 kinase is regulated by specific beta subunits and a novel glucose signaling mechanism.](http://www.ncbi.nlm.nih.gov/pubmed/11331606) *Genes Dev.* 2001; **15:** 1104-14.

Zhang Y, McCartney RR, Chandrashekarappa DG, Mangat S, Schmidt MC. [Reg1 protein regulates phosphorylation of all three Snf1 isoforms but preferentially associates with the Gal83 isoform.](http://www.ncbi.nlm.nih.gov/pubmed/22002657) *Eukaryot Cell.* 2011; **10:** 1628-36.

Leech A, Nath N, McCartney RR, Schmidt MC. [Isolation of mutations in the catalytic domain of the snf1 kinase that render its activity independent of the snf4 subunit.](http://www.ncbi.nlm.nih.gov/pubmed/12684376) *Eukaryot Cell.* 2003; **2:** 265-73.

Jiang R, Carlson M. [Glucose regulates protein interactions within the yeast SNF1 protein kinase complex.](http://www.ncbi.nlm.nih.gov/pubmed/8985180) *Genes Dev.*1996; **10:** 3105-15.

Santangelo GM. [Glucose signaling in Saccharomyces cerevisiae.](http://www.ncbi.nlm.nih.gov/pubmed/16524925) *Microbiol Mol Biol Rev.* 2006; **70:** 253-82.

Randez-Gil F, Sanz P, Entian KD, Prieto JA. [Carbon source-dependent phosphorylation of hexokinase PII and its role in the glucose-signaling response in yeast.](http://www.ncbi.nlm.nih.gov/pubmed/9566913) *Mol Cell Biol.* 1998; **18:** 2940-8.

Fernández-García P, Peláez R, Herrero P, Moreno F. [Phosphorylation of yeast hexokinase 2 regulates its nucleocytoplasmic shuttling.](http://www.ncbi.nlm.nih.gov/pubmed/23066030) *J Biol Chem.* 2012; **287:** 42151-64.

Newcomb LL, Hall DD, Heideman W. [AZF1 is a glucose-dependent positive regulator of CLN3 transcription in Saccharomyces cerevisiae.](http://www.ncbi.nlm.nih.gov/pubmed/11839825) *Mol Cell Biol.* 2002; **22:** 1607-14.

Hall DD, Markwardt DD, Parviz F, Heideman W. [Regulation of the Cln3-Cdc28 kinase by cAMP in Saccharomyces cerevisiae.](http://www.ncbi.nlm.nih.gov/pubmed/9687505) *EMBO J.* 1998; **17:** 4370-8.

Newcomb LL, Diderich JA, Slattery MG, Heideman W. [Glucose regulation of Saccharomyces cerevisiae cell cycle genes.](http://www.ncbi.nlm.nih.gov/pubmed/12582131)*Eukaryot Cell.* 2003; **2:** 143-9.

Ma P, Wera S, Van Dijck P, Thevelein JM. [The PDE1-encoded low-affinity phosphodiesterase in the yeast Saccharomyces cerevisiae has a specific function in controlling agonist-induced cAMP signaling.](http://www.ncbi.nlm.nih.gov/pubmed/9880329) *Mol Biol Cell.* 1999;**10:** 91-104.

Mbonyi K, van Aelst L, Argüelles JC, Jans AW, Thevelein JM. [Glucose-induced hyperaccumulation of cyclic AMP and defective glucose repression in yeast strains with reduced activity of cyclic AMP-dependent protein kinase.](http://www.ncbi.nlm.nih.gov/pubmed/2201893) *Mol Cell Biol.* 1990; **10:** 4518-23.

Rolland F, Winderickx J, Thevelein JM. [Glucose-sensing and -signalling mechanisms in yeast.](http://www.ncbi.nlm.nih.gov/pubmed/12702307) *FEMS Yeast Res.* 2002; **2:**183-201.

Budhwar R, Fang G, Hirsch JP. [Kelch repeat proteins control yeast PKA activity in response to nutrient availability.](http://www.ncbi.nlm.nih.gov/pubmed/21311222) *Cell Cycle.* 2011; **10:** 767-70.

Tamanoi F. [Ras signaling in yeast.](http://www.ncbi.nlm.nih.gov/pubmed/21779494) *Genes Cancer.* 2011; **2:** 210-5.

Griffioen G, Anghileri P, Imre E, Baroni MD, Ruis H. [Nutritional control of nucleocytoplasmic localization of cAMP-dependent protein kinase catalytic and regulatory subunits in Saccharomyces cerevisiae.](http://www.ncbi.nlm.nih.gov/pubmed/10625697) *J Biol Chem.* 2000; **275:**1449-56.

Werner-Washburne M, Brown D, Braun E. [Bcy1, the regulatory subunit of cAMP-dependent protein kinase in yeast, is differentially modified in response to the physiological status of the cell.](http://www.ncbi.nlm.nih.gov/pubmed/1655793) *J Biol Chem.* 1991; **266:** 19704-9.

Kuret J, Johnson KE, Nicolette C, Zoller MJ. [Mutagenesis of the regulatory subunit of yeast cAMP-dependent protein kinase. Isolation of site-directed mutants with altered binding affinity for catalytic subunit.](http://www.ncbi.nlm.nih.gov/pubmed/3288630) *J Biol Chem.* 1988; **263:**9149-54.

Kassis S, Melhuish T, Annan RS, Chen SL, Lee JC, Livi GP, Creasy CL. [Saccharomyces cerevisiae Yak1p protein kinase autophosphorylates on tyrosine residues and phosphorylates myelin basic protein on a C-terminal serine residue.](http://www.ncbi.nlm.nih.gov/pubmed/10816418)*Biochem J.* 2000; **348 Pt 2:** 263-72.

Schmelzle T, Beck T, Martin DE, Hall MN. [Activation of the RAS/cyclic AMP pathway suppresses a TOR deficiency in yeast.](http://www.ncbi.nlm.nih.gov/pubmed/14673167) *Mol Cell Biol.* 2004; **24:** 338-51.

Cameroni E, Hulo N, Roosen J, Winderickx J, De Virgilio C. [The novel yeast PAS kinase Rim 15 orchestrates G0-associated antioxidant defense mechanisms.](http://www.ncbi.nlm.nih.gov/pubmed/15300954) *Cell Cycle.* 2004; **3:** 462-8.

Fujiyama A, Tamanoi F. [Processing and fatty acid acylation of RAS1 and RAS2 proteins in Saccharomyces cerevisiae.](http://www.ncbi.nlm.nih.gov/pubmed/3513173)*Proc Natl Acad Sci U S A.* 1986; **83:** 1266-70.

Lobo S, Greentree WK, Linder ME, Deschenes RJ. [Identification of a Ras palmitoyltransferase in Saccharomyces cerevisiae.](http://www.ncbi.nlm.nih.gov/pubmed/12193598) *J Biol Chem.* 2002; **277:** 41268-73.

Goodman LE, Judd SR, Farnsworth CC, Powers S, Gelb MH, Glomset JA, Tamanoi F. [Mutants of Saccharomyces cerevisiae defective in the farnesylation of Ras proteins.](http://www.ncbi.nlm.nih.gov/pubmed/2124698) *Proc Natl Acad Sci U S A.* 1990; **87:** 9665-9.

Hrycyna CA, Sapperstein SK, Clarke S, Michaelis S. [The Saccharomyces cerevisiae STE14 gene encodes a methyltransferase that mediates C-terminal methylation of a-factor and RAS proteins.](http://www.ncbi.nlm.nih.gov/pubmed/2050108) *EMBO J.* 1991; **10:** 1699-709.

Fujiyama A, Tamanoi F. [RAS2 protein of Saccharomyces cerevisiae undergoes removal of methionine at N terminus and removal of three amino acids at C terminus.](http://www.ncbi.nlm.nih.gov/pubmed/2406252) *J Biol Chem.* 1990; **265:** 3362-8.

Liu Y, Xu X, Carlson M. [Interaction of SNF1 protein kinase with its activating kinase Sak1.](http://www.ncbi.nlm.nih.gov/pubmed/21216941) *Eukaryot Cell.* 2011; **10:** 313-9.

Nath N, McCartney RR, Schmidt MC. [Yeast Pak1 kinase associates with and activates Snf1.](http://www.ncbi.nlm.nih.gov/pubmed/12748292) *Mol Cell Biol.* 2003; **23:**3909-17.

Hong SP, Leiper FC, Woods A, Carling D, Carlson M. [Activation of yeast Snf1 and mammalian AMP-activated protein kinase by upstream kinases.](http://www.ncbi.nlm.nih.gov/pubmed/12847291) *Proc Natl Acad Sci U S A.* 2003; **100:** 8839-43.

Sutherland CM, Hawley SA, McCartney RR, Leech A, Stark MJ, Schmidt MC, Hardie DG. [Elm1p is one of three upstream kinases for the Saccharomyces cerevisiae SNF1 complex.](http://www.ncbi.nlm.nih.gov/pubmed/12906789) *Curr Biol.* 2003; **13:** 1299-305.

McCartney RR, Schmidt MC. [Regulation of Snf1 kinase. Activation requires phosphorylation of threonine 210 by an upstream kinase as well as a distinct step mediated by the Snf4 subunit.](http://www.ncbi.nlm.nih.gov/pubmed/11486005) *J Biol Chem.* 2001; **276:** 36460-6.

Ozcan S, Dover J, Rosenwald AG, Wölfl S, Johnston M. [Two glucose transporters in Saccharomyces cerevisiae are glucose sensors that generate a signal for induction of gene expression.](http://www.ncbi.nlm.nih.gov/pubmed/8901598) *Proc Natl Acad Sci U S A.* 1996; **93:** 12428-32.

Rubin-Bejerano I, Sagee S, Friedman O, Pnueli L, Kassir Y. [The in vivo activity of Ime1, the key transcriptional activator of meiosis-specific genes in Saccharomyces cerevisiae, is inhibited by the cyclic AMP/protein kinase A signal pathway through the glycogen synthase kinase 3-beta homolog Rim11.](http://www.ncbi.nlm.nih.gov/pubmed/15282298) *Mol Cell Biol.* 2004; **24:** 6967-79.

Slattery MG, Liko D, Heideman W. [The function and properties of the Azf1 transcriptional regulator change with growth conditions in Saccharomyces cerevisiae.](http://www.ncbi.nlm.nih.gov/pubmed/16467472) *Eukaryot Cell.* 2006; **5:** 313-20.

Harashima T, Heitman J. [The Galpha protein Gpa2 controls yeast differentiation by interacting with kelch repeat proteins that mimic Gbeta subunits.](http://www.ncbi.nlm.nih.gov/pubmed/12150916) *Mol Cell.* 2002; **10:** 163-73.

Ishiwata M, Kurahashi H, Nakamura Y. [A G-protein gamma subunit mimic is a general antagonist of prion propagation in Saccharomyces cerevisiae.](http://www.ncbi.nlm.nih.gov/pubmed/19129493) *Proc Natl Acad Sci U S A.* 2009; **106:** 791-6.

Xue Y, Batlle M, Hirsch JP. [GPR1 encodes a putative G protein-coupled receptor that associates with the Gpa2p Galpha subunit and functions in a Ras-independent pathway.](http://www.ncbi.nlm.nih.gov/pubmed/9524122) *EMBO J.* 1998; **17:** 1996-2007.

Iyer RS, Das M, Bhat PJ. [Pseudohyphal differentiation defect due to mutations in GPCR and ammonium signaling is suppressed by low glucose concentration: a possible integrated role for carbon and nitrogen limitation.](http://www.ncbi.nlm.nih.gov/pubmed/18622617) *Curr Genet.*2008; **54:** 71-81.

Pan X, Heitman J. [Cyclic AMP-dependent protein kinase regulates pseudohyphal differentiation in Saccharomyces cerevisiae.](http://www.ncbi.nlm.nih.gov/pubmed/10373537) *Mol Cell Biol.* 1999; **19:** 4874-87.

Irniger S. [The Ime2 protein kinase family in fungi: more duties than just meiosis.](http://www.ncbi.nlm.nih.gov/pubmed/21306447) *Mol Microbiol.* 2011; **80:** 1-13.

Ruiz A, Liu Y, Xu X, Carlson M. [Heterotrimer-independent regulation of activation-loop phosphorylation of Snf1 protein kinase involves two protein phosphatases.](http://www.ncbi.nlm.nih.gov/pubmed/22589305) *Proc Natl Acad Sci U S A.* 2012; **109:** 8652-7.

Ludin K, Jiang R, Carlson M. [Glucose-regulated interaction of a regulatory subunit of protein phosphatase 1 with the Snf1 protein kinase in Saccharomyces cerevisiae.](http://www.ncbi.nlm.nih.gov/pubmed/9600950) *Proc Natl Acad Sci U S A.* 1998; **95:** 6245-50.

Gancedo JM. [Yeast carbon catabolite repression.](http://www.ncbi.nlm.nih.gov/pubmed/9618445) *Microbiol Mol Biol Rev.* 1998; **62:** 334-61.

Tabba S, Mangat S, McCartney R, Schmidt MC. [PP1 phosphatase-binding motif in Reg1 protein of Saccharomyces cerevisiae is required for interaction with both the PP1 phosphatase Glc7 and the Snf1 protein kinase.](http://www.ncbi.nlm.nih.gov/pubmed/20170726) *Cell Signal.*2010; **22:** 1013-21.

Schüller HJ. [Transcriptional control of nonfermentative metabolism in the yeast Saccharomyces cerevisiae.](http://www.ncbi.nlm.nih.gov/pubmed/12715202) *Curr Genet.*2003; **43:** 139-60.

Peláez R, Herrero P, Moreno F. [Functional domains of yeast hexokinase 2.](http://www.ncbi.nlm.nih.gov/pubmed/20815814) *Biochem J.* 2010; **432:** 181-90.

Ahuatzi D, Herrero P, de la Cera T, Moreno F. [The glucose-regulated nuclear localization of hexokinase 2 in Saccharomyces cerevisiae is Mig1-dependent.](http://www.ncbi.nlm.nih.gov/pubmed/14715653) *J Biol Chem.* 2004; **279:** 14440-6.

Ahuatzi D, Riera A, Peláez R, Herrero P, Moreno F. [Hxk2 regulates the phosphorylation state of Mig1 and therefore its nucleocytoplasmic distribution.](http://www.ncbi.nlm.nih.gov/pubmed/17178716) *J Biol Chem.* 2007; **282:** 4485-93.

Treitel MA, Carlson M. [Repression by SSN6-TUP1 is directed by MIG1, a repressor/activator protein.](http://www.ncbi.nlm.nih.gov/pubmed/7724528) *Proc Natl Acad Sci U S A.* 1995; **92:** 3132-6.

Song W, Carlson M. [Srb/mediator proteins interact functionally and physically with transcriptional repressor Sfl1.](http://www.ncbi.nlm.nih.gov/pubmed/9755175) *EMBO J.* 1998; **17:** 5757-65.

Treitel MA, Kuchin S, Carlson M. [Snf1 protein kinase regulates phosphorylation of the Mig1 repressor in Saccharomyces cerevisiae.](http://www.ncbi.nlm.nih.gov/pubmed/9774644) *Mol Cell Biol.* 1998; **18:** 6273-80.

Papamichos-Chronakis M, Gligoris T, Tzamarias D. [The Snf1 kinase controls glucose repression in yeast by modulating interactions between the Mig1 repressor and the Cyc8-Tup1 co-repressor.](http://www.ncbi.nlm.nih.gov/pubmed/15031717) *EMBO Rep.* 2004; **5:** 368-72.

Smith FC, Davies SP, Wilson WA, Carling D, Hardie DG. [The SNF1 kinase complex from Saccharomyces cerevisiae phosphorylates the transcriptional repressor protein Mig1p in vitro at four sites within or near regulatory domain 1.](http://www.ncbi.nlm.nih.gov/pubmed/10403407)*FEBS Lett.* 1999; **453:** 219-23.

Ozcan S, Johnston M. [Function and regulation of yeast hexose transporters.](http://www.ncbi.nlm.nih.gov/pubmed/10477308) *Microbiol Mol Biol Rev.* 1999; **63:** 554-69.

Boles E, Hollenberg CP. [The molecular genetics of hexose transport in yeasts.](http://www.ncbi.nlm.nih.gov/pubmed/9299703) *FEMS Microbiol Rev.* 1997; **21:** 85-111.

Maier A, Völker B, Boles E, Fuhrmann GF. [Characterisation of glucose transport in Saccharomyces cerevisiae with plasma membrane vesicles (countertransport) and intact cells (initial uptake) with single Hxt1, Hxt2, Hxt3, Hxt4, Hxt6, Hxt7 or Gal2 transporters.](http://www.ncbi.nlm.nih.gov/pubmed/12702270) *FEMS Yeast Res.* 2002; **2:** 539-50.

Tschopp JF, Emr SD, Field C, Schekman R. [GAL2 codes for a membrane-bound subunit of the galactose permease in Saccharomyces cerevisiae.](http://www.ncbi.nlm.nih.gov/pubmed/3082856) *J Bacteriol.* 1986; **166:** 313-8.

Harashima T, Anderson S, Yates JR 3rd, Heitman J. [The kelch proteins Gpb1 and Gpb2 inhibit Ras activity via association with the yeast RasGAP neurofibromin homologs Ira1 and Ira2.](http://www.ncbi.nlm.nih.gov/pubmed/16793550) *Mol Cell.* 2006; **22:** 819-30.

Phan VT, Ding VW, Li F, Chalkley RJ, Burlingame A, McCormick F. [The RasGAP proteins Ira2 and neurofibromin are negatively regulated by Gpb1 in yeast and ETEA in humans.](http://www.ncbi.nlm.nih.gov/pubmed/20160012) *Mol Cell Biol.* 2010; **30:** 2264-79.

Li Y, Wang Y. [Ras protein/cAMP-dependent protein kinase signaling is negatively regulated by a deubiquitinating enzyme, Ubp3, in yeast.](http://www.ncbi.nlm.nih.gov/pubmed/23476013) *J Biol Chem.* 2013; **288:** 11358-65.

Sakai A, Chibazakura T, Shimizu Y, Hishinuma F. [Molecular analysis of POP2 gene, a gene required for glucose-derepression of gene expression in Saccharomyces cerevisiae.](http://www.ncbi.nlm.nih.gov/pubmed/1475183) *Nucleic Acids Res.* 1992; **20:** 6227-33.

Ozcan S, Johnston M. [Two different repressors collaborate to restrict expression of the yeast glucose transporter genes HXT2 and HXT4 to low levels of glucose.](http://www.ncbi.nlm.nih.gov/pubmed/8816466) *Mol Cell Biol.* 1996; **16:** 5536-45.

DeVit MJ, Johnston M. [The nuclear exportin Msn5 is required for nuclear export of the Mig1 glucose repressor of Saccharomyces cerevisiae.](http://www.ncbi.nlm.nih.gov/pubmed/10556086) *Curr Biol.* 1999; **9:** 1231-41.

Moreno F, Herrero P. [The hexokinase 2-dependent glucose signal transduction pathway of Saccharomyces cerevisiae.](http://www.ncbi.nlm.nih.gov/pubmed/12007644)*FEMS Microbiol Rev.* 2002; **26:** 83-90.

Palomino A, Herrero P, Moreno F. [Rgt1, a glucose sensing transcription factor, is required for transcriptional repression of the HXK2 gene in Saccharomyces cerevisiae.](http://www.ncbi.nlm.nih.gov/pubmed/15705057) *Biochem J.* 2005; **388:** 697-703.

Ozcan S, Johnston M. [Three different regulatory mechanisms enable yeast hexose transporter (HXT) genes to be induced by different levels of glucose.](http://www.ncbi.nlm.nih.gov/pubmed/7862149) *Mol Cell Biol.* 1995; **15:** 1564-72.

Ozcan S, Leong T, Johnston M. [Rgt1p of Saccharomyces cerevisiae, a key regulator of glucose-induced genes, is both an activator and a repressor of transcription.](http://www.ncbi.nlm.nih.gov/pubmed/8887670) *Mol Cell Biol.* 1996; **16:** 6419-26.

Flick KM, Spielewoy N, Kalashnikova TI, Guaderrama M, Zhu Q, Chang HC, Wittenberg C. [Grr1-dependent inactivation of Mth1 mediates glucose-induced dissociation of Rgt1 from HXT gene promoters.](http://www.ncbi.nlm.nih.gov/pubmed/12925759) *Mol Biol Cell.* 2003; **14:** 3230-41.

Polish JA, Kim JH, Johnston M. [How the Rgt1 transcription factor of Saccharomyces cerevisiae is regulated by glucose.](http://www.ncbi.nlm.nih.gov/pubmed/15489524)*Genetics.* 2005; **169:** 583-94.

Soontorngun N, Baramee S, Tangsombatvichit C, Thepnok P, Cheevadhanarak S, Robert F, Turcotte B. [Genome-wide location analysis reveals an important overlap between the targets of the yeast transcriptional regulators Rds2 and Adr1.](http://www.ncbi.nlm.nih.gov/pubmed/22687600) *Biochem Biophys Res Commun.* 2012; **423:** 632-7.

van Oevelen CJ, van Teeffelen HA, van Werven FJ, Timmers HT. [Snf1p-dependent Spt-Ada-Gcn5-acetyltransferase (SAGA) recruitment and chromatin remodeling activities on the HXT2 and HXT4 promoters.](http://www.ncbi.nlm.nih.gov/pubmed/16368692) *J Biol Chem.* 2006; **281:**4523-31.

Lakshmanan J, Mosley AL, Ozcan S. [Repression of transcription by Rgt1 in the absence of glucose requires Std1 and Mth1.](http://www.ncbi.nlm.nih.gov/pubmed/14508605) *Curr Genet.* 2003; **44:** 19-25.

Schmidt MC, McCartney RR, Zhang X, Tillman TS, Solimeo H, Wölfl S, Almonte C, Watkins SC. [Std1 and Mth1 proteins interact with the glucose sensors to control glucose-regulated gene expression in Saccharomyces cerevisiae.](http://www.ncbi.nlm.nih.gov/pubmed/10373505) *Mol Cell Biol.* 1999; **19:** 4561-71.

Lafuente MJ, Gancedo C, Jauniaux JC, Gancedo JM. [Mth1 receives the signal given by the glucose sensors Snf3 and Rgt2 in Saccharomyces cerevisiae.](http://www.ncbi.nlm.nih.gov/pubmed/10632886) *Mol Microbiol.* 2000; **35:** 161-72.

Pasula S, Jouandot D 2nd, Kim JH. [Biochemical evidence for glucose-independent induction of HXT expression in Saccharomyces cerevisiae.](http://www.ncbi.nlm.nih.gov/pubmed/17586499) *FEBS Lett.* 2007; **581:** 3230-4.

Pasula S, Chakraborty S, Choi JH, Kim JH. [Role of casein kinase 1 in the glucose sensor-mediated signaling pathway in yeast.](http://www.ncbi.nlm.nih.gov/pubmed/20205947) *BMC Cell Biol.* 2010; **11:** 17.

Moriya H, Johnston M. [Glucose sensing and signaling in Saccharomyces cerevisiae through the Rgt2 glucose sensor and casein kinase I.](http://www.ncbi.nlm.nih.gov/pubmed/14755054) *Proc Natl Acad Sci U S A.* 2004; **101:** 1572-7.

Spielewoy N, Flick K, Kalashnikova TI, Walker JR, Wittenberg C. [Regulation and recognition of SCFGrr1 targets in the glucose and amino acid signaling pathways.](http://www.ncbi.nlm.nih.gov/pubmed/15456873) *Mol Cell Biol.* 2004; **24:** 8994-9005.

Purnapatre K, Gray M, Piccirillo S, Honigberg SM. [Glucose inhibits meiotic DNA replication through SCFGrr1p-dependent destruction of Ime2p kinase.](http://www.ncbi.nlm.nih.gov/pubmed/15601864) *Mol Cell Biol.* 2005; **25:** 440-50.

Mbonyi K, Beullens M, Detremerie K, Geerts L, Thevelein JM. [Requirement of one functional RAS gene and inability of an oncogenic ras variant to mediate the glucose-induced cyclic AMP signal in the yeast Saccharomyces cerevisiae.](http://www.ncbi.nlm.nih.gov/pubmed/2850478) *Mol Cell Biol.* 1988; **8:** 3051-7.

Kübler E, Mösch HU, Rupp S, Lisanti MP. [Gpa2p, a G-protein alpha-subunit, regulates growth and pseudohyphal development in Saccharomyces cerevisiae via a cAMP-dependent mechanism.](http://www.ncbi.nlm.nih.gov/pubmed/9252333) *J Biol Chem.* 1997; **272:** 20321-3.

Colombo S, Ma P, Cauwenberg L, Winderickx J, Crauwels M, Teunissen A, Nauwelaers D, de Winde JH, Gorwa MF, Colavizza D, Thevelein JM. [Involvement of distinct G-proteins, Gpa2 and Ras, in glucose- and intracellular acidification-induced cAMP signalling in the yeast Saccharomyces cerevisiae.](http://www.ncbi.nlm.nih.gov/pubmed/9628870) *EMBO J.* 1998; **17:** 3326-41.

Pascual-Ahuir A, Posas F, Serrano R, Proft M. [Multiple levels of control regulate the yeast cAMP-response element-binding protein repressor Sko1p in response to stress.](http://www.ncbi.nlm.nih.gov/pubmed/11500510) *J Biol Chem.* 2001; **276:** 37373-8.

Tomás-Cobos L, Casadomé L, Mas G, Sanz P, Posas F. [Expression of the HXT1 low affinity glucose transporter requires the coordinated activities of the HOG and glucose signalling pathways.](http://www.ncbi.nlm.nih.gov/pubmed/15014083) *J Biol Chem.* 2004; **279:** 22010-9.

Gross E, Goldberg D, Levitzki A. [Phosphorylation of the S. cerevisiae Cdc25 in response to glucose results in its dissociation from Ras.](http://www.ncbi.nlm.nih.gov/pubmed/1334534) *Nature.* 1992; **360:** 762-5.

Donzeau M, Bandlow W. [The yeast trimeric guanine nucleotide-binding protein alpha subunit, Gpa2p, controls the meiosis-specific kinase Ime2p activity in response to nutrients.](http://www.ncbi.nlm.nih.gov/pubmed/10454558) *Mol Cell Biol.* 1999; **19:** 6110-9.

Moriya H, Shimizu-Yoshida Y, Omori A, Iwashita S, Katoh M, Sakai A. [Yak1p, a DYRK family kinase, translocates to the nucleus and phosphorylates yeast Pop2p in response to a glucose signal.](http://www.ncbi.nlm.nih.gov/pubmed/11358866) *Genes Dev.* 2001; **15:** 1217-28.

Lee P, Cho BR, Joo HS, Hahn JS. [Yeast Yak1 kinase, a bridge between PKA and stress-responsive transcription factors, Hsf1 and Msn2/Msn4.](http://www.ncbi.nlm.nih.gov/pubmed/18793336) *Mol Microbiol.* 2008; **70:** 882-95.

Daugeron MC, Mauxion F, Séraphin B. [The yeast POP2 gene encodes a nuclease involved in mRNA deadenylation.](http://www.ncbi.nlm.nih.gov/pubmed/11410650) *Nucleic Acids Res.* 2001; **29:** 2448-55.

Crauwels M, Donaton MC, Pernambuco MB, Winderickx J, de Winde JH, Thevelein JM. [The Sch9 protein kinase in the yeast Saccharomyces cerevisiae controls cAPK activity and is required for nitrogen activation of the fermentable-growth-medium-induced (FGM) pathway.](http://www.ncbi.nlm.nih.gov/pubmed/9274016) *Microbiology.* 1997; **143 ( Pt 8):** 2627-37.

Cheng Q, Michels CA. [MAL11 and MAL61 encode the inducible high-affinity maltose transporter of Saccharomyces cerevisiae.](http://www.ncbi.nlm.nih.gov/pubmed/1999393) *J Bacteriol.* 1991; **173:** 1817-20.

Barrett L, Orlova M, Maziarz M, Kuchin S. [Protein kinase A contributes to the negative control of Snf1 protein kinase in Saccharomyces cerevisiae.](http://www.ncbi.nlm.nih.gov/pubmed/22140226) *Eukaryot Cell.* 2012; **11:** 119-28.

Vodermaier HC. [APC/C and SCF: controlling each other and the cell cycle.](http://www.ncbi.nlm.nih.gov/pubmed/15380093) *Curr Biol.* 2004; **14:** R787-96.

Versele M, de Winde JH, Thevelein JM. [A novel regulator of G protein signalling in yeast, Rgs2, downregulates glucose-activation of the cAMP pathway through direct inhibition of Gpa2.](http://www.ncbi.nlm.nih.gov/pubmed/10523302) *EMBO J.* 1999; **18:** 5577-91.

Urban J, Soulard A, Huber A, Lippman S, Mukhopadhyay D, Deloche O, Wanke V, Anrather D, Ammerer G, Riezman H, Broach JR, De Virgilio C, Hall MN, Loewith R. [Sch9 is a major target of TORC1 in Saccharomyces cerevisiae.](http://www.ncbi.nlm.nih.gov/pubmed/17560372) *Mol Cell.*2007; **26:** 663-74.

Welch AZ, Gibney PA, Botstein D, Koshland DE. [TOR and RAS pathways regulate desiccation tolerance in Saccharomyces cerevisiae.](http://www.ncbi.nlm.nih.gov/pubmed/23171550) *Mol Biol Cell.* 2013; **24:** 115-28.

Lempiäinen H, Uotila A, Urban J, Dohnal I, Ammerer G, Loewith R, Shore D. [Sfp1 interaction with TORC1 and Mrs6 reveals feedback regulation on TOR signaling.](http://www.ncbi.nlm.nih.gov/pubmed/19328065) *Mol Cell.* 2009; **33:** 704-16.

Moir RD, Lee J, Haeusler RA, Desai N, Engelke DR, Willis IM. [Protein kinase A regulates RNA polymerase III transcription through the nuclear localization of Maf1.](http://www.ncbi.nlm.nih.gov/pubmed/17005718) *Proc Natl Acad Sci U S A.* 2006; **103:** 15044-9.

Lee J, Moir RD, Willis IM. [Regulation of RNA polymerase III transcription involves SCH9-dependent and SCH9-independent branches of the target of rapamycin (TOR) pathway.](http://www.ncbi.nlm.nih.gov/pubmed/19299514) *J Biol Chem.* 2009; **284:** 12604-8.

Huber A, Bodenmiller B, Uotila A, Stahl M, Wanka S, Gerrits B, Aebersold R, Loewith R. [Characterization of the rapamycin-sensitive phosphoproteome reveals that Sch9 is a central coordinator of protein synthesis.](http://www.ncbi.nlm.nih.gov/pubmed/19684113) *Genes Dev.*2009; **23:** 1929-43.

Tate JJ, Georis I, Feller A, Dubois E, Cooper TG. [Rapamycin-induced Gln3 dephosphorylation is insufficient for nuclear localization: Sit4 and PP2A phosphatases are regulated and function differently.](http://www.ncbi.nlm.nih.gov/pubmed/19015262) *J Biol Chem.* 2009; **284:** 2522-34.

Oler AJ, Cairns BR. [PP4 dephosphorylates Maf1 to couple multiple stress conditions to RNA polymerase III repression.](http://www.ncbi.nlm.nih.gov/pubmed/22333918)*EMBO J.* 2012; **31:** 1440-52.

Towpik J, Graczyk D, Gajda A, Lefebvre O, Boguta M. [Derepression of RNA polymerase III transcription by phosphorylation and nuclear export of its negative regulator, Maf1.](http://www.ncbi.nlm.nih.gov/pubmed/18445601) *J Biol Chem.* 2008; **283:** 17168-74.

Lu JY, Lin YY, Sheu JC, Wu JT, Lee FJ, Chen Y, Lin MI, Chiang FT, Tai TY, Berger SL, Zhao Y, Tsai KS, Zhu H, Chuang LM, Boeke JD. [Acetylation of yeast AMPK controls intrinsic aging independently of caloric restriction.](http://www.ncbi.nlm.nih.gov/pubmed/21906795) *Cell.* 2011; **146:**969-79.

Malcher M, Schladebeck S, Mösch HU. [The Yak1 protein kinase lies at the center of a regulatory cascade affecting adhesive growth and stress resistance in Saccharomyces cerevisiae.](http://www.ncbi.nlm.nih.gov/pubmed/21149646) *Genetics.* 2011; **187:** 717-30.

Pan X, Heitman J. [Protein kinase A operates a molecular switch that governs yeast pseudohyphal differentiation.](http://www.ncbi.nlm.nih.gov/pubmed/12024012) *Mol Cell Biol.* 2002; **22:** 3981-93.

Tamaki H. [Glucose-stimulated cAMP-protein kinase A pathway in yeast Saccharomyces cerevisiae.](http://www.ncbi.nlm.nih.gov/pubmed/18023794) *J Biosci Bioeng.*2007; **104:** 245-50.

Gagiano M, Bauer FF, Pretorius IS. [The sensing of nutritional status and the relationship to filamentous growth in Saccharomyces cerevisiae.](http://www.ncbi.nlm.nih.gov/pubmed/12702263) *FEMS Yeast Res.* 2002; **2:** 433-70.

Lenssen E, Oberholzer U, Labarre J, De Virgilio C, Collart MA. [Saccharomyces cerevisiae Ccr4-not complex contributes to the control of Msn2p-dependent transcription by the Ras/cAMP pathway.](http://www.ncbi.nlm.nih.gov/pubmed/11929548) *Mol Microbiol.* 2002; **43:** 1023-37.

Young ET, Dombek KM, Tachibana C, Ideker T. [Multiple pathways are co-regulated by the protein kinase Snf1 and the transcription factors Adr1 and Cat8.](http://www.ncbi.nlm.nih.gov/pubmed/12676948) *J Biol Chem.* 2003; **278:** 26146-58.

Tachibana C, Yoo JY, Tagne JB, Kacherovsky N, Lee TI, Young ET. [Combined global localization analysis and transcriptome data identify genes that are directly coregulated by Adr1 and Cat8.](http://www.ncbi.nlm.nih.gov/pubmed/15743812) *Mol Cell Biol.* 2005; **25:** 2138-46.

Soontorngun N, Larochelle M, Drouin S, Robert F, Turcotte B. [Regulation of gluconeogenesis in Saccharomyces cerevisiae is mediated by activator and repressor functions of Rds2.](http://www.ncbi.nlm.nih.gov/pubmed/17875938) *Mol Cell Biol.* 2007; **27:** 7895-905.

Turcotte B, Liang XB, Robert F, Soontorngun N. [Transcriptional regulation of nonfermentable carbon utilization in budding yeast.](http://www.ncbi.nlm.nih.gov/pubmed/19686338) *FEMS Yeast Res.* 2010; **10:** 2-13.

Patel BA, Seiffert ER, Boyer DM, Jacobs RL, St Clair EM, Simons EL. [New primate first metatarsals from the Paleogene of Egypt and the origin of the anthropoid big toe.](http://www.ncbi.nlm.nih.gov/pubmed/22694838) *J Hum Evol.* 2012; **63:** 99-120.

Bermejo C, Haerizadeh F, Sadoine MS, Chermak D, Frommer WB. [Differential regulation of glucose transport activity in yeast by specific cAMP signatures.](http://www.ncbi.nlm.nih.gov/pubmed/23495665) *Biochem J.* 2013; **452:** 489-97.

Vincent O, Carlson M. [Gal83 mediates the interaction of the Snf1 kinase complex with the transcription activator Sip4.](http://www.ncbi.nlm.nih.gov/pubmed/10581241)*EMBO J.* 1999; **18:** 6672-81.

Charbon G, Breunig KD, Wattiez R, Vandenhaute J, Noël-Georis I. [Key role of Ser562/661 in Snf1-dependent regulation of Cat8p in Saccharomyces cerevisiae and Kluyveromyces lactis.](http://www.ncbi.nlm.nih.gov/pubmed/15121831) *Mol Cell Biol.* 2004; **24:** 4083-91.

Randez-Gil F, Bojunga N, Proft M, Entian KD. [Glucose derepression of gluconeogenic enzymes in Saccharomyces cerevisiae correlates with phosphorylation of the gene activator Cat8p.](http://www.ncbi.nlm.nih.gov/pubmed/9111319) *Mol Cell Biol.* 1997; **17:** 2502-10.

McNabb DS, Xing Y, Guarente L. [Cloning of yeast HAP5: a novel subunit of a heterotrimeric complex required for CCAAT binding.](http://www.ncbi.nlm.nih.gov/pubmed/7828851) *Genes Dev.* 1995; **9:** 47-58.

Dombek KM, Kacherovsky N, Young ET. [The Reg1-interacting proteins, Bmh1, Bmh2, Ssb1, and Ssb2, have roles in maintaining glucose repression in Saccharomyces cerevisiae.](http://www.ncbi.nlm.nih.gov/pubmed/15220335) *J Biol Chem.* 2004; **279:** 39165-74.

Baker SP, Grant PA. [The SAGA continues: expanding the cellular role of a transcriptional co-activator complex.](http://www.ncbi.nlm.nih.gov/pubmed/17694076)*Oncogene.* 2007; **26:** 5329-40.

Liu Y, Xu X, Kuo MH. [Snf1p regulates Gcn5p transcriptional activity by antagonizing Spt3p.](http://www.ncbi.nlm.nih.gov/pubmed/19841091) *Genetics.* 2010; **184:** 91-105.

Binda M, Péli-Gulli MP, Bonfils G, Panchaud N, Urban J, Sturgill TW, Loewith R, De Virgilio C. [The Vam6 GEF controls TORC1 by activating the EGO complex.](http://www.ncbi.nlm.nih.gov/pubmed/19748353) *Mol Cell.* 2009; **35:** 563-73.

Schmidt A, Beck T, Koller A, Kunz J, Hall MN. [The TOR nutrient signalling pathway phosphorylates NPR1 and inhibits turnover of the tryptophan permease.](http://www.ncbi.nlm.nih.gov/pubmed/9843498) *EMBO J.* 1998; **17:** 6924-31.

Jiang Y, Broach JR. [Tor proteins and protein phosphatase 2A reciprocally regulate Tap42 in controlling cell growth in yeast.](http://www.ncbi.nlm.nih.gov/pubmed/10329624) *EMBO J.* 1999; **18:** 2782-92.

MacGurn JA, Hsu PC, Smolka MB, Emr SD. [TORC1 regulates endocytosis via Npr1-mediated phosphoinhibition of a ubiquitin ligase adaptor.](http://www.ncbi.nlm.nih.gov/pubmed/22118465) *Cell.* 2011; **147:** 1104-17.

Merhi A, André B. [Internal amino acids promote Gap1 permease ubiquitylation via TORC1/Npr1/14-3-3-dependent control of the Bul arrestin-like adaptors.](http://www.ncbi.nlm.nih.gov/pubmed/22966204) *Mol Cell Biol.* 2012; **32:** 4510-22.

Jacinto E, Guo B, Arndt KT, Schmelzle T, Hall MN. [TIP41 interacts with TAP42 and negatively regulates the TOR signaling pathway.](http://www.ncbi.nlm.nih.gov/pubmed/11741537) *Mol Cell.* 2001; **8:** 1017-26.

González A, Ruiz A, Casamayor A, Ariño J. [Normal function of the yeast TOR pathway requires the type 2C protein phosphatase Ptc1.](http://www.ncbi.nlm.nih.gov/pubmed/19273591) *Mol Cell Biol.* 2009; **29:** 2876-88.

Yan G, Shen X, Jiang Y. [Rapamycin activates Tap42-associated phosphatases by abrogating their association with Tor complex 1.](http://www.ncbi.nlm.nih.gov/pubmed/16874307) *EMBO J.* 2006; **25:** 3546-55.

Di Como CJ, Arndt KT. [Nutrients, via the Tor proteins, stimulate the association of Tap42 with type 2A phosphatases.](http://www.ncbi.nlm.nih.gov/pubmed/8756348)*Genes Dev.* 1996; **10:** 1904-16.

Luke MM, Della Seta F, Di Como CJ, Sugimoto H, Kobayashi R, Arndt KT. [The SAP, a new family of proteins, associate and function positively with the SIT4 phosphatase.](http://www.ncbi.nlm.nih.gov/pubmed/8649382) *Mol Cell Biol.* 1996; **16:** 2744-55.

Inoki K, Ouyang H, Li Y, Guan KL. [Signaling by target of rapamycin proteins in cell growth control.](http://www.ncbi.nlm.nih.gov/pubmed/15755954) *Microbiol Mol Biol Rev.*2005; **69:** 79-100.

Düvel K, Santhanam A, Garrett S, Schneper L, Broach JR. [Multiple roles of Tap42 in mediating rapamycin-induced transcriptional changes in yeast.](http://www.ncbi.nlm.nih.gov/pubmed/12820961) *Mol Cell.* 2003; **11:** 1467-78.

Bertram PG, Choi JH, Carvalho J, Ai W, Zeng C, Chan TF, Zheng XF. [Tripartite regulation of Gln3p by TOR, Ure2p, and phosphatases.](http://www.ncbi.nlm.nih.gov/pubmed/10940301) *J Biol Chem.* 2000; **275:** 35727-33.

Crespo JL, Powers T, Fowler B, Hall MN. [The TOR-controlled transcription activators GLN3, RTG1, and RTG3 are regulated in response to intracellular levels of glutamine.](http://www.ncbi.nlm.nih.gov/pubmed/11997479) *Proc Natl Acad Sci U S A.* 2002; **99:** 6784-9.

Feller A, Boeckstaens M, Marini AM, Dubois E. [Transduction of the nitrogen signal activating Gln3-mediated transcription is independent of Npr1 kinase and Rsp5-Bul1/2 ubiquitin ligase in Saccharomyces cerevisiae.](http://www.ncbi.nlm.nih.gov/pubmed/16864574) *J Biol Chem.* 2006; **281:**28546-54.

Cardenas ME, Cutler NS, Lorenz MC, Di Como CJ, Heitman J. [The TOR signaling cascade regulates gene expression in response to nutrients.](http://www.ncbi.nlm.nih.gov/pubmed/10617575) *Genes Dev.* 1999; **13:** 3271-9.

Schmelzle T, Hall MN. [TOR, a central controller of cell growth.](http://www.ncbi.nlm.nih.gov/pubmed/11057898) *Cell.* 2000; **103:** 253-62.

Cunningham TS, Andhare R, Cooper TG. [Nitrogen catabolite repression of DAL80 expression depends on the relative levels of Gat1p and Ure2p production in Saccharomyces cerevisiae.](http://www.ncbi.nlm.nih.gov/pubmed/10799523) *J Biol Chem.* 2000; **275:** 14408-14.

Stanbrough M, Rowen DW, Magasanik B. [Role of the GATA factors Gln3p and Nil1p of Saccharomyces cerevisiae in the expression of nitrogen-regulated genes.](http://www.ncbi.nlm.nih.gov/pubmed/7568152) *Proc Natl Acad Sci U S A.* 1995; **92:** 9450-4.

Marini AM, Soussi-Boudekou S, Vissers S, Andre B. [A family of ammonium transporters in Saccharomyces cerevisiae.](http://www.ncbi.nlm.nih.gov/pubmed/9234685)*Mol Cell Biol.* 1997; **17:** 4282-93.

Dilova I, Aronova S, Chen JC, Powers T. [Tor signaling and nutrient-based signals converge on Mks1p phosphorylation to regulate expression of Rtg1.Rtg3p-dependent target genes.](http://www.ncbi.nlm.nih.gov/pubmed/15326168) *J Biol Chem.* 2004; **279:** 46527-35.

Dilova I, Chen CY, Powers T. [Mks1 in concert with TOR signaling negatively regulates RTG target gene expression in S. cerevisiae.](http://www.ncbi.nlm.nih.gov/pubmed/11882290) *Curr Biol.* 2002; **12:** 389-95.

Medvedik O, Lamming DW, Kim KD, Sinclair DA. [MSN2 and MSN4 link calorie restriction and TOR to sirtuin-mediated lifespan extension in Saccharomyces cerevisiae.](http://www.ncbi.nlm.nih.gov/pubmed/17914901) *PLoS Biol.* 2007; **5:** e261.

Anderson RM, Bitterman KJ, Wood JG, Medvedik O, Sinclair DA. [Nicotinamide and PNC1 govern lifespan extension by calorie restriction in Saccharomyces cerevisiae.](http://www.ncbi.nlm.nih.gov/pubmed/12736687) *Nature.* 2003; **423:** 181-5.

Huber A, French SL, Tekotte H, Yerlikaya S, Stahl M, Perepelkina MP, Tyers M, Rougemont J, Beyer AL, Loewith R. [Sch9 regulates ribosome biogenesis via Stb3, Dot6 and Tod6 and the histone deacetylase complex RPD3L.](http://www.ncbi.nlm.nih.gov/pubmed/21730963) *EMBO J.* 2011;**30:** 3052-64.

Marion RM, Regev A, Segal E, Barash Y, Koller D, Friedman N, O'Shea EK. [Sfp1 is a stress- and nutrient-sensitive regulator of ribosomal protein gene expression.](http://www.ncbi.nlm.nih.gov/pubmed/15353587) *Proc Natl Acad Sci U S A.* 2004; **101:** 14315-22.

Liko D, Conway MK, Grunwald DS, Heideman W. [Stb3 plays a role in the glucose-induced transition from quiescence to growth in Saccharomyces cerevisiae.](http://www.ncbi.nlm.nih.gov/pubmed/20385783) *Genetics.* 2010; **185:** 797-810.

Komeili A, Wedaman KP, O'Shea EK, Powers T. [Mechanism of metabolic control. Target of rapamycin signaling links nitrogen quality to the activity of the Rtg1 and Rtg3 transcription factors.](http://www.ncbi.nlm.nih.gov/pubmed/11076970) *J Cell Biol.* 2000; **151:** 863-78.

Sekito T, Thornton J, Butow RA. [Mitochondria-to-nuclear signaling is regulated by the subcellular localization of the transcription factors Rtg1p and Rtg3p.](http://www.ncbi.nlm.nih.gov/pubmed/10848632) *Mol Biol Cell.* 2000; **11:** 2103-15.

Dilova I, Powers T. [Accounting for strain-specific differences during RTG target gene regulation in Saccharomyces cerevisiae.](http://www.ncbi.nlm.nih.gov/pubmed/16423076) *FEMS Yeast Res.* 2006; **6:** 112-9.

Yan G, Lai Y, Jiang Y. [The TOR complex 1 is a direct target of Rho1 GTPase.](http://www.ncbi.nlm.nih.gov/pubmed/22445487) *Mol Cell.* 2012; **45:** 743-53.

Poulsen P, Lo Leggio L, Kielland-Brandt MC. [Mapping of an internal protease cleavage site in the Ssy5p component of the amino acid sensor of Saccharomyces cerevisiae and functional characterization of the resulting pro- and protease domains by gain-of-function genetics.](http://www.ncbi.nlm.nih.gov/pubmed/16524914) *Eukaryot Cell.* 2006; **5:** 601-8.

Klasson H, Fink GR, Ljungdahl PO. [Ssy1p and Ptr3p are plasma membrane components of a yeast system that senses extracellular amino acids.](http://www.ncbi.nlm.nih.gov/pubmed/10409731) *Mol Cell Biol.* 1999; **19:** 5405-16.

Bernard F, André B. [Ubiquitin and the SCF(Grr1) ubiquitin ligase complex are involved in the signalling pathway activated by external amino acids in Saccharomyces cerevisiae.](http://www.ncbi.nlm.nih.gov/pubmed/11356187) *FEBS Lett.* 2001; **496:** 81-5.

Eckert-Boulet N, Larsson K, Wu B, Poulsen P, Regenberg B, Nielsen J, Kielland-Brandt MC. [Deletion of RTS1, encoding a regulatory subunit of protein phosphatase 2A, results in constitutive amino acid signaling via increased Stp1p processing.](http://www.ncbi.nlm.nih.gov/pubmed/16400180) *Eukaryot Cell.* 2006; **5:** 174-9.

Liu Z, Thornton J, Spírek M, Butow RA. [Activation of the SPS amino acid-sensing pathway in Saccharomyces cerevisiae correlates with the phosphorylation state of a sensor component, Ptr3.](http://www.ncbi.nlm.nih.gov/pubmed/17984223) *Mol Cell Biol.* 2008; **28:** 551-63.

Poulsen P, Wu B, Gaber RF, Kielland-Brandt MC. [Constitutive signal transduction by mutant Ssy5p and Ptr3p components of the SPS amino acid sensor system in Saccharomyces cerevisiae.](http://www.ncbi.nlm.nih.gov/pubmed/15947203) *Eukaryot Cell.* 2005; **4:** 1116-24.

Abdel-Sater F, Iraqui I, Urrestarazu A, André B. [The external amino acid signaling pathway promotes activation of Stp1 and Uga35/Dal81 transcription factors for induction of the AGP1 gene in Saccharomyces cerevisiae.](http://www.ncbi.nlm.nih.gov/pubmed/15126393) *Genetics.* 2004;**166:** 1727-39.

Iraqui I, Vissers S, Bernard F, de Craene JO, Boles E, Urrestarazu A, André B. [Amino acid signaling in Saccharomyces cerevisiae: a permease-like sensor of external amino acids and F-Box protein Grr1p are required for transcriptional induction of the AGP1 gene, which encodes a broad-specificity amino acid permease.](http://www.ncbi.nlm.nih.gov/pubmed/9891035) *Mol Cell Biol.* 1999; **19:** 989-1001.

Boban M, Ljungdahl PO. [Dal81 enhances Stp1- and Stp2-dependent transcription necessitating negative modulation by inner nuclear membrane protein Asi1 in Saccharomyces cerevisiae.](http://www.ncbi.nlm.nih.gov/pubmed/17603098) *Genetics.* 2007; **176:** 2087-97.

Ljungdahl PO, Gimeno CJ, Styles CA, Fink GR. [SHR3: a novel component of the secretory pathway specifically required for localization of amino acid permeases in yeast.](http://www.ncbi.nlm.nih.gov/pubmed/1423607) *Cell.* 1992; **71:** 463-78.

Boban M, Zargari A, Andréasson C, Heessen S, Thyberg J, Ljungdahl PO. [Asi1 is an inner nuclear membrane protein that restricts promoter access of two latent transcription factors.](http://www.ncbi.nlm.nih.gov/pubmed/16735580) *J Cell Biol.* 2006; **173:** 695-707.

Zargari A, Boban M, Heessen S, Andréasson C, Thyberg J, Ljungdahl PO. [Inner nuclear membrane proteins Asi1, Asi2, and Asi3 function in concert to maintain the latent properties of transcription factors Stp1 and Stp2.](http://www.ncbi.nlm.nih.gov/pubmed/17085444) *J Biol Chem.* 2007;**282:** 594-605.

Regenberg B, Düring-Olsen L, Kielland-Brandt MC, Holmberg S. [Substrate specificity and gene expression of the amino-acid permeases in Saccharomyces cerevisiae.](http://www.ncbi.nlm.nih.gov/pubmed/10654085) *Curr Genet.* 1999; **36:** 317-28.

Jauniaux JC, Grenson M. [GAP1, the general amino acid permease gene of Saccharomyces cerevisiae. Nucleotide sequence, protein similarity with the other bakers yeast amino acid permeases, and nitrogen catabolite repression.](http://www.ncbi.nlm.nih.gov/pubmed/2194797) *Eur J Biochem.* 1990; **190:** 39-44.

Schmidt A, Hall MN, Koller A. [Two FK506 resistance-conferring genes in Saccharomyces cerevisiae, TAT1 and TAT2, encode amino acid permeases mediating tyrosine and tryptophan uptake.](http://www.ncbi.nlm.nih.gov/pubmed/7523855) *Mol Cell Biol.* 1994; **14:** 6597-606.

Didion T, Regenberg B, Jørgensen MU, Kielland-Brandt MC, Andersen HA. [The permease homologue Ssy1p controls the expression of amino acid and peptide transporter genes in Saccharomyces cerevisiae.](http://www.ncbi.nlm.nih.gov/pubmed/9489675) *Mol Microbiol.* 1998; **27:** 643-50.

Oficjalska-Pham D, Harismendy O, Smagowicz WJ, Gonzalez de Peredo A, Boguta M, Sentenac A, Lefebvre O. [General repression of RNA polymerase III transcription is triggered by protein phosphatase type 2A-mediated dephosphorylation of Maf1.](http://www.ncbi.nlm.nih.gov/pubmed/16762835) *Mol Cell.* 2006; **22:** 623-32.

Lorenz MC, Heitman J. [TOR mutations confer rapamycin resistance by preventing interaction with FKBP12-rapamycin.](http://www.ncbi.nlm.nih.gov/pubmed/7499212) *J Biol Chem.* 1995; **270:** 27531-7.

Limson MV, Sweder KS. [Rapamycin inhibits yeast nucleotide excision repair independently of tor kinases.](http://www.ncbi.nlm.nih.gov/pubmed/19805410) *Toxicol Sci.*2010; **113:** 77-84.

Hauser M, Narita V, Donhardt AM, Naider F, Becker JM. [Multiplicity and regulation of genes encoding peptide transporters in Saccharomyces cerevisiae.](http://www.ncbi.nlm.nih.gov/pubmed/11396605) *Mol Membr Biol.* 2001; **18:** 105-12.

De Craene JO, Soetens O, Andre B. [The Npr1 kinase controls biosynthetic and endocytic sorting of the yeast Gap1 permease.](http://www.ncbi.nlm.nih.gov/pubmed/11500493) *J Biol Chem.* 2001; **276:** 43939-48.

Beck T, Schmidt A, Hall MN. [Starvation induces vacuolar targeting and degradation of the tryptophan permease in yeast.](http://www.ncbi.nlm.nih.gov/pubmed/10491387)*J Cell Biol.* 1999; **146:** 1227-38.

Springael JY, André B. [Nitrogen-regulated ubiquitination of the Gap1 permease of Saccharomyces cerevisiae.](http://www.ncbi.nlm.nih.gov/pubmed/9614172) *Mol Biol Cell.* 1998; **9:** 1253-63.

Suzuki A, Mochizuki T, Uemura S, Hiraki T, Abe F. [Pressure-induced endocytic degradation of the Saccharomyces cerevisiae low-affinity tryptophan permease Tat1 is mediated by Rsp5 ubiquitin ligase and functionally redundant PPxY motif proteins.](http://www.ncbi.nlm.nih.gov/pubmed/23666621) *Eukaryot Cell.* 2013; **12:** 990-7.

Day RE, Rogers PJ, Dawes IW, Higgins VJ. [Molecular analysis of maltotriose transport and utilization by Saccharomyces cerevisiae.](http://www.ncbi.nlm.nih.gov/pubmed/12406721) *Appl Environ Microbiol.* 2002; **68:** 5326-35.

Palomino A, Herrero P, Moreno F. [Tpk3 and Snf1 protein kinases regulate Rgt1 association with Saccharomyces cerevisiae HXK2 promoter.](http://www.ncbi.nlm.nih.gov/pubmed/16528100) *Nucleic Acids Res.* 2006; **34:** 1427-38.

Boisnard S, Lagniel G, Garmendia-Torres C, Molin M, Boy-Marcotte E, Jacquet M, Toledano MB, Labarre J, Chédin S.[H2O2 activates the nuclear localization of Msn2 and Maf1 through thioredoxins in Saccharomyces cerevisiae.](http://www.ncbi.nlm.nih.gov/pubmed/19581440)*Eukaryot Cell.* 2009; **8:** 1429-38.

Guttmann-Raviv N, Martin S, Kassir Y. [Ime2, a meiosis-specific kinase in yeast, is required for destabilization of its transcriptional activator, Ime1.](http://www.ncbi.nlm.nih.gov/pubmed/11884593) *Mol Cell Biol.* 2002; **22:** 2047-56.

Bourot S, Karst F. [Isolation and characterization of the Saccharomyces cerevisiae SUT1 gene involved in sterol uptake.](http://www.ncbi.nlm.nih.gov/pubmed/7489925)*Gene.* 1995; **165:** 97-102.

Collart MA, Panasenko OO. [The Ccr4--not complex.](http://www.ncbi.nlm.nih.gov/pubmed/22027279) *Gene.* 2012; **492:** 42-53.

Taussig R, Carlson M. [Nucleotide sequence of the yeast SUC2 gene for invertase.](http://www.ncbi.nlm.nih.gov/pubmed/6300785) *Nucleic Acids Res.* 1983; **11:** 1943-54.

Hitzeman RA, Clarke L, Carbon J. [Isolation and characterization of the yeast 3-phosphoglycerokinase gene (PGK) by an immunological screening technique.](http://www.ncbi.nlm.nih.gov/pubmed/6254992) *J Biol Chem.* 1980; **255:** 12073-80.

Marini AM, Vissers S, Urrestarazu A, André B. [Cloning and expression of the MEP1 gene encoding an ammonium transporter in Saccharomyces cerevisiae.](http://www.ncbi.nlm.nih.gov/pubmed/8062822) *EMBO J.* 1994; **13:** 3456-63.

Benjamin PM, Wu JI, Mitchell AP, Magasanik B. [Three regulatory systems control expression of glutamine synthetase in Saccharomyces cerevisiae at the level of transcription.](http://www.ncbi.nlm.nih.gov/pubmed/2570348) *Mol Gen Genet.* 1989; **217:** 370-7.

Miller SM, Magasanik B. [Role of NAD-linked glutamate dehydrogenase in nitrogen metabolism in Saccharomyces cerevisiae.](http://www.ncbi.nlm.nih.gov/pubmed/1975578) *J Bacteriol.* 1990; **172:** 4927-35.

Aguilera J, Randez-Gil F, Prieto JA. [Cold response in Saccharomyces cerevisiae: new functions for old mechanisms.](http://www.ncbi.nlm.nih.gov/pubmed/17298585)*FEMS Microbiol Rev.* 2007; **31:** 327-41.

Nakagawa Y, Sakumoto N, Kaneko Y, Harashima S. [Mga2p is a putative sensor for low temperature and oxygen to induce OLE1 transcription in Saccharomyces cerevisiae.](http://www.ncbi.nlm.nih.gov/pubmed/11855848) *Biochem Biophys Res Commun.* 2002; **291:** 707-13.

Hayashi M, Maeda T. [Activation of the HOG pathway upon cold stress in Saccharomyces cerevisiae.](http://www.ncbi.nlm.nih.gov/pubmed/16672281) *J Biochem.* 2006;**139:** 797-803.

Panadero J, Pallotti C, Rodríguez-Vargas S, Randez-Gil F, Prieto JA. [A downshift in temperature activates the high osmolarity glycerol (HOG) pathway, which determines freeze tolerance in Saccharomyces cerevisiae.](http://www.ncbi.nlm.nih.gov/pubmed/16371351) *J Biol Chem.*2006; **281:** 4638-45.

Al-Fageeh MB, Smales CM. [Control and regulation of the cellular responses to cold shock: the responses in yeast and mammalian systems.](http://www.ncbi.nlm.nih.gov/pubmed/16792527) *Biochem J.* 2006; **397:** 247-59.

Homma T, Iwahashi H, Komatsu Y. [Yeast gene expression during growth at low temperature.](http://www.ncbi.nlm.nih.gov/pubmed/12818212) *Cryobiology.* 2003; **46:** 230-7.

Posas F, Wurgler-Murphy SM, Maeda T, Witten EA, Thai TC, Saito H. [Yeast HOG1 MAP kinase cascade is regulated by a multistep phosphorelay mechanism in the SLN1-YPD1-SSK1 "two-component" osmosensor.](http://www.ncbi.nlm.nih.gov/pubmed/8808622) *Cell.* 1996; **86:** 865-75.

Yamamoto K, Tatebayashi K, Tanaka K, Saito H. [Dynamic control of yeast MAP kinase network by induced association and dissociation between the Ste50 scaffold and the Opy2 membrane anchor.](http://www.ncbi.nlm.nih.gov/pubmed/20932477) *Mol Cell.* 2010; **40:** 87-98.

Saito H, Tatebayashi K. [Regulation of the osmoregulatory HOG MAPK cascade in yeast.](http://www.ncbi.nlm.nih.gov/pubmed/15598881) *J Biochem.* 2004; **136:** 267-72.

Reiser V, Ruis H, Ammerer G. [Kinase activity-dependent nuclear export opposes stress-induced nuclear accumulation and retention of Hog1 mitogen-activated protein kinase in the budding yeast Saccharomyces cerevisiae.](http://www.ncbi.nlm.nih.gov/pubmed/10198063) *Mol Biol Cell.*1999; **10:** 1147-61.

Pascual-Ahuir A, Proft M. [The Sch9 kinase is a chromatin-associated transcriptional activator of osmostress-responsive genes.](http://www.ncbi.nlm.nih.gov/pubmed/17568771) *EMBO J.* 2007; **26:** 3098-108.

Proft M, Struhl K. [Hog1 kinase converts the Sko1-Cyc8-Tup1 repressor complex into an activator that recruits SAGA and SWI/SNF in response to osmotic stress.](http://www.ncbi.nlm.nih.gov/pubmed/12086627) *Mol Cell.* 2002; **9:** 1307-17.

Rios G, Ferrando A, Serrano R. [Mechanisms of salt tolerance conferred by overexpression of the HAL1 gene in Saccharomyces cerevisiae.](http://www.ncbi.nlm.nih.gov/pubmed/9178503) *Yeast.* 1997; **13:** 515-28.

Pascual-Ahuir A, Serrano R, Proft M. [The Sko1p repressor and Gcn4p activator antagonistically modulate stress-regulated transcription in Saccharomyces cerevisiae.](http://www.ncbi.nlm.nih.gov/pubmed/11113177) *Mol Cell Biol.* 2001; **21:** 16-25.

Proft M, Serrano R. [Repressors and upstream repressing sequences of the stress-regulated ENA1 gene in Saccharomyces cerevisiae: bZIP protein Sko1p confers HOG-dependent osmotic regulation.](http://www.ncbi.nlm.nih.gov/pubmed/9858577) *Mol Cell Biol.* 1999; **19:**537-46.

Bermejo C, Rodríguez E, García R, Rodríguez-Peña JM, Rodríguez de la Concepción ML, Rivas C, Arias P, Nombela C, Posas F, Arroyo J. [The sequential activation of the yeast HOG and SLT2 pathways is required for cell survival to cell wall stress.](http://www.ncbi.nlm.nih.gov/pubmed/18184748) *Mol Biol Cell.* 2008; **19:** 1113-24.

García R, Rodríguez-Peña JM, Bermejo C, Nombela C, Arroyo J. [The high osmotic response and cell wall integrity pathways cooperate to regulate transcriptional responses to zymolyase-induced cell wall stress in Saccharomyces cerevisiae.](http://www.ncbi.nlm.nih.gov/pubmed/19234305) *J Biol Chem.* 2009; **284:** 10901-11.

Pedrajas JR, Kosmidou E, Miranda-Vizuete A, Gustafsson JA, Wright AP, Spyrou G. [Identification and functional characterization of a novel mitochondrial thioredoxin system in Saccharomyces cerevisiae.](http://www.ncbi.nlm.nih.gov/pubmed/10037727) *J Biol Chem.* 1999; **274:**6366-73.

Posas F, Witten EA, Saito H. [Requirement of STE50 for osmostress-induced activation of the STE11 mitogen-activated protein kinase kinase kinase in the high-osmolarity glycerol response pathway.](http://www.ncbi.nlm.nih.gov/pubmed/9742096) *Mol Cell Biol.* 1998; **18:** 5788-96.

Wu C, Leberer E, Thomas DY, Whiteway M. [Functional characterization of the interaction of Ste50p with Ste11p MAPKKK in Saccharomyces cerevisiae.](http://www.ncbi.nlm.nih.gov/pubmed/10397774) *Mol Biol Cell.* 1999; **10:** 2425-40.

Lee EJ, Choi J, Groisman EA. [Control of a Salmonella virulence operon by proline-charged tRNA(Pro).](http://www.ncbi.nlm.nih.gov/pubmed/24516160) *Proc Natl Acad Sci U S A.* 2014; **111:** 3140-5.

Tamás MJ, Rep M, Thevelein JM, Hohmann S. [Stimulation of the yeast high osmolarity glycerol (HOG) pathway: evidence for a signal generated by a change in turgor rather than by water stress.](http://www.ncbi.nlm.nih.gov/pubmed/10781825) *FEBS Lett.* 2000; **472:** 159-65.

Li S, Ault A, Malone CL, Raitt D, Dean S, Johnston LH, Deschenes RJ, Fassler JS. [The yeast histidine protein kinase, Sln1p, mediates phosphotransfer to two response regulators, Ssk1p and Skn7p.](http://www.ncbi.nlm.nih.gov/pubmed/9843501) *EMBO J.* 1998; **17:** 6952-62.

Tamás MJ, Luyten K, Sutherland FC, Hernandez A, Albertyn J, Valadi H, Li H, Prior BA, Kilian SG, Ramos J, Gustafsson L, Thevelein JM, Hohmann S. [Fps1p controls the accumulation and release of the compatible solute glycerol in yeast osmoregulation.](http://www.ncbi.nlm.nih.gov/pubmed/10096077) *Mol Microbiol.* 1999; **31:** 1087-104.

Pettersson N, Filipsson C, Becit E, Brive L, Hohmann S. [Aquaporins in yeasts and filamentous fungi.](http://www.ncbi.nlm.nih.gov/pubmed/15966864) *Biol Cell.* 2005; **97:**487-500.

Oliveira R, Lages F, Silva-Graça M, Lucas C. [Fps1p channel is the mediator of the major part of glycerol passive diffusion in Saccharomyces cerevisiae: artefacts and re-definitions.](http://www.ncbi.nlm.nih.gov/pubmed/12832087) *Biochim Biophys Acta.* 2003; **1613:** 57-71.

Beese SE, Negishi T, Levin DE. [Identification of positive regulators of the yeast fps1 glycerol channel.](http://www.ncbi.nlm.nih.gov/pubmed/19956799) *PLoS Genet.* 2009;**5:** e1000738.

Compagno C, Brambilla L, Capitanio D, Boschi F, Ranzi BM, Porro D. [Alterations of the glucose metabolism in a triose phosphate isomerase-negative Saccharomyces cerevisiae mutant.](http://www.ncbi.nlm.nih.gov/pubmed/11329176) *Yeast.* 2001; **18:** 663-70.

Alber T, Kawasaki G. [Nucleotide sequence of the triose phosphate isomerase gene of Saccharomyces cerevisiae.](http://www.ncbi.nlm.nih.gov/pubmed/6759603) *J Mol Appl Genet.* 1982; **1:** 419-34.

Aguilera A, Zimmermann FK. [Isolation and molecular analysis of the phosphoglucose isomerase structural gene of Saccharomyces cerevisiae.](http://www.ncbi.nlm.nih.gov/pubmed/3007940) *Mol Gen Genet.* 1986; **202:** 83-9.

Lowe SL, Reithel FJ. [The subunit structure of phosphoglucose isomerase from bakers' yeast.](http://www.ncbi.nlm.nih.gov/pubmed/1095569) *J Biol Chem.* 1975; **250:**94-9.

Maitra PK, Lobo Z. [Genetic studies with a phosphoglucose isomerase mutant of Saccharomyces cerevisiae.](http://www.ncbi.nlm.nih.gov/pubmed/340892) *Mol Gen Genet.* 1977; **156:** 55-60.

Heinisch J. [Isolation and characterization of the two structural genes coding for phosphofructokinase in yeast.](http://www.ncbi.nlm.nih.gov/pubmed/3007939) *Mol Gen Genet.* 1986; **202:** 75-82.

Clifton D, Fraenkel DG. [Mutant studies of yeast phosphofructokinase.](http://www.ncbi.nlm.nih.gov/pubmed/6211191) *Biochemistry.* 1982; **21:** 1935-42.

Mercado JJ, Gancedo JM. [Regulatory regions in the yeast FBP1 and PCK1 genes.](http://www.ncbi.nlm.nih.gov/pubmed/1327878) *FEBS Lett.* 1992; **311:** 110-4.

Walsh RB, Kawasaki G, Fraenkel DG. [Cloning of genes that complement yeast hexokinase and glucokinase mutants.](http://www.ncbi.nlm.nih.gov/pubmed/6341351) *J Bacteriol.* 1983; **154:** 1002-4.

Clifton D, Walsh RB, Fraenkel DG. [Functional studies of yeast glucokinase.](http://www.ncbi.nlm.nih.gov/pubmed/8501032) *J Bacteriol.* 1993; **175:** 3289-94.

Bianconi ML. [Calorimetric determination of thermodynamic parameters of reaction reveals different enthalpic compensations of the yeast hexokinase isozymes.](http://www.ncbi.nlm.nih.gov/pubmed/12611889) *J Biol Chem.* 2003; **278:** 18709-13.

Norbeck J, Pâhlman AK, Akhtar N, Blomberg A, Adler L. [Purification and characterization of two isoenzymes of DL-glycerol-3-phosphatase from Saccharomyces cerevisiae. Identification of the corresponding GPP1 and GPP2 genes and evidence for osmotic regulation of Gpp2p expression by the osmosensing mitogen-activated protein kinase signal transduction pathway.](http://www.ncbi.nlm.nih.gov/pubmed/8662716) *J Biol Chem.* 1996; **271:** 13875-81.

Costenoble R, Valadi H, Gustafsson L, Niklasson C, Franzén CJ. [Microaerobic glycerol formation in Saccharomyces cerevisiae.](http://www.ncbi.nlm.nih.gov/pubmed/11113971) *Yeast.* 2000; **16:** 1483-95.

Kretschmer M, Fraenkel DG. [Yeast 6-phosphofructo-2-kinase: sequence and mutant.](http://www.ncbi.nlm.nih.gov/pubmed/1657152) *Biochemistry.* 1991; **30:** 10663-72.

Boles E, Göhlmann HW, Zimmermann FK. [Cloning of a second gene encoding 5-phosphofructo-2-kinase in yeast, and characterization of mutant strains without fructose-2,6-bisphosphate.](http://www.ncbi.nlm.nih.gov/pubmed/8861205) *Mol Microbiol.* 1996; **20:** 65-76.

Dihazi H, Kessler R, Eschrich K. [High osmolarity glycerol (HOG) pathway-induced phosphorylation and activation of 6-phosphofructo-2-kinase are essential for glycerol accumulation and yeast cell proliferation under hyperosmotic stress.](http://www.ncbi.nlm.nih.gov/pubmed/15037628)*J Biol Chem.* 2004; **279:** 23961-8.

Jung JY, Kim TY, Ng CY, Oh MK. [Characterization of GCY1 in Saccharomyces cerevisiae by metabolic profiling.](http://www.ncbi.nlm.nih.gov/pubmed/22979944) *J Appl Microbiol.* 2012; **113:** 1468-78.

Ford G, Ellis EM. [Characterization of Ypr1p from Saccharomyces cerevisiae as a 2-methylbutyraldehyde reductase.](http://www.ncbi.nlm.nih.gov/pubmed/12210903)*Yeast.* 2002; **19:** 1087-96.

Molin M, Norbeck J, Blomberg A. [Dihydroxyacetone kinases in Saccharomyces cerevisiae are involved in detoxification of dihydroxyacetone.](http://www.ncbi.nlm.nih.gov/pubmed/12401799) *J Biol Chem.* 2003; **278:** 1415-23.

Oliveira R, Lucas C. [Expression studies of GUP1 and GUP2, genes involved in glycerol active transport in Saccharomyces cerevisiae, using semi-quantitative RT-PCR.](http://www.ncbi.nlm.nih.gov/pubmed/15278288) *Curr Genet.* 2004; **46:** 140-6.

Lages F, Lucas C. [Contribution to the physiological characterization of glycerol active uptake in Saccharomyces cerevisiae.](http://www.ncbi.nlm.nih.gov/pubmed/9398075) *Biochim Biophys Acta.* 1997; **1322:** 8-18.

Holst B, Lunde C, Lages F, Oliveira R, Lucas C, Kielland-Brandt MC. [GUP1 and its close homologue GUP2, encoding multimembrane-spanning proteins involved in active glycerol uptake in Saccharomyces cerevisiae.](http://www.ncbi.nlm.nih.gov/pubmed/10931309) *Mol Microbiol.* 2000;**37:** 108-24.

Ferreira C, van Voorst F, Martins A, Neves L, Oliveira R, Kielland-Brandt MC, Lucas C, Brandt A. [A member of the sugar transporter family, Stl1p is the glycerol/H+ symporter in Saccharomyces cerevisiae.](http://www.ncbi.nlm.nih.gov/pubmed/15703210) *Mol Biol Cell.* 2005; **16:** 2068-76.

Furukawa K, Sidoux-Walter F, Hohmann S. [Expression of the yeast aquaporin Aqy2 affects cell surface properties under the control of osmoregulatory and morphogenic signalling pathways.](http://www.ncbi.nlm.nih.gov/pubmed/19889095) *Mol Microbiol.* 2009; **74:** 1272-86.

Bonhivers M, Carbrey JM, Gould SJ, Agre P. [Aquaporins in Saccharomyces. Genetic and functional distinctions between laboratory and wild-type strains.](http://www.ncbi.nlm.nih.gov/pubmed/9765289) *J Biol Chem.* 1998; **273:** 27565-72.

Carbrey JM, Bonhivers M, Boeke JD, Agre P. [Aquaporins in Saccharomyces: Characterization of a second functional water channel protein.](http://www.ncbi.nlm.nih.gov/pubmed/11158584) *Proc Natl Acad Sci U S A.* 2001; **98:** 1000-5.

Luyten K, Albertyn J, Skibbe WF, Prior BA, Ramos J, Thevelein JM, Hohmann S. [Fps1, a yeast member of the MIP family of channel proteins, is a facilitator for glycerol uptake and efflux and is inactive under osmotic stress.](http://www.ncbi.nlm.nih.gov/pubmed/7729414) *EMBO J.* 1995;**14:** 1360-71.

MARSCHAK J. [ECONOMICS OF LANGUAGE.](http://www.ncbi.nlm.nih.gov/pubmed/14284288) *Behav Sci.* 1965; **10:** 135-40.

Lommel M, Bagnat M, Strahl S. [Aberrant processing of the WSC family and Mid2p cell surface sensors results in cell death of Saccharomyces cerevisiae O-mannosylation mutants.](http://www.ncbi.nlm.nih.gov/pubmed/14673142) *Mol Cell Biol.* 2004; **24:** 46-57.

Mil'chenko KP, Afonskaia SV. [[Action of some polysaccharide containing complexes of microbial origin on the tobacco mosaic virus and on the influenza virus].](http://www.ncbi.nlm.nih.gov/pubmed/948273) *Mikrobiol Zh.* 1976; **38:** 347-50.

Zhang S, Skalsky Y, Garfinkel DJ. [MGA2 or SPT23 is required for transcription of the delta9 fatty acid desaturase gene, OLE1, and nuclear membrane integrity in Saccharomyces cerevisiae.](http://www.ncbi.nlm.nih.gov/pubmed/9927444) *Genetics.* 1999; **151:** 473-83.

Abramova NE, Cohen BD, Sertil O, Kapoor R, Davies KJ, Lowry CV. [Regulatory mechanisms controlling expression of the DAN/TIR mannoprotein genes during anaerobic remodeling of the cell wall in Saccharomyces cerevisiae.](http://www.ncbi.nlm.nih.gov/pubmed/11238402) *Genetics.*2001; **157:** 1169-77.

Abramova N, Sertil O, Mehta S, Lowry CV. [Reciprocal regulation of anaerobic and aerobic cell wall mannoprotein gene expression in Saccharomyces cerevisiae.](http://www.ncbi.nlm.nih.gov/pubmed/11292809) *J Bacteriol.* 2001; **183:** 2881-7.

Jiang Y, Vasconcelles MJ, Wretzel S, Light A, Martin CE, Goldberg MA. [MGA2 is involved in the low-oxygen response element-dependent hypoxic induction of genes in Saccharomyces cerevisiae.](http://www.ncbi.nlm.nih.gov/pubmed/11509659) *Mol Cell Biol.* 2001; **21:** 6161-9.

Kondo K, Inouye M. [TIP 1, a cold shock-inducible gene of Saccharomyces cerevisiae.](http://www.ncbi.nlm.nih.gov/pubmed/1894636) *J Biol Chem.* 1991; **266:** 17537-44.

Kowalski LR, Kondo K, Inouye M. [Cold-shock induction of a family of TIP1-related proteins associated with the membrane in Saccharomyces cerevisiae.](http://www.ncbi.nlm.nih.gov/pubmed/7746155) *Mol Microbiol.* 1995; **15:** 341-53.

Muñoz-Dorado J, Kondo K, Inouye M, Sone H. [Identification of cis- and trans-acting elements involved in the expression of cold shock-inducible TIP1 gene of yeast Saccharomyces cerevisiae.](http://www.ncbi.nlm.nih.gov/pubmed/8127704) *Nucleic Acids Res.* 1994; **22:** 560-8.

Murata Y, Watanabe T, Sato M, Momose Y, Nakahara T, Oka S, Iwahashi H. [Dimethyl sulfoxide exposure facilitates phospholipid biosynthesis and cellular membrane proliferation in yeast cells.](http://www.ncbi.nlm.nih.gov/pubmed/12771156) *J Biol Chem.* 2003; **278:** 33185-93.

Tatebayashi K, Yamamoto K, Tanaka K, Tomida T, Maruoka T, Kasukawa E, Saito H. [Adaptor functions of Cdc42, Ste50, and Sho1 in the yeast osmoregulatory HOG MAPK pathway.](http://www.ncbi.nlm.nih.gov/pubmed/16778768) *EMBO J.* 2006; **25:** 3033-44.

Pavlik P, Simon M, Schuster T, Ruis H. [The glycerol kinase (GUT1) gene of Saccharomyces cerevisiae: cloning and characterization.](http://www.ncbi.nlm.nih.gov/pubmed/8358828) *Curr Genet.* 1993; **24:** 21-5.

Rønnow B, Kielland-Brandt MC. [GUT2, a gene for mitochondrial glycerol 3-phosphate dehydrogenase of Saccharomyces cerevisiae.](http://www.ncbi.nlm.nih.gov/pubmed/8256521) *Yeast.* 1993; **9:** 1121-30.

Lobo Z. [Saccharomyces cerevisiae aldolase mutants.](http://www.ncbi.nlm.nih.gov/pubmed/6384192) *J Bacteriol.* 1984; **160:** 222-6.

Cutler NS, Heitman J, Cardenas ME. [STT4 is an essential phosphatidylinositol 4-kinase that is a target of wortmannin in Saccharomyces cerevisiae.](http://www.ncbi.nlm.nih.gov/pubmed/9346907) *J Biol Chem.* 1997; **272:** 27671-7.

Desrivières S, Cooke FT, Parker PJ, Hall MN. [MSS4, a phosphatidylinositol-4-phosphate 5-kinase required for organization of the actin cytoskeleton in Saccharomyces cerevisiae.](http://www.ncbi.nlm.nih.gov/pubmed/9624178) *J Biol Chem.* 1998; **273:** 15787-93.

Homma K, Terui S, Minemura M, Qadota H, Anraku Y, Kanaho Y, Ohya Y. [Phosphatidylinositol-4-phosphate 5-kinase localized on the plasma membrane is essential for yeast cell morphogenesis.](http://www.ncbi.nlm.nih.gov/pubmed/9624177) *J Biol Chem.* 1998; **273:** 15779-86.

Yorimitsu T, He C, Wang K, Klionsky DJ. [Tap42-associated protein phosphatase type 2A negatively regulates induction of autophagy.](http://www.ncbi.nlm.nih.gov/pubmed/19223769) *Autophagy.* 2009; **5:** 616-24.

Greetham D, Grant CM. [Antioxidant activity of the yeast mitochondrial one-Cys peroxiredoxin is dependent on thioredoxin reductase and glutathione in vivo.](http://www.ncbi.nlm.nih.gov/pubmed/19332553) *Mol Cell Biol.* 2009; **29:** 3229-40.

Pedrajas JR, Padilla CA, McDonagh B, Bárcena JA. [Glutaredoxin participates in the reduction of peroxides by the mitochondrial 1-CYS peroxiredoxin in Saccharomyces cerevisiae.](http://www.ncbi.nlm.nih.gov/pubmed/20059400) *Antioxid Redox Signal.* 2010; **13:** 249-58.

Tan SX, Greetham D, Raeth S, Grant CM, Dawes IW, Perrone GG. [The thioredoxin-thioredoxin reductase system can function in vivo as an alternative system to reduce oxidized glutathione in Saccharomyces cerevisiae.](http://www.ncbi.nlm.nih.gov/pubmed/19951944) *J Biol Chem.*2010; **285:** 6118-26.

Izawa S, Inoue Y, Kimura A. [Importance of catalase in the adaptive response to hydrogen peroxide: analysis of acatalasaemic Saccharomyces cerevisiae.](http://www.ncbi.nlm.nih.gov/pubmed/8947468) *Biochem J.* 1996; **320 ( Pt 1):** 61-7.

Lewis DA, Bisson LF. [The HXT1 gene product of Saccharomyces cerevisiae is a new member of the family of hexose transporters.](http://www.ncbi.nlm.nih.gov/pubmed/2046678) *Mol Cell Biol.* 1991; **11:** 3804-13.

Chen CN, Porubleva L, Shearer G, Svrakic M, Holden LG, Dover JL, Johnston M, Chitnis PR, Kohl DH. [Associating protein activities with their genes: rapid identification of a gene encoding a methylglyoxal reductase in the yeast Saccharomyces cerevisiae.](http://www.ncbi.nlm.nih.gov/pubmed/12722185) *Yeast.* 2003; **20:** 545-54.

Fahrenkrog B, Sauder U, Aebi U. [The S. cerevisiae HtrA-like protein Nma111p is a nuclear serine protease that mediates yeast apoptosis.](http://www.ncbi.nlm.nih.gov/pubmed/14657274) *J Cell Sci.* 2004; **117:** 115-26.

Errede B, Gartner A, Zhou Z, Nasmyth K, Ammerer G. [MAP kinase-related FUS3 from S. cerevisiae is activated by STE7 in vitro.](http://www.ncbi.nlm.nih.gov/pubmed/8384702) *Nature.* 1993; **362:** 261-4.

Zhou Z, Gartner A, Cade R, Ammerer G, Errede B. [Pheromone-induced signal transduction in Saccharomyces cerevisiae requires the sequential function of three protein kinases.](http://www.ncbi.nlm.nih.gov/pubmed/8455599) *Mol Cell Biol.* 1993; **13:** 2069-80.

Elion EA, Brill JA, Fink GR. [Functional redundancy in the yeast cell cycle: FUS3 and KSS1 have both overlapping and unique functions.](http://www.ncbi.nlm.nih.gov/pubmed/1819502) *Cold Spring Harb Symp Quant Biol.* 1991; **56:** 41-9.

Stukey JE, McDonough VM, Martin CE. [The OLE1 gene of Saccharomyces cerevisiae encodes the delta 9 fatty acid desaturase and can be functionally replaced by the rat stearoyl-CoA desaturase gene.](http://www.ncbi.nlm.nih.gov/pubmed/1978720) *J Biol Chem.* 1990; **265:**20144-9.

Donzeau M, Bourdineaud JP, Lauquin GJ. [Regulation by low temperatures and anaerobiosis of a yeast gene specifying a putative GPI-anchored plasma membrane protein [corrected].](http://www.ncbi.nlm.nih.gov/pubmed/8733242) *Mol Microbiol.* 1996; **20:** 449-59.

Sertil O, Cohen BD, Davies KJ, Lowry CV. [The DAN1 gene of S. cerevisiae is regulated in parallel with the hypoxic genes, but by a different mechanism.](http://www.ncbi.nlm.nih.gov/pubmed/9224891) *Gene.* 1997; **192:** 199-205.

Tamarit J, Belli G, Cabiscol E, Herrero E, Ros J. [Biochemical characterization of yeast mitochondrial Grx5 monothiol glutaredoxin.](http://www.ncbi.nlm.nih.gov/pubmed/12730244) *J Biol Chem.* 2003; **278:** 25745-51.

Moye-Rowley WS. [Regulation of the transcriptional response to oxidative stress in fungi: similarities and differences.](http://www.ncbi.nlm.nih.gov/pubmed/12796283)*Eukaryot Cell.* 2003; **2:** 381-9.

Gulshan K, Lee SS, Moye-Rowley WS. [Differential oxidant tolerance determined by the key transcription factor Yap1 is controlled by levels of the Yap1-binding protein, Ybp1.](http://www.ncbi.nlm.nih.gov/pubmed/21844193) *J Biol Chem.* 2011; **286:** 34071-81.

Gulshan K, Rovinsky SA, Coleman ST, Moye-Rowley WS. [Oxidant-specific folding of Yap1p regulates both transcriptional activation and nuclear localization.](http://www.ncbi.nlm.nih.gov/pubmed/16219769) *J Biol Chem.* 2005; **280:** 40524-33.

Choi JH, Lou W, Vancura A. [A novel membrane-bound glutathione S-transferase functions in the stationary phase of the yeast Saccharomyces cerevisiae.](http://www.ncbi.nlm.nih.gov/pubmed/9792709) *J Biol Chem.* 1998; **273:** 29915-22.

Izquierdo A, Casas C, Mühlenhoff U, Lillig CH, Herrero E. [Saccharomyces cerevisiae Grx6 and Grx7 are monothiol glutaredoxins associated with the early secretory pathway.](http://www.ncbi.nlm.nih.gov/pubmed/18503006) *Eukaryot Cell.* 2008; **7:** 1415-26.

Cooper KF, Mallory MJ, Strich R. [Oxidative stress-induced destruction of the yeast C-type cyclin Ume3p requires phosphatidylinositol-specific phospholipase C and the 26S proteasome.](http://www.ncbi.nlm.nih.gov/pubmed/10207058) *Mol Cell Biol.* 1999; **19:** 3338-48.

Charizanis C, Juhnke H, Krems B, Entian KD. [The mitochondrial cytochrome c peroxidase Ccp1 of Saccharomyces cerevisiae is involved in conveying an oxidative stress signal to the transcription factor Pos9 (Skn7).](http://www.ncbi.nlm.nih.gov/pubmed/10589830) *Mol Gen Genet.*1999; **262:** 437-47.

He XJ, Fassler JS. [Identification of novel Yap1p and Skn7p binding sites involved in the oxidative stress response of Saccharomyces cerevisiae.](http://www.ncbi.nlm.nih.gov/pubmed/16313629) *Mol Microbiol.* 2005; **58:** 1454-67.

Mulford KE, Fassler JS. [Association of the Skn7 and Yap1 transcription factors in the Saccharomyces cerevisiae oxidative stress response.](http://www.ncbi.nlm.nih.gov/pubmed/21478431) *Eukaryot Cell.* 2011; **10:** 761-9.

Delaunay A, Pflieger D, Barrault MB, Vinh J, Toledano MB. [A thiol peroxidase is an H2O2 receptor and redox-transducer in gene activation.](http://www.ncbi.nlm.nih.gov/pubmed/12437921) *Cell.* 2002; **111:** 471-81.

Farrugia G, Balzan R. [Oxidative stress and programmed cell death in yeast.](http://www.ncbi.nlm.nih.gov/pubmed/22737670) *Front Oncol.* 2012; **2:** 64.

Iwai K, Naganuma A, Kuge S. [Peroxiredoxin Ahp1 acts as a receptor for alkylhydroperoxides to induce disulfide bond formation in the Cad1 transcription factor.](http://www.ncbi.nlm.nih.gov/pubmed/20145245) *J Biol Chem.* 2010; **285:** 10597-604.

Veal EA, Ross SJ, Malakasi P, Peacock E, Morgan BA. [Ybp1 is required for the hydrogen peroxide-induced oxidation of the Yap1 transcription factor.](http://www.ncbi.nlm.nih.gov/pubmed/12743123) *J Biol Chem.* 2003; **278:** 30896-904.

Gulshan K, Rovinsky SA, Moye-Rowley WS. [YBP1 and its homologue YBP2/YBH1 influence oxidative-stress tolerance by nonidentical mechanisms in Saccharomyces cerevisiae.](http://www.ncbi.nlm.nih.gov/pubmed/15075262) *Eukaryot Cell.* 2004; **3:** 318-30.

Okazaki S, Tachibana T, Naganuma A, Mano N, Kuge S. [Multistep disulfide bond formation in Yap1 is required for sensing and transduction of H2O2 stress signal.](http://www.ncbi.nlm.nih.gov/pubmed/17707237) *Mol Cell.* 2007; **27:** 675-88.

Rep M, Proft M, Remize F, Tamás M, Serrano R, Thevelein JM, Hohmann S. [The Saccharomyces cerevisiae Sko1p transcription factor mediates HOG pathway-dependent osmotic regulation of a set of genes encoding enzymes implicated in protection from oxidative damage.](http://www.ncbi.nlm.nih.gov/pubmed/11401713) *Mol Microbiol.* 2001; **40:** 1067-83.

Dumond H, Danielou N, Pinto M, Bolotin-Fukuhara M. [A large-scale study of Yap1p-dependent genes in normal aerobic and H2O2-stress conditions: the role of Yap1p in cell proliferation control in yeast.](http://www.ncbi.nlm.nih.gov/pubmed/10844671) *Mol Microbiol.* 2000; **36:** 830-45.

Gasch AP, Spellman PT, Kao CM, Carmel-Harel O, Eisen MB, Storz G, Botstein D, Brown PO. [Genomic expression programs in the response of yeast cells to environmental changes.](http://www.ncbi.nlm.nih.gov/pubmed/11102521) *Mol Biol Cell.* 2000; **11:** 4241-57.

He XJ, Mulford KE, Fassler JS. [Oxidative stress function of the Saccharomyces cerevisiae Skn7 receiver domain.](http://www.ncbi.nlm.nih.gov/pubmed/19304952)*Eukaryot Cell.* 2009; **8:** 768-78.

Ayer A, Fellermeier S, Fife C, Li SS, Smits G, Meyer AJ, Dawes IW, Perrone GG. [A genome-wide screen in yeast identifies specific oxidative stress genes required for the maintenance of sub-cellular redox homeostasis.](http://www.ncbi.nlm.nih.gov/pubmed/22970195) *PLoS One.*2012; **7:** e44278.

Hasan R, Leroy C, Isnard AD, Labarre J, Boy-Marcotte E, Toledano MB. [The control of the yeast H2O2 response by the Msn2/4 transcription factors.](http://www.ncbi.nlm.nih.gov/pubmed/12100562) *Mol Microbiol.* 2002; **45:** 233-41.

Yamamoto A, Ueda J, Yamamoto N, Hashikawa N, Sakurai H. [Role of heat shock transcription factor in Saccharomyces cerevisiae oxidative stress response.](http://www.ncbi.nlm.nih.gov/pubmed/17586717) *Eukaryot Cell.* 2007; **6:** 1373-9.

Morgan BA, Banks GR, Toone WM, Raitt D, Kuge S, Johnston LH. [The Skn7 response regulator controls gene expression in the oxidative stress response of the budding yeast Saccharomyces cerevisiae.](http://www.ncbi.nlm.nih.gov/pubmed/9118942) *EMBO J.* 1997; **16:** 1035-44.

Fernandes L, Rodrigues-Pousada C, Struhl K. [Yap, a novel family of eight bZIP proteins in Saccharomyces cerevisiae with distinct biological functions.](http://www.ncbi.nlm.nih.gov/pubmed/9372930) *Mol Cell Biol.* 1997; **17:** 6982-93.

Kuge S, Jones N. [YAP1 dependent activation of TRX2 is essential for the response of Saccharomyces cerevisiae to oxidative stress by hydroperoxides.](http://www.ncbi.nlm.nih.gov/pubmed/8313910) *EMBO J.* 1994; **13:** 655-64.

Coleman ST, Epping EA, Steggerda SM, Moye-Rowley WS. [Yap1p activates gene transcription in an oxidant-specific fashion.](http://www.ncbi.nlm.nih.gov/pubmed/10567555) *Mol Cell Biol.* 1999; **19:** 8302-13.

Delaunay A, Isnard AD, Toledano MB. [H2O2 sensing through oxidation of the Yap1 transcription factor.](http://www.ncbi.nlm.nih.gov/pubmed/11013218) *EMBO J.* 2000;**19:** 5157-66.

Isoyama T, Murayama A, Nomoto A, Kuge S. [Nuclear import of the yeast AP-1-like transcription factor Yap1p is mediated by transport receptor Pse1p, and this import step is not affected by oxidative stress.](http://www.ncbi.nlm.nih.gov/pubmed/11274141) *J Biol Chem.* 2001;**276:** 21863-9.

Alepuz PM, Jovanovic A, Reiser V, Ammerer G. [Stress-induced map kinase Hog1 is part of transcription activation complexes.](http://www.ncbi.nlm.nih.gov/pubmed/11336700) *Mol Cell.* 2001; **7:** 767-77.

Tachibana T, Okazaki S, Murayama A, Naganuma A, Nomoto A, Kuge S. [A major peroxiredoxin-induced activation of Yap1 transcription factor is mediated by reduction-sensitive disulfide bonds and reveals a low level of transcriptional activation.](http://www.ncbi.nlm.nih.gov/pubmed/19106090) *J Biol Chem.* 2009; **284:** 4464-72.

Okazaki S, Naganuma A, Kuge S. [Peroxiredoxin-mediated redox regulation of the nuclear localization of Yap1, a transcription factor in budding yeast.](http://www.ncbi.nlm.nih.gov/pubmed/15706081) *Antioxid Redox Signal.* 2005; **7:** 327-34.

Cohen TJ, Lee K, Rutkowski LH, Strich R. [Ask10p mediates the oxidative stress-induced destruction of the Saccharomyces cerevisiae C-type cyclin Ume3p/Srb11p.](http://www.ncbi.nlm.nih.gov/pubmed/14555478) *Eukaryot Cell.* 2003; **2:** 962-70.

Stephen DW, Rivers SL, Jamieson DJ. [The role of the YAP1 and YAP2 genes in the regulation of the adaptive oxidative stress responses of Saccharomyces cerevisiae.](http://www.ncbi.nlm.nih.gov/pubmed/7565103) *Mol Microbiol.* 1995; **16:** 415-23.

Kuge S, Toda T, Iizuka N, Nomoto A. [Crm1 (XpoI) dependent nuclear export of the budding yeast transcription factor yAP-1 is sensitive to oxidative stress.](http://www.ncbi.nlm.nih.gov/pubmed/9797454) *Genes Cells.* 1998; **3:** 521-32.

Izawa S, Maeda K, Sugiyama K, Mano J, Inoue Y, Kimura A. [Thioredoxin deficiency causes the constitutive activation of Yap1, an AP-1-like transcription factor in Saccharomyces cerevisiae.](http://www.ncbi.nlm.nih.gov/pubmed/10497208) *J Biol Chem.* 1999; **274:** 28459-65.

Kuge S, Arita M, Murayama A, Maeta K, Izawa S, Inoue Y, Nomoto A. [Regulation of the yeast Yap1p nuclear export signal is mediated by redox signal-induced reversible disulfide bond formation.](http://www.ncbi.nlm.nih.gov/pubmed/11509657) *Mol Cell Biol.* 2001; **21:** 6139-50.

Carmel-Harel O, Stearman R, Gasch AP, Botstein D, Brown PO, Storz G. [Role of thioredoxin reductase in the Yap1p-dependent response to oxidative stress in Saccharomyces cerevisiae.](http://www.ncbi.nlm.nih.gov/pubmed/11169101) *Mol Microbiol.* 2001; **39:** 595-605.

Cohen BA, Pilpel Y, Mitra RD, Church GM. [Discrimination between paralogs using microarray analysis: application to the Yap1p and Yap2p transcriptional networks.](http://www.ncbi.nlm.nih.gov/pubmed/12006656) *Mol Biol Cell.* 2002; **13:** 1608-14.

Rodrigues-Pousada CA, Nevitt T, Menezes R, Azevedo D, Pereira J, Amaral C. [Yeast activator proteins and stress response: an overview.](http://www.ncbi.nlm.nih.gov/pubmed/15165897) *FEBS Lett.* 2004; **567:** 80-5.

Azevedo D, Nascimento L, Labarre J, Toledano MB, Rodrigues-Pousada C. [The S. cerevisiae Yap1 and Yap2 transcription factors share a common cadmium-sensing domain.](http://www.ncbi.nlm.nih.gov/pubmed/17187783) *FEBS Lett.* 2007; **581:** 187-95.

Watson PG, Jakeman C, Ozturk M, Barnett MF, Barnett F, Khaw KT. [The complications of trabeculectomy (a 20-year follow-up).](http://www.ncbi.nlm.nih.gov/pubmed/2209905) *Eye (Lond).* 1990; **4 ( Pt 3):** 425-38.

Gan ZR. [Yeast thioredoxin genes.](http://www.ncbi.nlm.nih.gov/pubmed/1988444) *J Biol Chem.* 1991; **266:** 1692-6.

Kwon M, Chong S, Han S, Kim K. [Oxidative stresses elevate the expression of cytochrome c peroxidase in Saccharomyces cerevisiae.](http://www.ncbi.nlm.nih.gov/pubmed/12957710) *Biochim Biophys Acta.* 2003; **1623:** 1-5.

Juhnke H, Charizanis C, Latifi F, Krems B, Entian KD. [The essential protein fap7 is involved in the oxidative stress response of Saccharomyces cerevisiae.](http://www.ncbi.nlm.nih.gov/pubmed/10692169) *Mol Microbiol.* 2000; **35:** 936-48.

Jin C, Strich R, Cooper KF. [Slt2p phosphorylation induces cyclin C nuclear-to-cytoplasmic translocation in response to oxidative stress.](http://www.ncbi.nlm.nih.gov/pubmed/24554767) *Mol Biol Cell.* 2014; **25:** 1396-407.

Krasley E, Cooper KF, Mallory MJ, Dunbrack R, Strich R. [Regulation of the oxidative stress response through Slt2p-dependent destruction of cyclin C in Saccharomyces cerevisiae.](http://www.ncbi.nlm.nih.gov/pubmed/16387872) *Genetics.* 2006; **172:** 1477-86.

Miranda-Vizuete A, Damdimopoulos AE, Spyrou G. [The mitochondrial thioredoxin system.](http://www.ncbi.nlm.nih.gov/pubmed/11213484) *Antioxid Redox Signal.* 2000; **2:**801-10.

Garrido EO, Grant CM. [Role of thioredoxins in the response of Saccharomyces cerevisiae to oxidative stress induced by hydroperoxides.](http://www.ncbi.nlm.nih.gov/pubmed/11929546) *Mol Microbiol.* 2002; **43:** 993-1003.

Wong CM, Siu KL, Jin DY. [Peroxiredoxin-null yeast cells are hypersensitive to oxidative stress and are genomically unstable.](http://www.ncbi.nlm.nih.gov/pubmed/15051715) *J Biol Chem.* 2004; **279:** 23207-13.

Inoue Y, Matsuda T, Sugiyama K, Izawa S, Kimura A. [Genetic analysis of glutathione peroxidase in oxidative stress response of Saccharomyces cerevisiae.](http://www.ncbi.nlm.nih.gov/pubmed/10480913) *J Biol Chem.* 1999; **274:** 27002-9.

Avery AM, Avery SV. [Saccharomyces cerevisiae expresses three phospholipid hydroperoxide glutathione peroxidases.](http://www.ncbi.nlm.nih.gov/pubmed/11445588) *J Biol Chem.* 2001; **276:** 33730-5.

Trotter EW, Grant CM. [Overlapping roles of the cytoplasmic and mitochondrial redox regulatory systems in the yeast Saccharomyces cerevisiae.](http://www.ncbi.nlm.nih.gov/pubmed/15701801) *Eukaryot Cell.* 2005; **4:** 392-400.

Biteau B, Labarre J, Toledano MB. [ATP-dependent reduction of cysteine-sulphinic acid by S. cerevisiae sulphiredoxin.](http://www.ncbi.nlm.nih.gov/pubmed/14586471)*Nature.* 2003; **425:** 980-4.

Lee J, Spector D, Godon C, Labarre J, Toledano MB. [A new antioxidant with alkyl hydroperoxide defense properties in yeast.](http://www.ncbi.nlm.nih.gov/pubmed/9988687) *J Biol Chem.* 1999; **274:** 4537-44.

Park SG, Cha MK, Jeong W, Kim IH. [Distinct physiological functions of thiol peroxidase isoenzymes in Saccharomyces cerevisiae.](http://www.ncbi.nlm.nih.gov/pubmed/10681558) *J Biol Chem.* 2000; **275:** 5723-32.

Izawa S, Kuroki N, Inoue Y. [Nuclear thioredoxin peroxidase Dot5 in Saccharomyces cerevisiae: roles in oxidative stress response and disruption of telomeric silencing.](http://www.ncbi.nlm.nih.gov/pubmed/12925864) *Appl Microbiol Biotechnol.* 2004; **64:** 120-4.

Hinnebusch AG. [Translational regulation of GCN4 and the general amino acid control of yeast.](http://www.ncbi.nlm.nih.gov/pubmed/16153175) *Annu Rev Microbiol.* 2005;**59:** 407-50.

Ma M, Liu ZL. [Comparative transcriptome profiling analyses during the lag phase uncover YAP1, PDR1, PDR3, RPN4, and HSF1 as key regulatory genes in genomic adaptation to the lignocellulose derived inhibitor HMF for Saccharomyces cerevisiae.](http://www.ncbi.nlm.nih.gov/pubmed/21106074) *BMC Genomics.* 2010; **11:** 660.

Lisowsky T. [A high copy number of yeast gamma-glutamylcysteine synthetase suppresses a nuclear mutation affecting mitochondrial translation.](http://www.ncbi.nlm.nih.gov/pubmed/8100487) *Curr Genet.* 1993; **23:** 408-13.

Grant CM, MacIver FH, Dawes IW. [Glutathione synthetase is dispensable for growth under both normal and oxidative stress conditions in the yeast Saccharomyces cerevisiae due to an accumulation of the dipeptide gamma-glutamylcysteine.](http://www.ncbi.nlm.nih.gov/pubmed/9307967) *Mol Biol Cell.* 1997; **8:** 1699-707.

Bao R, Zhang Y, Lou X, Zhou CZ, Chen Y. [Structural and kinetic analysis of Saccharomyces cerevisiae thioredoxin Trx1: implications for the catalytic mechanism of GSSG reduced by the thioredoxin system.](http://www.ncbi.nlm.nih.gov/pubmed/19362171) *Biochim Biophys Acta.* 2009;**1794:** 1218-23.

Rodríguez-Manzaneque MT, Tamarit J, Bellí G, Ros J, Herrero E. [Grx5 is a mitochondrial glutaredoxin required for the activity of iron/sulfur enzymes.](http://www.ncbi.nlm.nih.gov/pubmed/11950925) *Mol Biol Cell.* 2002; **13:** 1109-21.

Bellí G, Molina MM, García-Martínez J, Pérez-Ortín JE, Herrero E. [Saccharomyces cerevisiae glutaredoxin 5-deficient cells subjected to continuous oxidizing conditions are affected in the expression of specific sets of genes.](http://www.ncbi.nlm.nih.gov/pubmed/14722110) *J Biol Chem.*2004; **279:** 12386-95.

Kim KD, Chung WH, Kim HJ, Lee KC, Roe JH. [Monothiol glutaredoxin Grx5 interacts with Fe-S scaffold proteins Isa1 and Isa2 and supports Fe-S assembly and DNA integrity in mitochondria of fission yeast.](http://www.ncbi.nlm.nih.gov/pubmed/20085751) *Biochem Biophys Res Commun.*2010; **392:** 467-72.

Molina-Navarro MM, Casas C, Piedrafita L, Bellí G, Herrero E. [Prokaryotic and eukaryotic monothiol glutaredoxins are able to perform the functions of Grx5 in the biogenesis of Fe/S clusters in yeast mitochondria.](http://www.ncbi.nlm.nih.gov/pubmed/16566929) *FEBS Lett.* 2006; **580:**2273-80.

Barreto L, Garcerá A, Jansson K, Sunnerhagen P, Herrero E. [A peroxisomal glutathione transferase of Saccharomyces cerevisiae is functionally related to sulfur amino acid metabolism.](http://www.ncbi.nlm.nih.gov/pubmed/16936141) *Eukaryot Cell.* 2006; **5:** 1748-59.

Cohen G, Fessl F, Traczyk A, Rytka J, Ruis H. [Isolation of the catalase A gene of Saccharomyces cerevisiae by complementation of the cta1 mutation.](http://www.ncbi.nlm.nih.gov/pubmed/3897793) *Mol Gen Genet.* 1985; **200:** 74-9.

Petrova VY, Drescher D, Kujumdzieva AV, Schmitt MJ. [Dual targeting of yeast catalase A to peroxisomes and mitochondria.](http://www.ncbi.nlm.nih.gov/pubmed/14998369) *Biochem J.* 2004; **380:** 393-400.

Goehring AS, Rivers DM, Sprague GF Jr. [Attachment of the ubiquitin-related protein Urm1p to the antioxidant protein Ahp1p.](http://www.ncbi.nlm.nih.gov/pubmed/14555475) *Eukaryot Cell.* 2003; **2:** 930-6.

Wong CM, Zhou Y, Ng RW, Kung Hf HF, Jin DY. [Cooperation of yeast peroxiredoxins Tsa1p and Tsa2p in the cellular defense against oxidative and nitrosative stress.](http://www.ncbi.nlm.nih.gov/pubmed/11741925) *J Biol Chem.* 2002; **277:** 5385-94.

Jin C, Parshin AV, Daly I, Strich R, Cooper KF. [The cell wall sensors Mtl1, Wsc1, and Mid2 are required for stress-induced nuclear to cytoplasmic translocation of cyclin C and programmed cell death in yeast.](http://www.ncbi.nlm.nih.gov/pubmed/24260614) *Oxid Med Cell Longev.*2013; **2013:** 320823.

Kuchin S, Yeghiayan P, Carlson M. [Cyclin-dependent protein kinase and cyclin homologs SSN3 and SSN8 contribute to transcriptional control in yeast.](http://www.ncbi.nlm.nih.gov/pubmed/7732022) *Proc Natl Acad Sci U S A.* 1995; **92:** 4006-10.

Nogae I, Johnston M. [Isolation and characterization of the ZWF1 gene of Saccharomyces cerevisiae, encoding glucose-6-phosphate dehydrogenase.](http://www.ncbi.nlm.nih.gov/pubmed/2269430) *Gene.* 1990; **96:** 161-9.

Cherest H, Surdin-Kerjan Y. [Genetic analysis of a new mutation conferring cysteine auxotrophy in Saccharomyces cerevisiae: updating of the sulfur metabolism pathway.](http://www.ncbi.nlm.nih.gov/pubmed/1732168) *Genetics.* 1992; **130:** 51-8.

Schaaff I, Hohmann S, Zimmermann FK. [Molecular analysis of the structural gene for yeast transaldolase.](http://www.ncbi.nlm.nih.gov/pubmed/2185015) *Eur J Biochem.*1990; **188:** 597-603.

Puria R, Mannan MA, Chopra-Dewasthaly R, Ganesan K. [Critical role of RPI1 in the stress tolerance of yeast during ethanolic fermentation.](http://www.ncbi.nlm.nih.gov/pubmed/19678848) *FEMS Yeast Res.* 2009; **9:** 1161-71.

McNeil JB, McIntosh EM, Taylor BV, Zhang FR, Tang S, Bognar AL. [Cloning and molecular characterization of three genes, including two genes encoding serine hydroxymethyltransferases, whose inactivation is required to render yeast auxotrophic for glycine.](http://www.ncbi.nlm.nih.gov/pubmed/8132653) *J Biol Chem.* 1994; **269:** 9155-65.

Kumar D, Viberg J, Nilsson AK, Chabes A. [Highly mutagenic and severely imbalanced dNTP pools can escape detection by the S-phase checkpoint.](http://www.ncbi.nlm.nih.gov/pubmed/20215435) *Nucleic Acids Res.* 2010; **38:** 3975-83.

Sia RA, Herald HA, Lew DJ. [Cdc28 tyrosine phosphorylation and the morphogenesis checkpoint in budding yeast.](http://www.ncbi.nlm.nih.gov/pubmed/8930890) *Mol Biol Cell.* 1996; **7:** 1657-66.

Leonhardt SA, Fearson K, Danese PN, Mason TL. [HSP78 encodes a yeast mitochondrial heat shock protein in the Clp family of ATP-dependent proteases.](http://www.ncbi.nlm.nih.gov/pubmed/8413229) *Mol Cell Biol.* 1993; **13:** 6304-13.

Yun CW, Bauler M, Moore RE, Klebba PE, Philpott CC. [The role of the FRE family of plasma membrane reductases in the uptake of siderophore-iron in Saccharomyces cerevisiae.](http://www.ncbi.nlm.nih.gov/pubmed/11120744) *J Biol Chem.* 2001; **276:** 10218-23.

Puig S, Askeland E, Thiele DJ. [Coordinated remodeling of cellular metabolism during iron deficiency through targeted mRNA degradation.](http://www.ncbi.nlm.nih.gov/pubmed/15652485) *Cell.* 2005; **120:** 99-110.

Protchenko O, Philpott CC. [Regulation of intracellular heme levels by HMX1, a homologue of heme oxygenase, in Saccharomyces cerevisiae.](http://www.ncbi.nlm.nih.gov/pubmed/12840010) *J Biol Chem.* 2003; **278:** 36582-7.

Stolz J, Hoja U, Meier S, Sauer N, Schweizer E. [Identification of the plasma membrane H+-biotin symporter of Saccharomyces cerevisiae by rescue of a fatty acid-auxotrophic mutant.](http://www.ncbi.nlm.nih.gov/pubmed/10373489) *J Biol Chem.* 1999; **274:** 18741-6.

Pekary AE, Lukaski HC, Mena I, Hershman JM. [Processing of TRH precursor peptides in rat brain and pituitary is zinc dependent.](http://www.ncbi.nlm.nih.gov/pubmed/1800945) *Peptides.* 1991; **12:** 1025-32.

Alves R, Herrero E, Sorribas A. [Predictive reconstruction of the mitochondrial iron-sulfur cluster assembly metabolism. II. Role of glutaredoxin Grx5.](http://www.ncbi.nlm.nih.gov/pubmed/15382238) *Proteins.* 2004; **57:** 481-92.

Molina MM, Bellí G, de la Torre MA, Rodríguez-Manzaneque MT, Herrero E. [Nuclear monothiol glutaredoxins of Saccharomyces cerevisiae can function as mitochondrial glutaredoxins.](http://www.ncbi.nlm.nih.gov/pubmed/15456753) *J Biol Chem.* 2004; **279:** 51923-30.

Rodríguez-Manzaneque MT, Ros J, Cabiscol E, Sorribas A, Herrero E. [Grx5 glutaredoxin plays a central role in protection against protein oxidative damage in Saccharomyces cerevisiae.](http://www.ncbi.nlm.nih.gov/pubmed/10567543) *Mol Cell Biol.* 1999; **19:** 8180-90.

Zhang B, Bandyopadhyay S, Shakamuri P, Naik SG, Huynh BH, Couturier J, Rouhier N, Johnson MK. [Monothiol glutaredoxins can bind linear [Fe3S4]+ and [Fe4S4]2+ clusters in addition to [Fe2S2]2+ clusters: spectroscopic characterization and functional implications.](http://www.ncbi.nlm.nih.gov/pubmed/24032439) *J Am Chem Soc.* 2013; **135:** 15153-64.

Otsuga D, Keegan BR, Brisch E, Thatcher JW, Hermann GJ, Bleazard W, Shaw JM. [The dynamin-related GTPase, Dnm1p, controls mitochondrial morphology in yeast.](http://www.ncbi.nlm.nih.gov/pubmed/9786946) *J Cell Biol.* 1998; **143:** 333-49.

Johnson DI. [Cdc42: An essential Rho-type GTPase controlling eukaryotic cell polarity.](http://www.ncbi.nlm.nih.gov/pubmed/10066831) *Microbiol Mol Biol Rev.* 1999; **63:**54-105.

Dohlman HG. [G proteins and pheromone signaling.](http://www.ncbi.nlm.nih.gov/pubmed/11826266) *Annu Rev Physiol.* 2002; **64:** 129-52.

Bardwell L, Cook JG, Voora D, Baggott DM, Martinez AR, Thorner J. [Repression of yeast Ste12 transcription factor by direct binding of unphosphorylated Kss1 MAPK and its regulation by the Ste7 MEK.](http://www.ncbi.nlm.nih.gov/pubmed/9744865) *Genes Dev.* 1998; **12:** 2887-98.

Blondel M, Galan JM, Chi Y, Lafourcade C, Longaretti C, Deshaies RJ, Peter M. [Nuclear-specific degradation of Far1 is controlled by the localization of the F-box protein Cdc4.](http://www.ncbi.nlm.nih.gov/pubmed/11080155) *EMBO J.* 2000; **19:** 6085-97.

Bardwell L, Cook JG, Chang EC, Cairns BR, Thorner J. [Signaling in the yeast pheromone response pathway: specific and high-affinity interaction of the mitogen-activated protein (MAP) kinases Kss1 and Fus3 with the upstream MAP kinase kinase Ste7.](http://www.ncbi.nlm.nih.gov/pubmed/8668180) *Mol Cell Biol.* 1996; **16:** 3637-50.

Gulli MP, Jaquenoud M, Shimada Y, Niederhäuser G, Wiget P, Peter M. [Phosphorylation of the Cdc42 exchange factor Cdc24 by the PAK-like kinase Cla4 may regulate polarized growth in yeast.](http://www.ncbi.nlm.nih.gov/pubmed/11106754) *Mol Cell.* 2000; **6:** 1155-67.

Dohlman HG, Thorner JW. [Regulation of G protein-initiated signal transduction in yeast: paradigms and principles.](http://www.ncbi.nlm.nih.gov/pubmed/11395421) *Annu Rev Biochem.* 2001; **70:** 703-54.

Köhler T, Wesche S, Taheri N, Braus GH, Mösch HU. [Dual role of the Saccharomyces cerevisiae TEA/ATTS family transcription factor Tec1p in regulation of gene expression and cellular development.](http://www.ncbi.nlm.nih.gov/pubmed/12455687) *Eukaryot Cell.* 2002; **1:** 673-86.

Cullen PJ, Sabbagh W Jr, Graham E, Irick MM, van Olden EK, Neal C, Delrow J, Bardwell L, Sprague GF Jr. [A signaling mucin at the head of the Cdc42- and MAPK-dependent filamentous growth pathway in yeast.](http://www.ncbi.nlm.nih.gov/pubmed/15256499) *Genes Dev.* 2004; **18:**1695-708.

Madhani HD, Styles CA, Fink GR. [MAP kinases with distinct inhibitory functions impart signaling specificity during yeast differentiation.](http://www.ncbi.nlm.nih.gov/pubmed/9393860) *Cell.* 1997; **91:** 673-84.

Oehlen L, Cross FR. [The mating factor response pathway regulates transcription of TEC1, a gene involved in pseudohyphal differentiation of Saccharomyces cerevisiae.](http://www.ncbi.nlm.nih.gov/pubmed/9657388) *FEBS Lett.* 1998; **429:** 83-8.

Robertson LS, Fink GR. [The three yeast A kinases have specific signaling functions in pseudohyphal growth.](http://www.ncbi.nlm.nih.gov/pubmed/9811878) *Proc Natl Acad Sci U S A.* 1998; **95:** 13783-7.

Conlan RS, Tzamarias D. [Sfl1 functions via the co-repressor Ssn6-Tup1 and the cAMP-dependent protein kinase Tpk2.](http://www.ncbi.nlm.nih.gov/pubmed/11399075)*J Mol Biol.* 2001; **309:** 1007-15.

Feng Y, Davis NG. [Akr1p and the type I casein kinases act prior to the ubiquitination step of yeast endocytosis: Akr1p is required for kinase localization to the plasma membrane.](http://www.ncbi.nlm.nih.gov/pubmed/10866691) *Mol Cell Biol.* 2000; **20:** 5350-9.

Hicke L, Riezman H. [Ubiquitination of a yeast plasma membrane receptor signals its ligand-stimulated endocytosis.](http://www.ncbi.nlm.nih.gov/pubmed/8565073) *Cell.*1996; **84:** 277-87.

Feng Y, Song LY, Kincaid E, Mahanty SK, Elion EA. [Functional binding between Gbeta and the LIM domain of Ste5 is required to activate the MEKK Ste11.](http://www.ncbi.nlm.nih.gov/pubmed/9501067) *Curr Biol.* 1998; **8:** 267-78.

Burkholder AC, Hartwell LH. [The yeast alpha-factor receptor: structural properties deduced from the sequence of the STE2 gene.](http://www.ncbi.nlm.nih.gov/pubmed/3001640) *Nucleic Acids Res.* 1985; **13:** 8463-75.

Xu BE, Skowronek KR, Kurjan J. [The N terminus of Saccharomyces cerevisiae Sst2p plays an RGS-domain-independent, Mpt5p-dependent role in recovery from pheromone arrest.](http://www.ncbi.nlm.nih.gov/pubmed/11779797) *Genetics.* 2001; **159:** 1559-71.

Wang Y, Ge Q, Houston D, Thorner J, Errede B, Dohlman HG. [Regulation of Ste7 ubiquitination by Ste11 phosphorylation and the Skp1-Cullin-F-box complex.](http://www.ncbi.nlm.nih.gov/pubmed/12668671) *J Biol Chem.* 2003; **278:** 22284-9.

Hao N, Yildirim N, Wang Y, Elston TC, Dohlman HG. [Regulators of G protein signaling and transient activation of signaling: experimental and computational analysis reveals negative and positive feedback controls on G protein activity.](http://www.ncbi.nlm.nih.gov/pubmed/12968019) *J Biol Chem.* 2003; **278:** 46506-15.

Grimshaw SJ, Mott HR, Stott KM, Nielsen PR, Evetts KA, Hopkins LJ, Nietlispach D, Owen D. [Structure of the sterile alpha motif (SAM) domain of the Saccharomyces cerevisiae mitogen-activated protein kinase pathway-modulating protein STE50 and analysis of its interaction with the STE11 SAM.](http://www.ncbi.nlm.nih.gov/pubmed/14573615) *J Biol Chem.* 2004; **279:** 2192-201.

Herskowitz I. [MAP kinase pathways in yeast: for mating and more.](http://www.ncbi.nlm.nih.gov/pubmed/7834739) *Cell.* 1995; **80:** 187-97.

Bardwell L. [A walk-through of the yeast mating pheromone response pathway.](http://www.ncbi.nlm.nih.gov/pubmed/15374648) *Peptides.* 2004; **25:** 1465-76.

Kofahl B, Klipp E. [Modelling the dynamics of the yeast pheromone pathway.](http://www.ncbi.nlm.nih.gov/pubmed/15300679) *Yeast.* 2004; **21:** 831-50.

Villasmil ML, Ansbach A, Nickels JT Jr. [The putative lipid transporter, Arv1, is required for activating pheromone-induced MAP kinase signaling in Saccharomyces cerevisiae.](http://www.ncbi.nlm.nih.gov/pubmed/21098723) *Genetics.* 2011; **187:** 455-65.

Merlini L, Dudin O, Martin SG. [Mate and fuse: how yeast cells do it.](http://www.ncbi.nlm.nih.gov/pubmed/23466674) *Open Biol.* 2013; **3:** 130008.

Hagen DC, McCaffrey G, Sprague GF Jr. [Evidence the yeast STE3 gene encodes a receptor for the peptide pheromone a factor: gene sequence and implications for the structure of the presumed receptor.](http://www.ncbi.nlm.nih.gov/pubmed/3006051) *Proc Natl Acad Sci U S A.* 1986;**83:** 1418-22.

Printen JA, Sprague GF Jr. [Protein-protein interactions in the yeast pheromone response pathway: Ste5p interacts with all members of the MAP kinase cascade.](http://www.ncbi.nlm.nih.gov/pubmed/7851759) *Genetics.* 1994; **138:** 609-19.

Marcus S, Polverino A, Barr M, Wigler M. [Complexes between STE5 and components of the pheromone-responsive mitogen-activated protein kinase module.](http://www.ncbi.nlm.nih.gov/pubmed/8052657) *Proc Natl Acad Sci U S A.* 1994; **91:** 7762-6.

Wang Y, Elion EA. [Nuclear export and plasma membrane recruitment of the Ste5 scaffold are coordinated with oligomerization and association with signal transduction components.](http://www.ncbi.nlm.nih.gov/pubmed/12808050) *Mol Biol Cell.* 2003; **14:** 2543-58.

Heinrich M, Köhler T, Mösch HU. [Role of Cdc42-Cla4 interaction in the pheromone response of Saccharomyces cerevisiae.](http://www.ncbi.nlm.nih.gov/pubmed/17189484) *Eukaryot Cell.* 2007; **6:** 317-27.

Tedford K, Kim S, Sa D, Stevens K, Tyers M. [Regulation of the mating pheromone and invasive growth responses in yeast by two MAP kinase substrates.](http://www.ncbi.nlm.nih.gov/pubmed/9094309) *Curr Biol.* 1997; **7:** 228-38.

Chou S, Zhao S, Song Y, Liu H, Nie Q. [Fus3-triggered Tec1 degradation modulates mating transcriptional output during the pheromone response.](http://www.ncbi.nlm.nih.gov/pubmed/18682702) *Mol Syst Biol.* 2008; **4:** 212.

Chou S, Lane S, Liu H. [Regulation of mating and filamentation genes by two distinct Ste12 complexes in Saccharomyces cerevisiae.](http://www.ncbi.nlm.nih.gov/pubmed/16782869) *Mol Cell Biol.* 2006; **26:** 4794-805.

Chen T, Kurjan J. [Saccharomyces cerevisiae Mpt5p interacts with Sst2p and plays roles in pheromone sensitivity and recovery from pheromone arrest.](http://www.ncbi.nlm.nih.gov/pubmed/9154842) *Mol Cell Biol.* 1997; **17:** 3429-39.

Gartner A, Jovanović A, Jeoung DI, Bourlat S, Cross FR, Ammerer G. [Pheromone-dependent G1 cell cycle arrest requires Far1 phosphorylation, but may not involve inhibition of Cdc28-Cln2 kinase, in vivo.](http://www.ncbi.nlm.nih.gov/pubmed/9632750) *Mol Cell Biol.* 1998; **18:**3681-91.

Elion EA, Brill JA, Fink GR. [FUS3 represses CLN1 and CLN2 and in concert with KSS1 promotes signal transduction.](http://www.ncbi.nlm.nih.gov/pubmed/1946350)*Proc Natl Acad Sci U S A.* 1991; **88:** 9392-6.

Elion EA, Satterberg B, Kranz JE. [FUS3 phosphorylates multiple components of the mating signal transduction cascade: evidence for STE12 and FAR1.](http://www.ncbi.nlm.nih.gov/pubmed/8334305) *Mol Biol Cell.* 1993; **4:** 495-510.

Butty AC, Pryciak PM, Huang LS, Herskowitz I, Peter M. [The role of Far1p in linking the heterotrimeric G protein to polarity establishment proteins during yeast mating.](http://www.ncbi.nlm.nih.gov/pubmed/9822386) *Science.* 1998; **282:** 1511-6.

Nern A, Arkowitz RA. [A Cdc24p-Far1p-Gbetagamma protein complex required for yeast orientation during mating.](http://www.ncbi.nlm.nih.gov/pubmed/10087263) *J Cell Biol.* 1999; **144:** 1187-202.

Butty AC, Perrinjaquet N, Petit A, Jaquenoud M, Segall JE, Hofmann K, Zwahlen C, Peter M. [A positive feedback loop stabilizes the guanine-nucleotide exchange factor Cdc24 at sites of polarization.](http://www.ncbi.nlm.nih.gov/pubmed/11927541) *EMBO J.* 2002; **21:** 1565-76.

Bar EE, Ellicott AT, Stone DE. [Gbetagamma recruits Rho1 to the site of polarized growth during mating in budding yeast.](http://www.ncbi.nlm.nih.gov/pubmed/12660244)*J Biol Chem.* 2003; **278:** 21798-804.

Lyons DM, Mahanty SK, Choi KY, Manandhar M, Elion EA. [The SH3-domain protein Bem1 coordinates mitogen-activated protein kinase cascade activation with cell cycle control in Saccharomyces cerevisiae.](http://www.ncbi.nlm.nih.gov/pubmed/8754808) *Mol Cell Biol.* 1996; **16:** 4095-106.

Park HO, Bi E. [Central roles of small GTPases in the development of cell polarity in yeast and beyond.](http://www.ncbi.nlm.nih.gov/pubmed/17347519) *Microbiol Mol Biol Rev.* 2007; **71:** 48-96.

Leeuw T, Fourest-Lieuvin A, Wu C, Chenevert J, Clark K, Whiteway M, Thomas DY, Leberer E. [Pheromone response in yeast: association of Bem1p with proteins of the MAP kinase cascade and actin.](http://www.ncbi.nlm.nih.gov/pubmed/7502048) *Science.* 1995; **270:** 1210-3.

Lamson RE, Winters MJ, Pryciak PM. [Cdc42 regulation of kinase activity and signaling by the yeast p21-activated kinase Ste20.](http://www.ncbi.nlm.nih.gov/pubmed/11940652) *Mol Cell Biol.* 2002; **22:** 2939-51.

Bhunia A, Domadia PN, Xu X, Gingras R, Ni F, Bhattacharjya S. [Equilibrium unfolding of the dimeric SAM domain of MAPKKK Ste11 from the budding yeast: role of the interfacial residues in structural stability and binding.](http://www.ncbi.nlm.nih.gov/pubmed/18092817)*Biochemistry.* 2008; **47:** 651-9.

Song D, Dolan JW, Yuan YL, Fields S. [Pheromone-dependent phosphorylation of the yeast STE12 protein correlates with transcriptional activation.](http://www.ncbi.nlm.nih.gov/pubmed/2026326) *Genes Dev.* 1991; **5:** 741-50.

Madhani HD, Fink GR. [Combinatorial control required for the specificity of yeast MAPK signaling.](http://www.ncbi.nlm.nih.gov/pubmed/9036858) *Science.* 1997; **275:**1314-7.

Ballon DR, Flanary PL, Gladue DP, Konopka JB, Dohlman HG, Thorner J. [DEP-domain-mediated regulation of GPCR signaling responses.](http://www.ncbi.nlm.nih.gov/pubmed/16990133) *Cell.* 2006; **126:** 1079-93.

Chou S, Huang L, Liu H. [Fus3-regulated Tec1 degradation through SCFCdc4 determines MAPK signaling specificity during mating in yeast.](http://www.ncbi.nlm.nih.gov/pubmed/15620356) *Cell.* 2004; **119:** 981-90.

Bao MZ, Schwartz MA, Cantin GT, Yates JR 3rd, Madhani HD. [Pheromone-dependent destruction of the Tec1 transcription factor is required for MAP kinase signaling specificity in yeast.](http://www.ncbi.nlm.nih.gov/pubmed/15620357) *Cell.* 2004; **119:** 991-1000.

Roth AF, Nelson B, Boone C, Davis NG. [Asg7p-Ste3p inhibition of pheromone signaling: regulation of the zygotic transition to vegetative growth.](http://www.ncbi.nlm.nih.gov/pubmed/11073982) *Mol Cell Biol.* 2000; **20:** 8815-25.

Rivers DM, Sprague GF Jr. [Autocrine activation of the pheromone response pathway in matalpha2- cells is attenuated by SST2- and ASG7-dependent mechanisms.](http://www.ncbi.nlm.nih.gov/pubmed/13680367) *Mol Genet Genomics.* 2003; **270:** 225-33.

Tyers M, Futcher B. [Far1 and Fus3 link the mating pheromone signal transduction pathway to three G1-phase Cdc28 kinase complexes.](http://www.ncbi.nlm.nih.gov/pubmed/8395009) *Mol Cell Biol.* 1993; **13:** 5659-69.

Mead J, Bruning AR, Gill MK, Steiner AM, Acton TB, Vershon AK. [Interactions of the Mcm1 MADS box protein with cofactors that regulate mating in yeast.](http://www.ncbi.nlm.nih.gov/pubmed/12052870) *Mol Cell Biol.* 2002; **22:** 4607-21.

Ammerer G. [Identification, purification, and cloning of a polypeptide (PRTF/GRM) that binds to mating-specific promoter elements in yeast.](http://www.ncbi.nlm.nih.gov/pubmed/2159934) *Genes Dev.* 1990; **4:** 299-312.

Keleher CA, Passmore S, Johnson AD. [Yeast repressor alpha 2 binds to its operator cooperatively with yeast protein Mcm1.](http://www.ncbi.nlm.nih.gov/pubmed/2689875) *Mol Cell Biol.* 1989; **9:** 5228-30.

Dohlman HG, Song J, Ma D, Courchesne WE, Thorner J. [Sst2, a negative regulator of pheromone signaling in the yeast Saccharomyces cerevisiae: expression, localization, and genetic interaction and physical association with Gpa1 (the G-protein alpha subunit).](http://www.ncbi.nlm.nih.gov/pubmed/8756677) *Mol Cell Biol.* 1996; **16:** 5194-209.

Apanovitch DM, Slep KC, Sigler PB, Dohlman HG. [Sst2 is a GTPase-activating protein for Gpa1: purification and characterization of a cognate RGS-Galpha protein pair in yeast.](http://www.ncbi.nlm.nih.gov/pubmed/9537998) *Biochemistry.* 1998; **37:** 4815-22.

Yu RC, Pesce CG, Colman-Lerner A, Lok L, Pincus D, Serra E, Holl M, Benjamin K, Gordon A, Brent R. [Negative feedback that improves information transmission in yeast signalling.](http://www.ncbi.nlm.nih.gov/pubmed/19079053) *Nature.* 2008; **456:** 755-61.

Li E, Cismowski MJ, Stone DE. [Phosphorylation of the pheromone-responsive Gbeta protein of Saccharomyces cerevisiae does not affect its mating-specific signaling function.](http://www.ncbi.nlm.nih.gov/pubmed/9671029) *Mol Gen Genet.* 1998; **258:** 608-18.

Deflorio R, Brett ME, Waszczak N, Apollinari E, Metodiev MV, Dubrovskyi O, Eddington D, Arkowitz RA, Stone DE.[Phosphorylation of Gβ is crucial for efficient chemotropism in yeast.](http://www.ncbi.nlm.nih.gov/pubmed/23613469) *J Cell Sci.* 2013; **126:** 2997-3009.

Leberer E, Wu C, Leeuw T, Fourest-Lieuvin A, Segall JE, Thomas DY. [Functional characterization of the Cdc42p binding domain of yeast Ste20p protein kinase.](http://www.ncbi.nlm.nih.gov/pubmed/9009270) *EMBO J.* 1997; **16:** 83-97.

Ash J, Wu C, Larocque R, Jamal M, Stevens W, Osborne M, Thomas DY, Whiteway M. [Genetic analysis of the interface between Cdc42p and the CRIB domain of Ste20p in Saccharomyces cerevisiae.](http://www.ncbi.nlm.nih.gov/pubmed/12586692) *Genetics.* 2003; **163:** 9-20.

Oehlen LJ, Cross FR. [Potential regulation of Ste20 function by the Cln1-Cdc28 and Cln2-Cdc28 cyclin-dependent protein kinases.](http://www.ncbi.nlm.nih.gov/pubmed/9737966) *J Biol Chem.* 1998; **273:** 25089-97.

Drogen F, O'Rourke SM, Stucke VM, Jaquenoud M, Neiman AM, Peter M. [Phosphorylation of the MEKK Ste11p by the PAK-like kinase Ste20p is required for MAP kinase signaling in vivo.](http://www.ncbi.nlm.nih.gov/pubmed/10837245) *Curr Biol.* 2000; **10:** 630-9.

Whiteway MS, Wu C, Leeuw T, Clark K, Fourest-Lieuvin A, Thomas DY, Leberer E. [Association of the yeast pheromone response G protein beta gamma subunits with the MAP kinase scaffold Ste5p.](http://www.ncbi.nlm.nih.gov/pubmed/7667635) *Science.* 1995; **269:** 1572-5.

Wu C, Arcand M, Jansen G, Zhong M, Iouk T, Thomas DY, Meloche S, Whiteway M. [Phosphorylation of the MAPKKK regulator Ste50p in Saccharomyces cerevisiae: a casein kinase I phosphorylation site is required for proper mating function.](http://www.ncbi.nlm.nih.gov/pubmed/14555477) *Eukaryot Cell.* 2003; **2:** 949-61.

Neiman AM, Herskowitz I. [Reconstitution of a yeast protein kinase cascade in vitro: activation of the yeast MEK homologue STE7 by STE11.](http://www.ncbi.nlm.nih.gov/pubmed/8159759) *Proc Natl Acad Sci U S A.* 1994; **91:** 3398-402.

Zheng CF, Guan KL. [Activation of MEK family kinases requires phosphorylation of two conserved Ser/Thr residues.](http://www.ncbi.nlm.nih.gov/pubmed/8131746)*EMBO J.* 1994; **13:** 1123-31.

Ma D, Cook JG, Thorner J. [Phosphorylation and localization of Kss1, a MAP kinase of the Saccharomyces cerevisiae pheromone response pathway.](http://www.ncbi.nlm.nih.gov/pubmed/7579701) *Mol Biol Cell.* 1995; **6:** 889-909.

Roberts RL, Fink GR. [Elements of a single MAP kinase cascade in Saccharomyces cerevisiae mediate two developmental programs in the same cell type: mating and invasive growth.](http://www.ncbi.nlm.nih.gov/pubmed/8001818) *Genes Dev.* 1994; **8:** 2974-85.

Hagen DC, McCaffrey G, Sprague GF Jr. [Pheromone response elements are necessary and sufficient for basal and pheromone-induced transcription of the FUS1 gene of Saccharomyces cerevisiae.](http://www.ncbi.nlm.nih.gov/pubmed/1903837) *Mol Cell Biol.* 1991; **11:** 2952-61.

Hung W, Olson KA, Breitkreutz A, Sadowski I. [Characterization of the basal and pheromone-stimulated phosphorylation states of Ste12p.](http://www.ncbi.nlm.nih.gov/pubmed/9151949) *Eur J Biochem.* 1997; **245:** 241-51.

Feng Y, Davis NG. [Feedback phosphorylation of the yeast a-factor receptor requires activation of the downstream signaling pathway from G protein through mitogen-activated protein kinase.](http://www.ncbi.nlm.nih.gov/pubmed/10611235) *Mol Cell Biol.* 2000; **20:** 563-74.

Hicke L, Zanolari B, Riezman H. [Cytoplasmic tail phosphorylation of the alpha-factor receptor is required for its ubiquitination and internalization.](http://www.ncbi.nlm.nih.gov/pubmed/9548714) *J Cell Biol.* 1998; **141:** 349-58.

Roth AF, Davis NG. [Ubiquitination of the yeast a-factor receptor.](http://www.ncbi.nlm.nih.gov/pubmed/8707846) *J Cell Biol.* 1996; **134:** 661-74.

Roth AF, Davis NG. [Ubiquitination of the PEST-like endocytosis signal of the yeast a-factor receptor.](http://www.ncbi.nlm.nih.gov/pubmed/10713137) *J Biol Chem.* 2000;**275:** 8143-53.

Garrison TR, Zhang Y, Pausch M, Apanovitch D, Aebersold R, Dohlman HG. [Feedback phosphorylation of an RGS protein by MAP kinase in yeast.](http://www.ncbi.nlm.nih.gov/pubmed/10593933) *J Biol Chem.* 1999; **274:** 36387-91.

Parnell SC, Marotti LA Jr, Kiang L, Torres MP, Borchers CH, Dohlman HG. [Phosphorylation of the RGS protein Sst2 by the MAP kinase Fus3 and use of Sst2 as a model to analyze determinants of substrate sequence specificity.](http://www.ncbi.nlm.nih.gov/pubmed/15924435)*Biochemistry.* 2005; **44:** 8159-66.

Zhan XL, Guan KL. [A specific protein-protein interaction accounts for the in vivo substrate selectivity of Ptp3 towards the Fus3 MAP kinase.](http://www.ncbi.nlm.nih.gov/pubmed/10557209) *Genes Dev.* 1999; **13:** 2811-27.

Bardwell L. [A walk-through of the yeast mating pheromone response pathway.](http://www.ncbi.nlm.nih.gov/pubmed/15690603) *Peptides.* 2005; **26:** 339-50.

Jin M, Errede B, Behar M, Mather W, Nayak S, Hasty J, Dohlman HG, Elston TC. [Yeast dynamically modify their environment to achieve better mating efficiency.](http://www.ncbi.nlm.nih.gov/pubmed/21868361) *Sci Signal.* 2011; **4:** ra54.

Moore TI, Chou CS, Nie Q, Jeon NL, Yi TM. [Robust spatial sensing of mating pheromone gradients by yeast cells.](http://www.ncbi.nlm.nih.gov/pubmed/19052645) *PLoS One.* 2008; **3:** e3865.

Chant J, Herskowitz I. [Genetic control of bud site selection in yeast by a set of gene products that constitute a morphogenetic pathway.](http://www.ncbi.nlm.nih.gov/pubmed/2065354) *Cell.* 1991; **65:** 1203-12.

Bender A. [Genetic evidence for the roles of the bud-site-selection genes BUD5 and BUD2 in control of the Rsr1p (Bud1p) GTPase in yeast.](http://www.ncbi.nlm.nih.gov/pubmed/8234337) *Proc Natl Acad Sci U S A.* 1993; **90:** 9926-9.

Park HO, Chant J, Herskowitz I. [BUD2 encodes a GTPase-activating protein for Bud1/Rsr1 necessary for proper bud-site selection in yeast.](http://www.ncbi.nlm.nih.gov/pubmed/8371782) *Nature.* 1993; **365:** 269-74.

Wang HX, Konopka JB. [Identification of amino acids at two dimer interface regions of the alpha-factor receptor (Ste2).](http://www.ncbi.nlm.nih.gov/pubmed/19588927)*Biochemistry.* 2009; **48:** 7132-9.

Overton MC, Chinault SL, Blumer KJ. [Oligomerization of G-protein-coupled receptors: lessons from the yeast Saccharomyces cerevisiae.](http://www.ncbi.nlm.nih.gov/pubmed/16339714) *Eukaryot Cell.* 2005; **4:** 1963-70.

Gehret AU, Bajaj A, Naider F, Dumont ME. [Oligomerization of the yeast alpha-factor receptor: implications for dominant negative effects of mutant receptors.](http://www.ncbi.nlm.nih.gov/pubmed/16709573) *J Biol Chem.* 2006; **281:** 20698-714.

Overton MC, Blumer KJ. [G-protein-coupled receptors function as oligomers in vivo.](http://www.ncbi.nlm.nih.gov/pubmed/10744981) *Curr Biol.* 2000; **10:** 341-4.

Raicu V, Jansma DB, Miller RJ, Friesen JD. [Protein interaction quantified in vivo by spectrally resolved fluorescence resonance energy transfer.](http://www.ncbi.nlm.nih.gov/pubmed/15352875) *Biochem J.* 2005; **385:** 265-77.

Oehlen LJ, McKinney JD, Cross FR. [Ste12 and Mcm1 regulate cell cycle-dependent transcription of FAR1.](http://www.ncbi.nlm.nih.gov/pubmed/8649392) *Mol Cell Biol.*1996; **16:** 2830-7.

Rupp S, Summers E, Lo HJ, Madhani H, Fink G. [MAP kinase and cAMP filamentation signaling pathways converge on the unusually large promoter of the yeast FLO11 gene.](http://www.ncbi.nlm.nih.gov/pubmed/10064592) *EMBO J.* 1999; **18:** 1257-69.

Passmore S, Elble R, Tye BK. [A protein involved in minichromosome maintenance in yeast binds a transcriptional enhancer conserved in eukaryotes.](http://www.ncbi.nlm.nih.gov/pubmed/2673922) *Genes Dev.* 1989; **3:** 921-35.

White JM, Rose MD. [Yeast mating: getting close to membrane merger.](http://www.ncbi.nlm.nih.gov/pubmed/11166190) *Curr Biol.* 2001; **11:** R16-20.

Ren B, Robert F, Wyrick JJ, Aparicio O, Jennings EG, Simon I, Zeitlinger J, Schreiber J, Hannett N, Kanin E, Volkert TL, Wilson CJ, Bell SP, Young RA. [Genome-wide location and function of DNA binding proteins.](http://www.ncbi.nlm.nih.gov/pubmed/11125145) *Science.* 2000; **290:** 2306-9.

Dolan JW, Kirkman C, Fields S. [The yeast STE12 protein binds to the DNA sequence mediating pheromone induction.](http://www.ncbi.nlm.nih.gov/pubmed/2668945)*Proc Natl Acad Sci U S A.* 1989; **86:** 5703-7.

Errede B, Ammerer G. [STE12, a protein involved in cell-type-specific transcription and signal transduction in yeast, is part of protein-DNA complexes.](http://www.ncbi.nlm.nih.gov/pubmed/2558054) *Genes Dev.* 1989; **3:** 1349-61.

Fields S, Herskowitz I. [The yeast STE12 product is required for expression of two sets of cell-type specific genes.](http://www.ncbi.nlm.nih.gov/pubmed/3931921) *Cell.*1985; **42:** 923-30.

Hwang-Shum JJ, Hagen DC, Jarvis EE, Westby CA, Sprague GF Jr. [Relative contributions of MCM1 and STE12 to transcriptional activation of a- and alpha-specific genes from Saccharomyces cerevisiae.](http://www.ncbi.nlm.nih.gov/pubmed/1905781) *Mol Gen Genet.* 1991; **227:**197-204.

Bender A, Sprague GF Jr. [MAT alpha 1 protein, a yeast transcription activator, binds synergistically with a second protein to a set of cell-type-specific genes.](http://www.ncbi.nlm.nih.gov/pubmed/3304657) *Cell.* 1987; **50:** 681-91.

Jarvis EE, Clark KL, Sprague GF Jr. [The yeast transcription activator PRTF, a homolog of the mammalian serum response factor, is encoded by the MCM1 gene.](http://www.ncbi.nlm.nih.gov/pubmed/2550323) *Genes Dev.* 1989; **3:** 936-45.

Passmore S, Maine GT, Elble R, Christ C, Tye BK. [Saccharomyces cerevisiae protein involved in plasmid maintenance is necessary for mating of MAT alpha cells.](http://www.ncbi.nlm.nih.gov/pubmed/3066908) *J Mol Biol.* 1988; **204:** 593-606.

Tan S, Ammerer G, Richmond TJ. [Interactions of purified transcription factors: binding of yeast MAT alpha 1 and PRTF to cell type-specific, upstream activating sequences.](http://www.ncbi.nlm.nih.gov/pubmed/2854061) *EMBO J.* 1988; **7:** 4255-64.

Mahanty SK, Wang Y, Farley FW, Elion EA. [Nuclear shuttling of yeast scaffold Ste5 is required for its recruitment to the plasma membrane and activation of the mating MAPK cascade.](http://www.ncbi.nlm.nih.gov/pubmed/10481914) *Cell.* 1999; **98:** 501-12.

Garrenton LS, Braunwarth A, Irniger S, Hurt E, Künzler M, Thorner J. [Nucleus-specific and cell cycle-regulated degradation of mitogen-activated protein kinase scaffold protein Ste5 contributes to the control of signaling competence.](http://www.ncbi.nlm.nih.gov/pubmed/19001089) *Mol Cell Biol.* 2009; **29:** 582-601.

Bardwell L, Cook JG, Zhu-Shimoni JX, Voora D, Thorner J. [Differential regulation of transcription: repression by unactivated mitogen-activated protein kinase Kss1 requires the Dig1 and Dig2 proteins.](http://www.ncbi.nlm.nih.gov/pubmed/9860980) *Proc Natl Acad Sci U S A.*1998; **95:** 15400-5.

Leslie DM, Grill B, Rout MP, Wozniak RW, Aitchison JD. [Kap121p-mediated nuclear import is required for mating and cellular differentiation in yeast.](http://www.ncbi.nlm.nih.gov/pubmed/11909949) *Mol Cell Biol.* 2002; **22:** 2544-55.

Blondel M, Alepuz PM, Huang LS, Shaham S, Ammerer G, Peter M. [Nuclear export of Far1p in response to pheromones requires the export receptor Msn5p/Ste21p.](http://www.ncbi.nlm.nih.gov/pubmed/10485850) *Genes Dev.* 1999; **13:** 2284-300.

Nern A, Arkowitz RA. [Nucleocytoplasmic shuttling of the Cdc42p exchange factor Cdc24p.](http://www.ncbi.nlm.nih.gov/pubmed/10725324) *J Cell Biol.* 2000; **148:** 1115-22.

Marín MJ, Flández M, Bermejo C, Arroyo J, Martín H, Molina M. [Different modulation of the outputs of yeast MAPK-mediated pathways by distinct stimuli and isoforms of the dual-specificity phosphatase Msg5.](http://www.ncbi.nlm.nih.gov/pubmed/19123063) *Mol Genet Genomics.*2009; **281:** 345-59.

Yang HY, Tatebayashi K, Yamamoto K, Saito H. [Glycosylation defects activate filamentous growth Kss1 MAPK and inhibit osmoregulatory Hog1 MAPK.](http://www.ncbi.nlm.nih.gov/pubmed/19369942) *EMBO J.* 2009; **28:** 1380-91.

Singh A, Chen EY, Lugovoy JM, Chang CN, Hitzeman RA, Seeburg PH. [Saccharomyces cerevisiae contains two discrete genes coding for the alpha-factor pheromone.](http://www.ncbi.nlm.nih.gov/pubmed/6306574) *Nucleic Acids Res.* 1983; **11:** 4049-63.

Cappellaro C, Baldermann C, Rachel R, Tanner W. [Mating type-specific cell-cell recognition of Saccharomyces cerevisiae: cell wall attachment and active sites of a- and alpha-agglutinin.](http://www.ncbi.nlm.nih.gov/pubmed/7957044) *EMBO J.* 1994; **13:** 4737-44.

Michaelis S, Herskowitz I. [The a-factor pheromone of Saccharomyces cerevisiae is essential for mating.](http://www.ncbi.nlm.nih.gov/pubmed/3285180) *Mol Cell Biol.*1988; **8:** 1309-18.

Trueheart J, Boeke JD, Fink GR. [Two genes required for cell fusion during yeast conjugation: evidence for a pheromone-induced surface protein.](http://www.ncbi.nlm.nih.gov/pubmed/3302672) *Mol Cell Biol.* 1987; **7:** 2316-28.

Erdman S, Lin L, Malczynski M, Snyder M. [Pheromone-regulated genes required for yeast mating differentiation.](http://www.ncbi.nlm.nih.gov/pubmed/9456310) *J Cell Biol.* 1998; **140:** 461-83.

Roy A, Lu CF, Marykwas DL, Lipke PN, Kurjan J. [The AGA1 product is involved in cell surface attachment of the Saccharomyces cerevisiae cell adhesion glycoprotein a-agglutinin.](http://www.ncbi.nlm.nih.gov/pubmed/2072914) *Mol Cell Biol.* 1991; **11:** 4196-206.

McGrath JP, Varshavsky A. [The yeast STE6 gene encodes a homologue of the mammalian multidrug resistance P-glycoprotein.](http://www.ncbi.nlm.nih.gov/pubmed/2569166) *Nature.* 1989; **340:** 400-4.

Kuchler K, Sterne RE, Thorner J. [Saccharomyces cerevisiae STE6 gene product: a novel pathway for protein export in eukaryotic cells.](http://www.ncbi.nlm.nih.gov/pubmed/2686977) *EMBO J.* 1989; **8:** 3973-84.

Fujimura H. [Molecular cloning of the DAC2/FUS3 gene essential for pheromone-induced G1-arrest of the cell cycle in Saccharomyces cerevisiae.](http://www.ncbi.nlm.nih.gov/pubmed/2078866) *Curr Genet.* 1990; **18:** 395-400.
