# Supplementary figures and images for: Network analyses based on comprehensive molecular interaction maps reveal robust control structures in yeast stress response pathways
Source: NPJ Syst Biol Appl. 2016 Jan 7;2:15018–. doi: 10.1038/npjsba.2015.18 (PMC5516916; doi:10.1038/npjsba.2015.18)

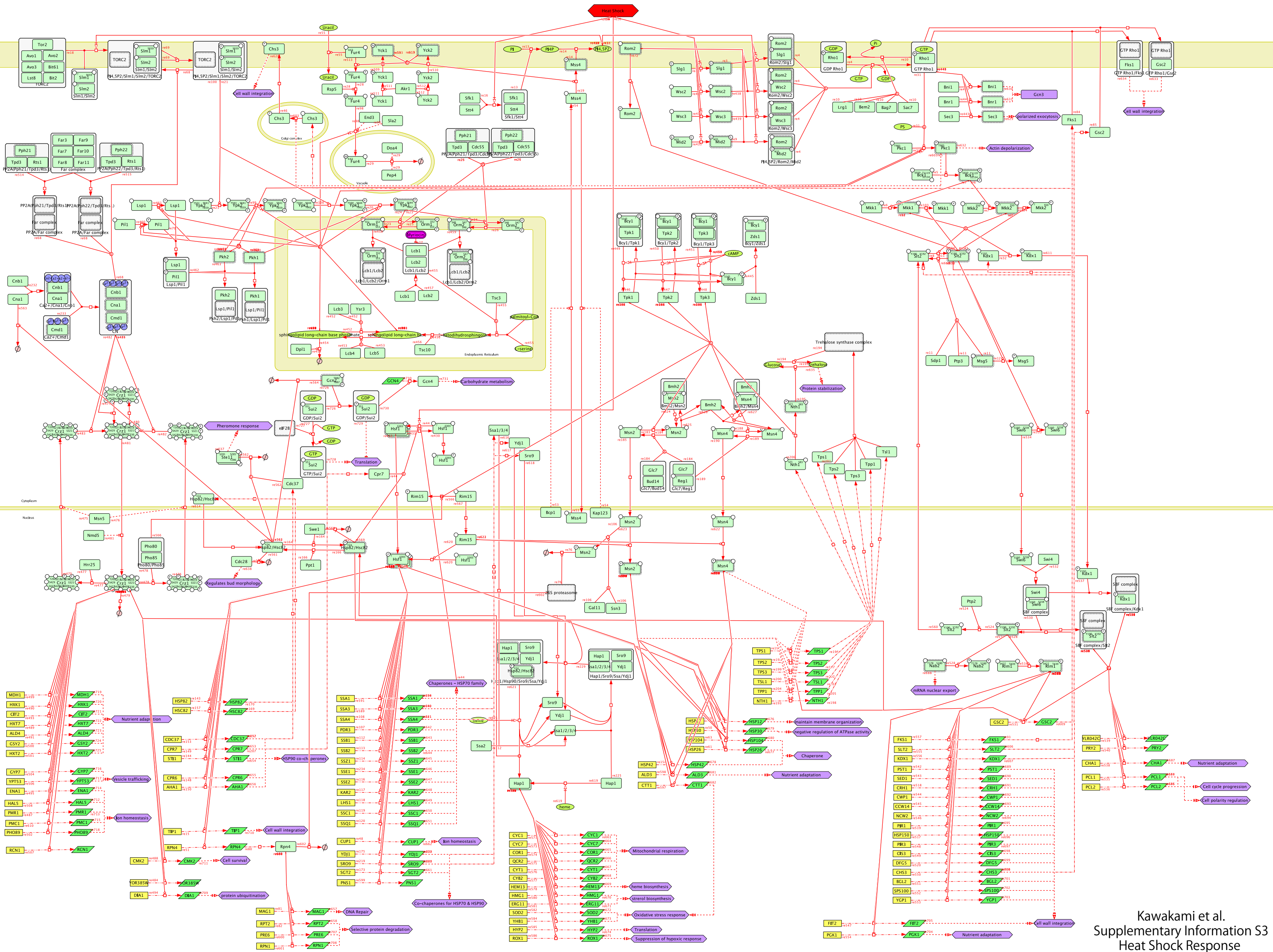

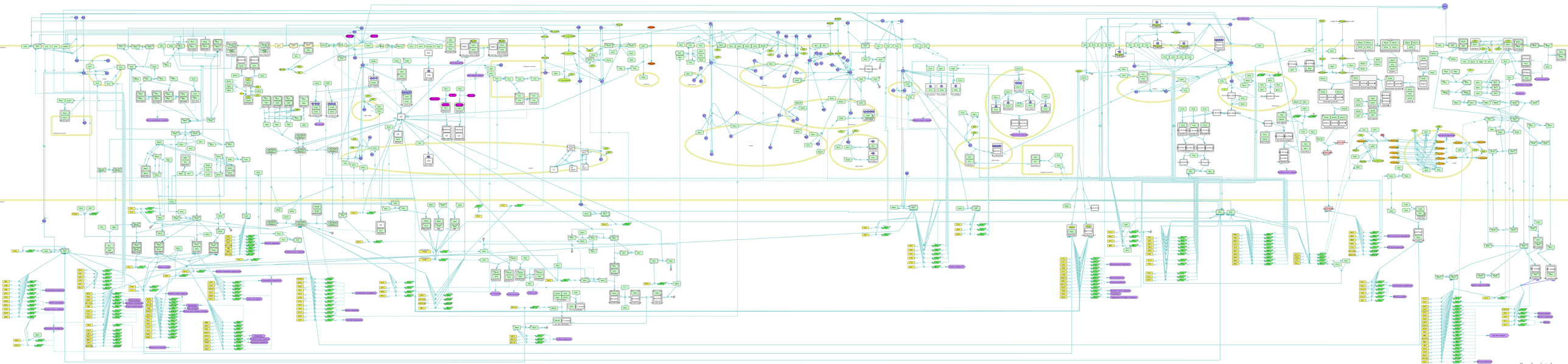

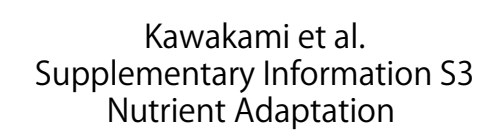

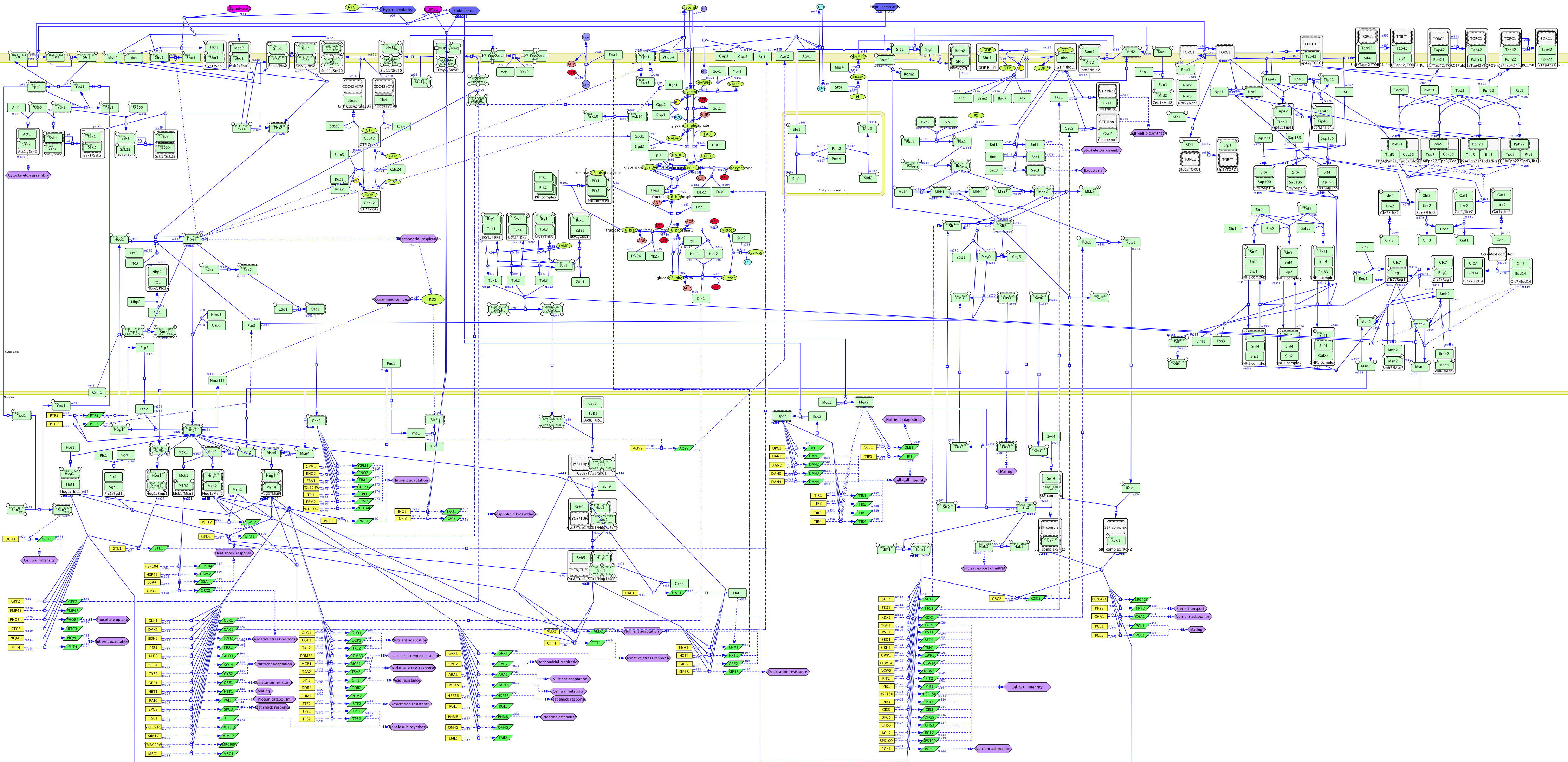

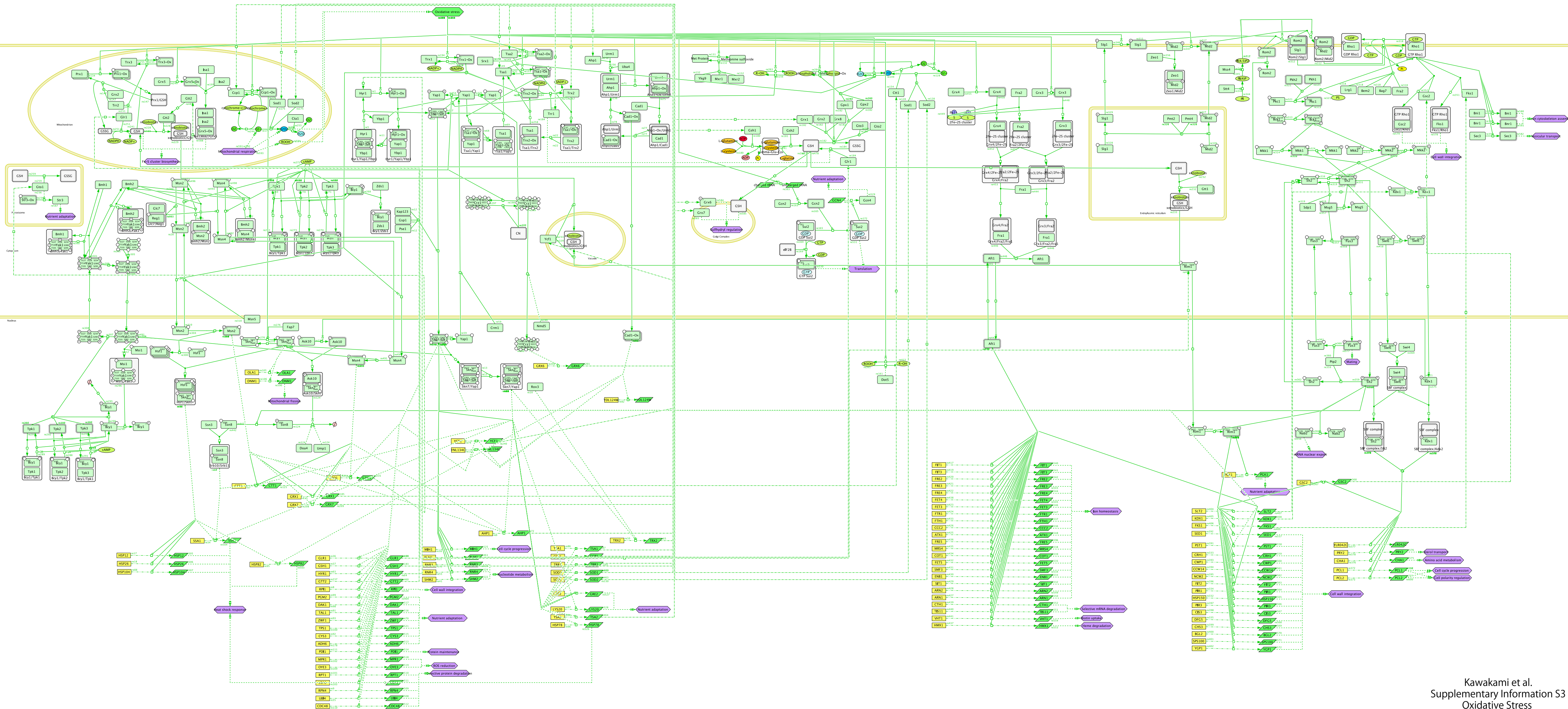

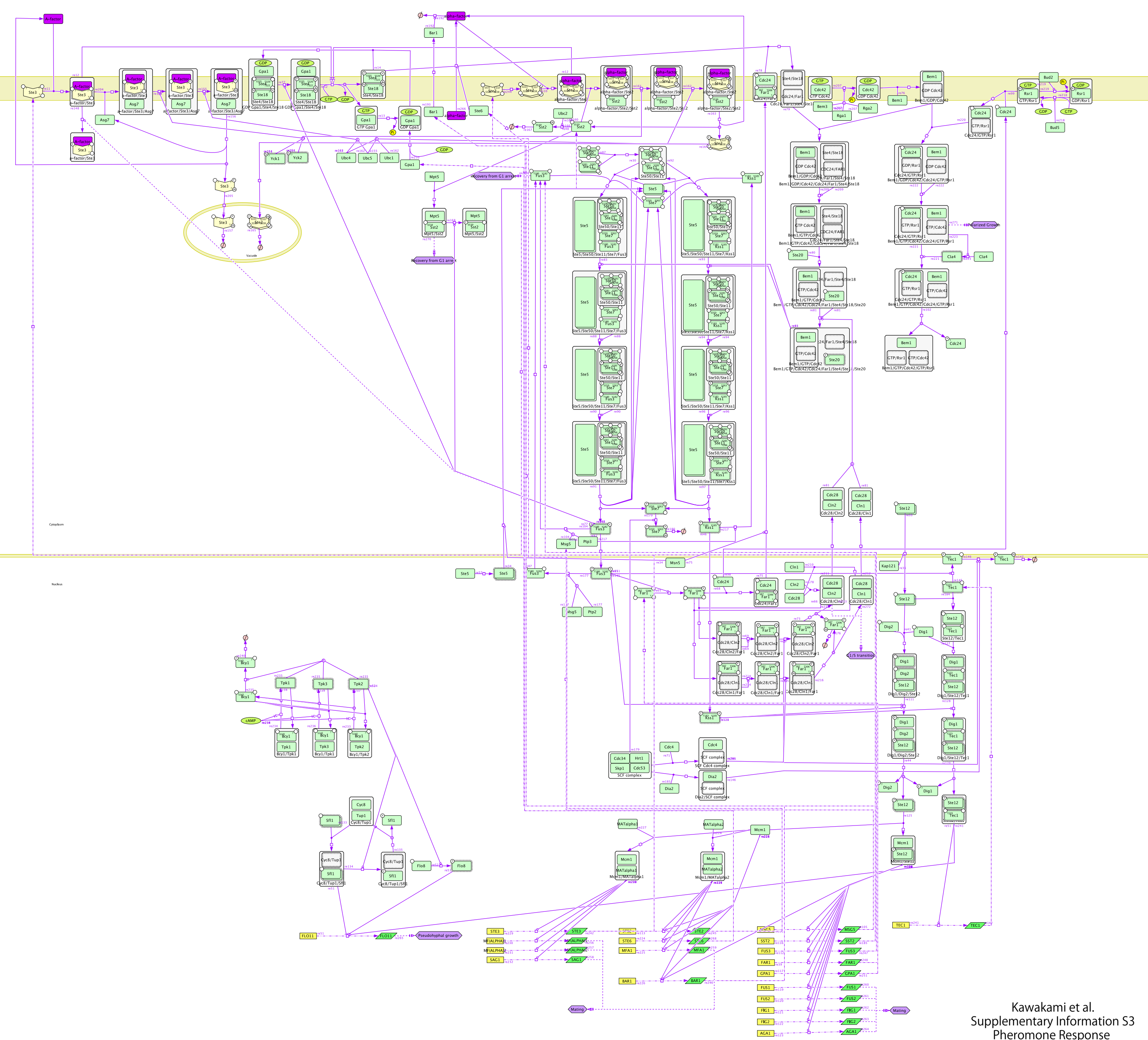

Supplement: Supplementary Information S3 [file npjsba201518-s3.pdf]

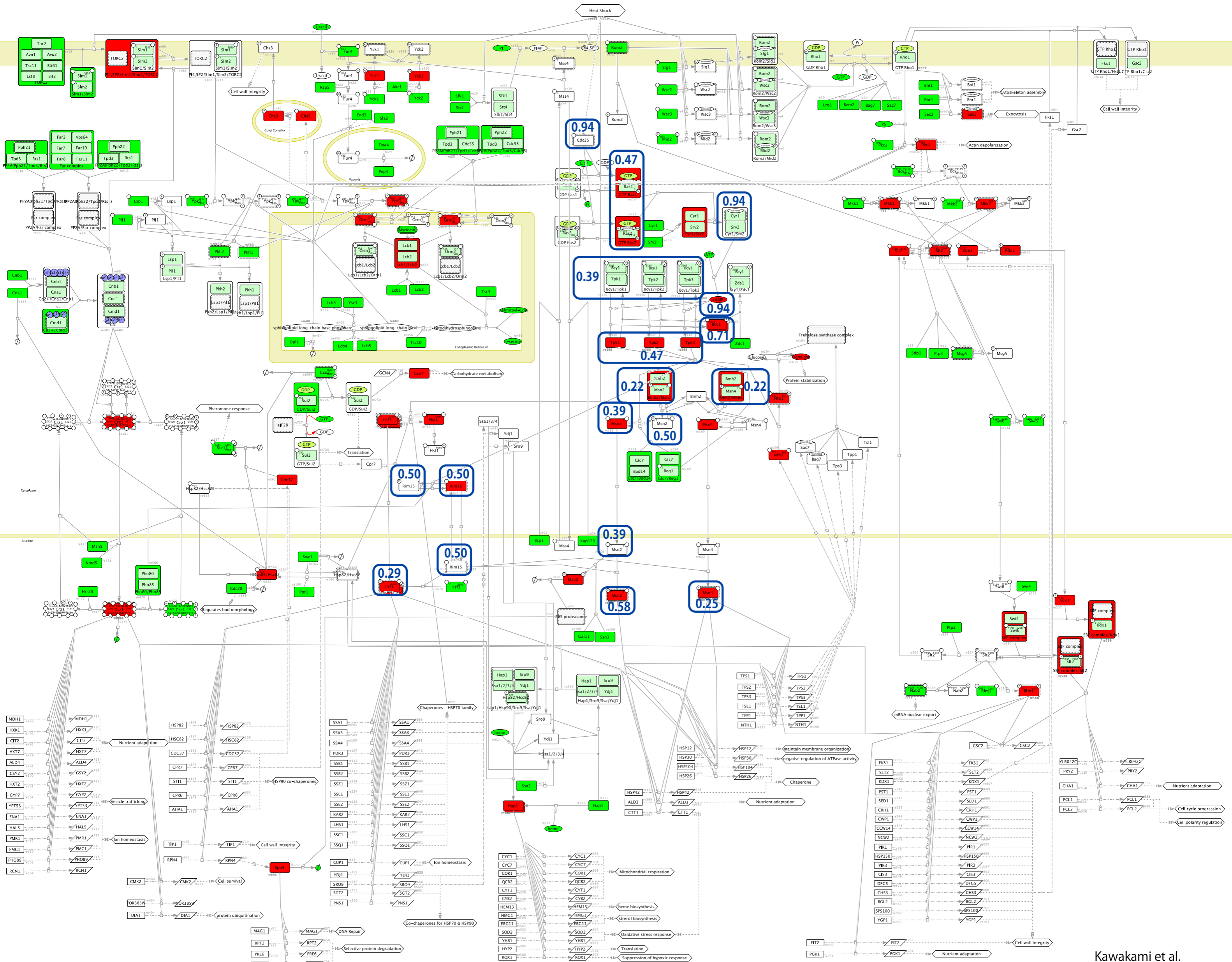

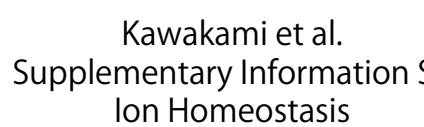

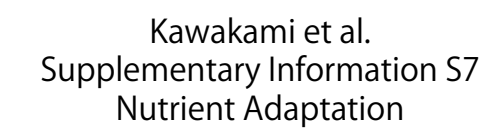

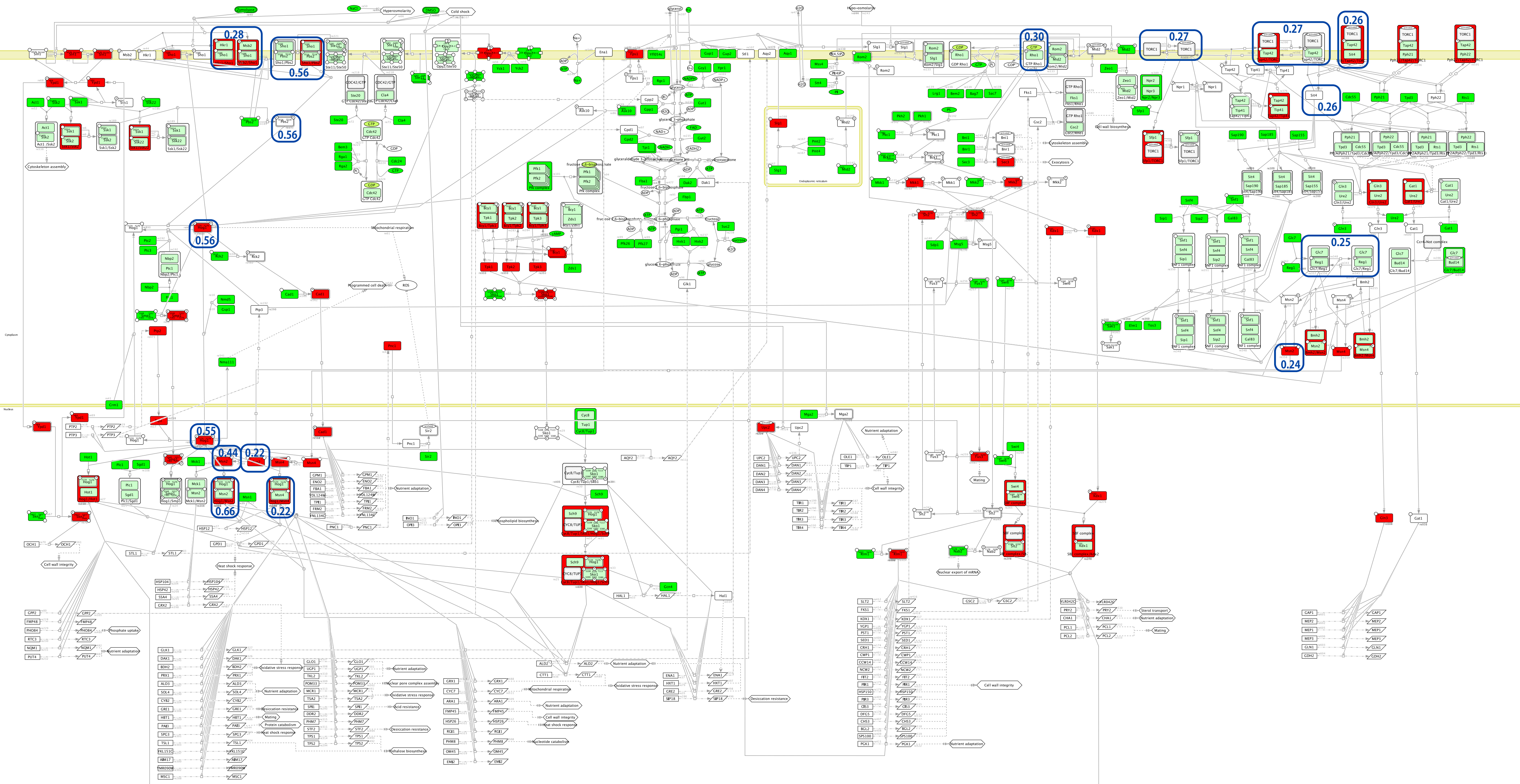

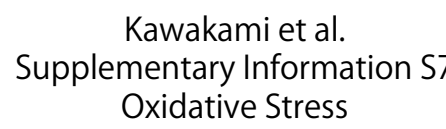

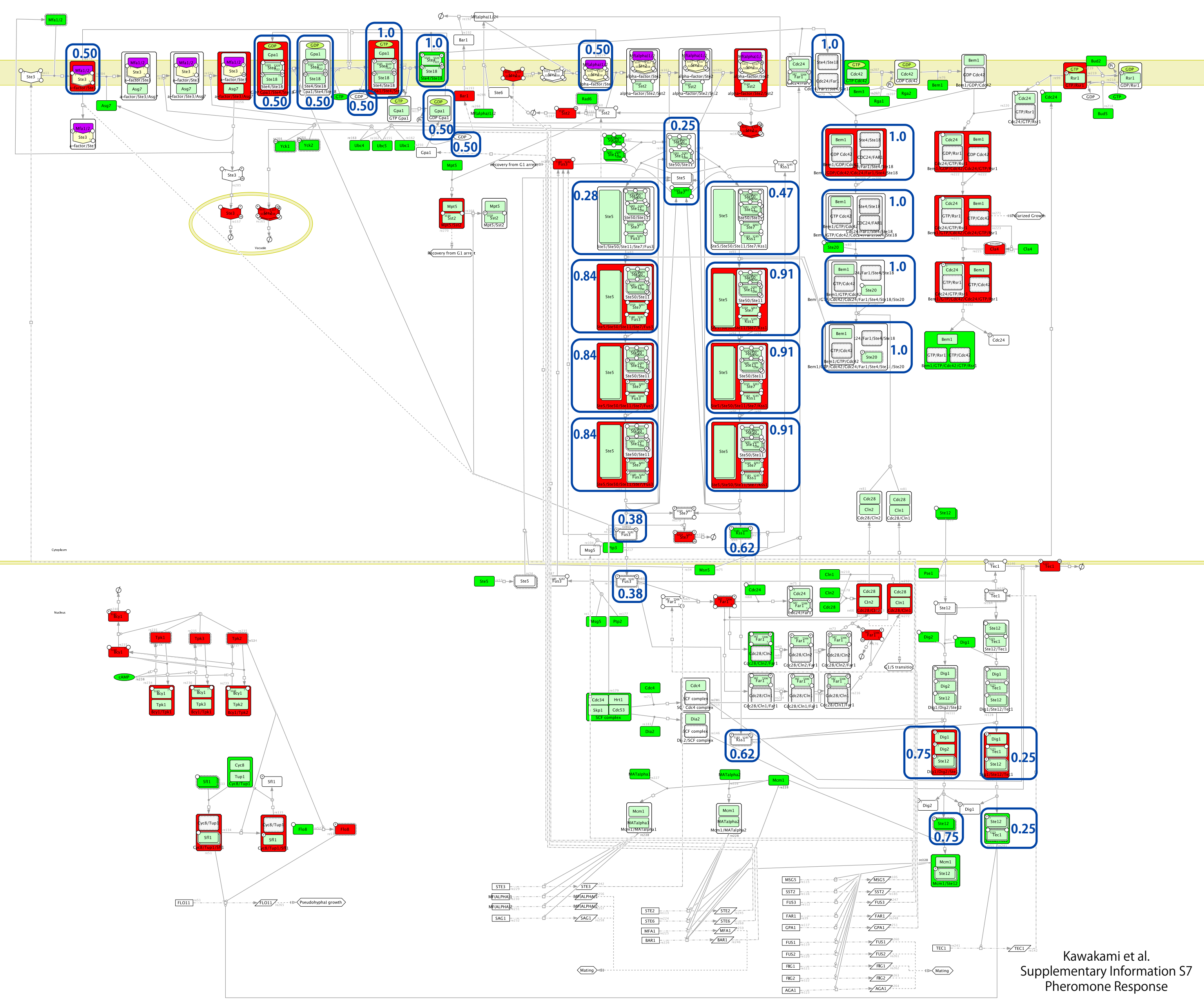

Supplement: Supplementary Information S7 [file npjsba201518-s7.pdf]
