# Supplementary material for: Network analyses based on comprehensive molecular interaction maps reveal robust control structures in yeast stress response pathways
Source: NPJ Syst Biol Appl. 2016 Jan 7;2:15018–. doi: 10.1038/npjsba.2015.18 (PMC5516916; doi:10.1038/npjsba.2015.18)

Comprehensive Molecular Interaction Map of Yeast Stress Response Ver. 1.0

RIKEN IMS Laboratory for Disease Systems Modeling,
The Systems Biology Institute, Persistent Systems

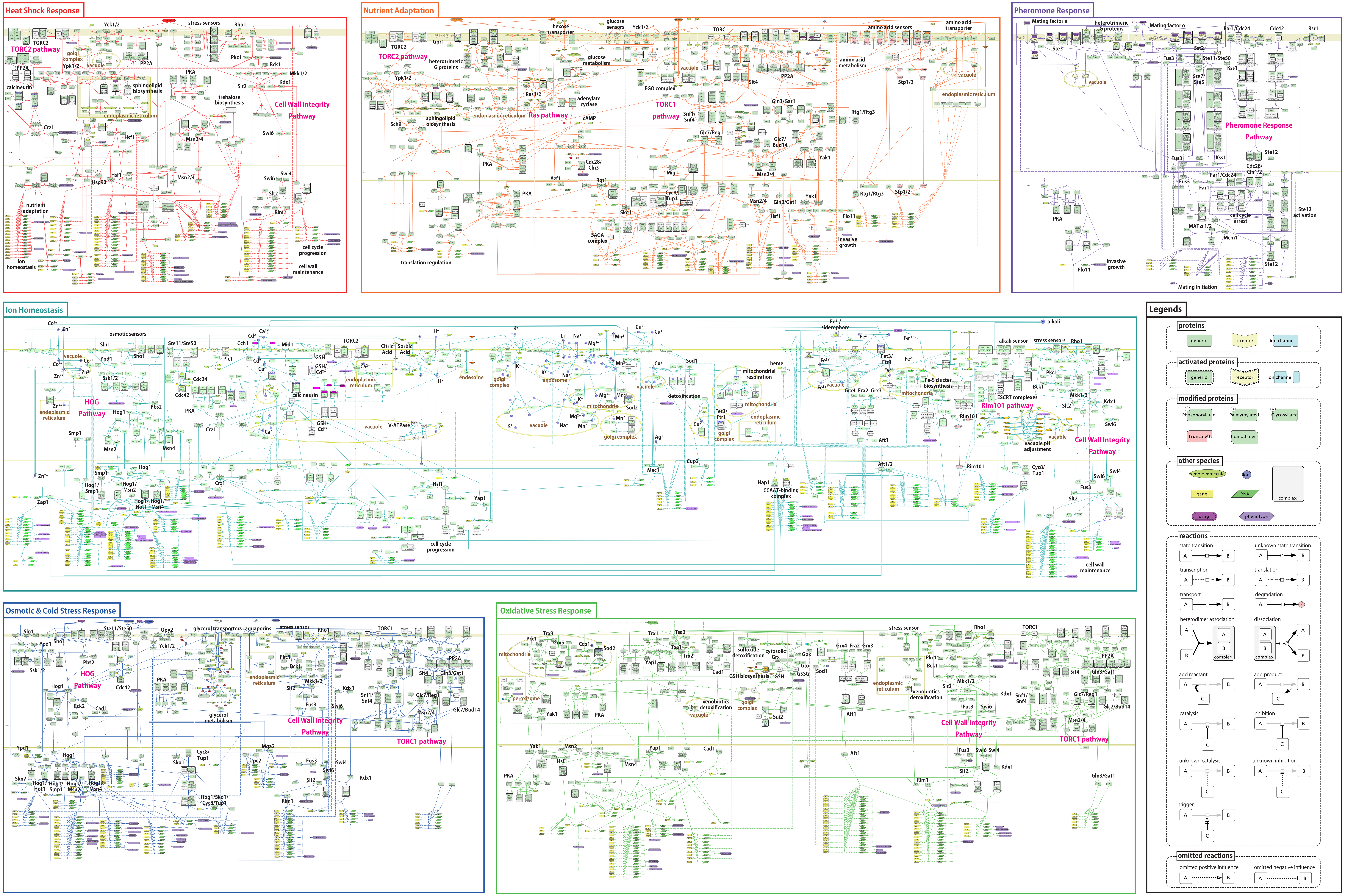

Supplement: Supplementary Information S4 [file npjsba201518-s4.pdf]
