# Supplementary material for: Network analyses based on comprehensive molecular interaction maps reveal robust control structures in yeast stress response pathways
Source: NPJ Syst Biol Appl. 2016 Jan 7;2:15018–. doi: 10.1038/npjsba.2015.18 (PMC5516916; doi:10.1038/npjsba.2015.18)
Supplement: Supplementary Information S5 [file npjsba201518-s5.doc]

**Supplementary Information S5**

Biological features of 6 stress response maps.

*Response to Heat Shock*

Variation in temperature is probably the most common environmental stress for all organisms. Yeasts can cope with high temperatures up to 42°C employing a series of protective reactions including chaperone induction and trehalose biosynthesis. The heat shock transcription factor (Hsf1) activates most of transcription of heat shock genes. Hsf1 is extensively phosphorylated and activated with uncharacterized mechanism. One possibility is that misfolded proteins trigger Hsf1 activation. Hsf1 target genes attenuate expression within 20-40 min after induction. The transient activation of heat shock response can be explained by a negative feedback loop in which heat shock proteins including Hsp70 and Hsp90 complex (Hsp82/Hsc82) repress Hsf1 activity. In addition to activation of Hsf1, cell wall integrity (CWI) signaling is also induced in response to heat shock. Increased production of PI(4, 5)P2 activates Rho1 which in turn controls cell wall structural component genes via MAP kinase (MAPK) cascade. Slt2, a MAPK responsible for the CWI signaling pathway, phosphorylates and activates the transcription factor Rlm1 that regulates expression of cell wall integrity related genes. Yeast has a pseudokinase paralog of Slt2, named Kdx1. Although Kdx1 has no catalytic function as a kinase, Kdx1 has shared role with Slt2 in forming a complex with Swi4 and activating SBF complex. SBF complex binds to the Swi4/6 cell cycle box (SCB) promoter element and activates transcription duringthe G1/S transition of the cell cycle. TORC2 pathway is also activated by PI(4, 5)P2 inducing sphingolipid long-chain base biosynthesis. Other major players in heat shock response are Msn2/4, which are required for expression of a wide array of genes in multiple stresses. Under heat stress, genes composing trehalose synthetic complex as well as some chaperone genes are induced by Msn2/4 under control of cAMP/PKA.

*Osmotic and Cold Stress Response*

Osmotic changes are also lethal for the unicellular yeast cells. Any water-soluble compounds including ions and sugar increase external osmolarity, while too much water causes hypo-osmolarity. Yeast counteracts the sudden osmotic changes by controlling concentration of cytosolic ions (especially Na+), water efflux and influx, glycerol metabolism and cell wall integrity. Cold stress and dimethyl sulfoxide (DMSO) also leads to a reduction in membrane fluidity inducing cellular responses similar to that of hyper-osmotic stress. High osmolarity glycerol (HOG) pathway predominantly mediates the transduction of hyper-osmotic and cold shock signal. The upstream part of HOG pathway comprises two distinct branches, namely Sho1 and Sln1 branches. Both branches activate a common MAPK kinase (MAPKK), Pbs2, which is the specific activator of the Hog1 MAPK. Activated Hog1 is rapidly imported into the nucleus and regulates various transcription factors including Msn2/4, Hot1, Sko1, Smp1. Cold shock independently stimulates the induction of several genes involved in phospholipid biosynthesis. On the other hand, under the hypo-osmotic stress, the cell tries to maintain cellular turgor inducing CWI signaling via Slg1 and suppressing HOG pathway via Sln1 branch.

*Ion homeostasis*

Ionic stress in yeast may be caused due to several entities such as alkali, Na^+^, K^+^, Li^+^, Ca^2+^, Mg^2+^, Mn^2+^, Cu^2+^, Cd^2+^, Zn^2+^, Co^2+^, Fe^3+^, and organic acids including citric acid and sorbic acid. Ions have crucial roles to play in the maintenance of yeast cells. However, when they exceed their permissible non-lethal limit, they are deemed as stress-causing agents. Yeast cells employ ion homeostasis pathways to ensure that a balance between their ionic requirement and the detoxification pathways is maintained. In particular, Zn^2+^ function as a catalytic cofactor as in the active site of an enzyme or in a zinc finger. The zinc-sensing transcription factor Zap1 is mainly responsible for the control of the response to zinc deficiency in yeast. In addition to direct activation of Zap1 activity, severe zinc deficiency increases the expression level of Zap1 through autoactivation at the ZAP1 promoter which itself contains Zap1 binding DNA sequence. Intracellular zinc level is regulated by Zap1 target zinc transporters that serve to transfer zinc between the extracellular milieu, the vacuolar lumen, the endoplasmic reticulum and the cytosol. Some transporters involved in zinc transfer also play a role in cobalt accumulation. Ca^2+^ works widely as a second messenger in prokaryotic and eukaryotic cells. In yeast, the most characterized target of Ca^2+^ is calmodulin (Cmd1), which is activated by Ca^2+^ and binds to and activates protein kinases and calcineurin. Calcineurin is a phosphatase which dephosphorylates a range of protein including the transcription factor Crz1, TORC2 effectors (Slm1 and Slm2), the trafficking adaptor Aly1, and possibly the calcium importers (Mid1 and Cch1). Ca^2+^ also stimulates HOG pathway via two osmosensors (Sln1, Sho1). In yeast, slightly different from mammalian cells, most of Ca^2+^ is stored in the vacuolar lumen, but less in the endoplasmic reticulum or in the mitochondria. Cadmium ion influx is also mediated by the Mid1/Cch1 plasma membrane channel. However Cd^2+^ is toxic for biological system and forms complexes with reduced glutathione (GSH). The Cd^2+^-GSH complexes can be removed from the cytosol and transported into the vacuole. The cytosolic concentration of protons, expressed as pH, is tightly regulated by the activity of two proton pumps Pma1 and V-ATPase complex. Pma1 is a P2-type ATPase which pumps proton across the plasma membrane, which is activated by low pH and addition of glucose. V-ATPase is the vacuolar proton-translocating ATPase, which pumps protons from the cytosol into the vacuole. V-ATPase is regulated through the reversible dissociation/association of its two components (V_0_ and V_1_ subcomplex). This phenomenon is observed during glucose starvation and requires a number of different regulatory proteins including the RAVE complex, PKA/Ras activity, although detailed mechanism has not been elucidated. In addition to Pma1 and V-ATPase, the nutrient permeases for sugars and aminoacids, together with the K+ transporters and the Na+/H+ antiporter, influence cytosolic pH consuming the proton gradient across the plasma membrane. Sodium and potassium are the family of alkali metals. In cells, K^+^ plays various physiological roles, whereas Na^+^ is rather toxic at high concentration by substituting for K^+^. Therefore, yeast maintains a high intracellular ratio of K^+^/Na^+^ by the selective accumulation of K^+^ and the active extrusion of Na^+^. The high affinity K^+^ transporter Trk1 and Trk2 are responsible for K^+^ uptake regulated by several protein kinases and phosphatases. The P-type ATPases and Nha1 antiporter promote efflux of Na^+^, Li^+^, and to a lesser extent, K^+^. Magnesium serves as an essencial cofactor for many enzymes. Mg^2+^ is usually stored in the vacuole and mitochondria, but the responsible Mg^2+^/H^+^ exchanger has not been identified yet. Under Mg^2+^-deficient conditions, Mg^2+^ is released into the cytoplasm by the function of Mnr2. Manganese is a redox-active metal found in the superoxide dismutase (Sod2) of the mitochondria and in the sugar transferases of the secretory pathway. Manganese is imported into the cytoplasm by the Nramp family transporter Smf1 and by the MnHPO_4_ transporter Pho84. In magnesium rich conditions, Pho84 also mediates the MgHPO_4_ import. Copper ions participate in redox reactions and mitochondrial respiration process. Therefore copper uptake and utilization are tightly regulated by copper-sensing transcriptional activators, Mac1 and Ace1. Mac1 can sense two different levels of copper ions. In other words, the Mac1 bind DNA in Cu^+^-dependent manner, but excess Cu^+^ disrupts the DNA binding of Mac1. Extracellular copper is largely present in the oxidized state, Cu^2+^, so it undergoes reduction to Cu^+^ before uptake. When extracellular copper concentrations rise, copper-binding proteins called metallothioneins (Cup1 and Crs5) are expressed under control of Ace1, which bind and detoxify cytosolic copper. Iron homeostasis is largely controlled by the iron-dependent transcriptional activators, Aft1 and Aft2. Under iron deficiency, Aft1 accumulates in the nucleus and binds DNA to activate transcription. When intracellular iron level is increased, Aft1 is exported from nucleus and inactivated by iron-sulfur cluster (ISC)-mediated process. There are two genetically separate systems of iron uptake. One is the reductive system, in which the Fe^3+^ ion is reduced to the Fe^2+^ ion before uptake. In another system, yeast utilize siderophores to chelates Fe^3+^, which produced by other species of fungi and bacteria. The siderophore-iron chelates are imported into the cytoplasm by the ARN/SIT family transporters. Alkalization activates the Rim101 pathway in addition to CWI pathway. In Rim101 pathway, external alkalization perceived by integral membrane proteins leads to proteolytic activation of Rim101. ESCRT proteins, originally reported to be required for vacuolar protein sorting, are necessary for this process. The activated Rim101 translocates to the nucleus and induces alkaline response genes indirectly through repressing expression of transcription repressors such as Nrg1 and Smp1. Hence, by modulating the activity of the transporters (importers and exporters) that are highly specific for different ions, yeast ensures that ion homeostasis is maintained within the cell.

*Oxidative stress*

Yeast induces the expression of limited number of antioxidants that sufficiently protect it against reactive oxygen species (ROS). ROS is naturally generated from internal aerobic metabolism as well as from environment. The onset of oxidative stress generally induces an early response in yeast cells wherein the pre-existing antioxidant defense provides immediate protection against the initial sub-lethal accumulation of ROS. Catalases (Cta1 in the peroxisome/mitochondria and Ctt1 in the cytosol) catalyze the dismutation of H_2_O_2_ into H_2_O and O_2_. Superoxide dismutases (SODs) are another antioxidant family proteins that convert the superoxide anion to hydrogen peroxide. Yeast contains a cytoplasmic Cu, Zn-SOD (Sod1) and a mitochondrial matrix Mn-SOD (Sod2). Methionine residues that are particularly susceptible to oxidation by ROS should be reduced by methionine sulfoxide reductases (MSRs). Ykl069w, Mxr1 and Mxr2 are MSR enzymes in yeast. Yeast also contains a cytoplasmic thioredoxin system comprising two thioredoxins (Trx1 and Trx2) and a thioredoxin reductase (Trr1) and a mitochondrial thioredoxin system comprising a thioredoxin (Trx3) and a thioredoxin reductase (Trr2). In the cytoplasmic thioredoxin system, the thioredoxins receive oxidation from peroxiredoxin (Tsa1) and are reduced directly by NADPH and Trr1. Similarly, Trx3 is reduced by NADPH and Trr2, although the oxidation donor to Trx3 has not been identified. GSH (γ-glutamylcysteinylglycine) is the most abundant low molecular-weight sulfhydryl compound playing a role in xenobiotics and ROS detoxification. In protection against ROS, oxidative stress as well as GSH-dependent reductase including glutaredoxin (Grx), glutathione peroxidase (Gpx), and omega-class glutathione transferase (Gto) convert GSH to its oxidized disulfide form (GSSG). Glutathione is predominantly present in its reduced GSH form by the constitutive activity of glutathione reductase (Glr1). The constitutive defense against ordinary level of ROS is incapable of protecting the cell from massive and sudden oxidative insults caused due to chemicals such as hydrogen peroxide (H_2_O_2_). Consequently, the early stress signals are passed downstream which initiates the late response. The late response involves activation of transcription factors Yap1 and Cad1 that are responsible for the synthesis of new antioxidant molecules. Tsa1 and Hyr1 activate Yap1 forming intramolecular disulfide bonds, while Ahp1 activates Cad1 in the same manner. Activated Yap1 and Cad1 is imported into the nucleus and activates gene expression of antioxidant proteins.

*Nutrient Adaptation*

In free-living micro-organisms, nutrient availability is the major factor controlling growth and development. In yeast cells, glucose is the favored source of carbon and serves as an important primary messenger molecule. Glucose availability triggers optimal conditions for processes such as growth, metabolism and proliferation. Plasma membrane proteins highly similar to the Hxt glucose transporters (Snf3 and Rgt2) act as receptors that sense external glucose. Then, Snf3 and Rgt2 are suggested to activate the type I casein kinases Yck1 and Yck2. Mth1 and Std1, which interact with Rgt1 and suppress gene expression of Hxt glucose transporters, are phosphorylated by Yck1/Yck2 followed by the ubiquitination by the SCF complex and the degradation via the proteasome. Thus, glucose induces Rgt1 activation and consequent induction of HXT genes. Alternatively, Yck1/Yck2 phosphorylate and activate Mss4 which catalyzes the conversion of PI(4)P to PI(4, 5)P2 at the plasma membrane. PI(4, 5)P2 activates TORC2 pathway which leads to the production of sphingolipid long-chain base and the activation of Sch9. Sch9 regulates transcription factors involved in ribosome biogenesis and translation initiation. Other possible glucose target protein is Gpr1, a plasma membrane protein coupled with the G protein Gpa2. Activated Gpa2 by Gpr1 in turn stimulates adenylyl cyclase to elevate the cellular levels of cAMP. Finally, binding of cAMP to Bcy1 causes dissociation of the Tpk1/2/3-Bcy1 complex inducing PKA activity. PKA is responsible for various protein phosphorylation including transcription factors, metabolic enzymes, and other regulatory kinases. Gpb1 and Gpb2 negatively regulate the activity of the Ras/PKA pathway by interference with the Gpr1/Gpa2 interaction, or thorough stabilization of the Ras-GAP proteins, Ira1 and Ira2, or by stabilization of the Tpk1/2/3-Bcy1 complex. Another nutrient essential for yeast survival is nitrogen. Two major pathways mediate the response of yeast to nitrogen availability. One of them is TORC1 pathway, in which EGO complex senses intracellular amino acids and activates TORC1 complex. TORC1 transfers the signal to two major branches: Sch9 and Tap42-PPase complexes. TORC1 phosphorylates and activates Sch9 stimulating translation as well as in glucose sensing pathway, while Tap42 phosphorylated by TORC1 associates with PPase phosphatase complexes and resides in membrane. Rapamycin, TORC1 inhibitor, treatment or nitrogen starvation releases Tap42-PPase to the cytosol. Released phosphatases dephosphorylate various target including protein kinease Snf1 and transcription factors Gln3 and Gat1 inducing gene expression to deal with nitrogen starvation. Extracellular amino acid is perceived by Ssy1-Ptr3 pathway which induces expression of amino acid transporter genes.

*Pheromone Response*

Yeast cell exists either as haploid or diploid and can reproduce by sexual or asexual means. Sexual reproduction involves the mating pheromone response that is a characteristic feature of haploid cells. These haploid yeasts have two mating genotypes, namely MATa and MATα. The mating response to generate a diploid cell is stimulated by the release of small peptide mating pheromones, alpha-factor from MATα cells and a-factor from MATa cells, which act on cells of the opposite mating type to prepare them for mating. The cellular response to mating pheromone includes the polarized growth of one haploid cell toward its mating partner, cell cycle arrest in G1 phase and increased expression of proteins that are needed for cell adhesion, cell fusion and nuclear fusion. Mating pheromone is sensed by G-protein-coupled receptors (GPCRs) on the cell surface, Ste2 for alpha-factor and Ste3 for a-factor, respectively. As other GPCRs, the receptor bound by its ligand promotes GTP binding to the Gα subunit (Gpa1) of the G protein. GTP-bound Gpa1 then releases the Gβγheterodimer (Ste4, Ste18). After released from Gpa1, Ste4/Ste18 binds to multiple effectors including a Ste5/Ste11 complex, the Ste20 protein kinase and a Far1/Cdc24 complex to bring them near each other. Brought near together, Ste20 is activated by autophosphorylation and then phosphorylates and activates Ste11 MAPKKK. Using Ste5 as a binding platform, Ste11 activates Ste7 MAPKK by phosphorylating its activation loop and Ste7, in turn, activates two MAPKs, Kss1 and Fus3. The major intranuclear targets of Fus3 and Kss1 are Ste12/Dig1/Dig2 transcription factor complex and Far1. For Ste12/Dig1/Dig2 complex, Fus3 and Kss1 phosphorylate all of the components and decrease the ability of Dig1/2 to bind to and repress Ste12. Released Ste12 binds to a DNA promoter and induce expression of genes including components of the mating pathway (Ste2, Fus3 and Far1 as positively-acting factors and Sst2, Msg5 and Gpa1 as negatively-acting factors) and cell fusion related factors. Far1 acts as a multifunctional regulator of the mating process binding to Cdc24 and Cdc28. Far1 binding of Cdc24 constitute a positive feedback loop in pheromone response pathway, while association with Cdc28 inhibits Cdc28 function as the master regulator of yeast cell cycle leading to pheromone-induced cell cycle arrest. Additionally, Fus3 and Kss1 phosphorylate several upstream components of the pathway including Ste5, Ste11 and Ste7.
