# Supplementary material for: Network analyses based on comprehensive molecular interaction maps reveal robust control structures in yeast stress response pathways
Source: NPJ Syst Biol Appl. 2016 Jan 7;2:15018–. doi: 10.1038/npjsba.2015.18 (PMC5516916; doi:10.1038/npjsba.2015.18)
Supplement: Supplementary Information S10 [file npjsba201518-s15.doc]

**Supplementary Information S10**. Motifs common in the 6 yeast stress response maps.

| Motif | Frequency in yeast stress response pathways | | | | | | Appearance in previously reported pathways | |
| --- | --- | --- | --- | --- | --- | --- | --- | --- |
|  | Ion  Homeostasis | Nutrient  Adaptation | Osmotic  Stress | Oxidative  Stress | Heat  Shock | Pheromone  Response | stimuli  response^a^ | Others^b^ |
| 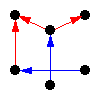 | 637 | 107 | 112 | 65 | 36 | 53 | 2/3 | 1/2 |
| 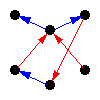 | 247 | 173 | 92 | 102 | 28 | 18 | 3/3 | 1/2 |
| 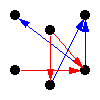 | 188 | 145 | 54 | 88 | 22 | 12 | 1/3 | 0/2 |
| 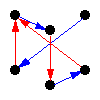 | 145 | 92 | 75 | 51 | 19 | 6 | 3/3 | 1/2 |
| 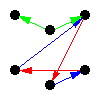 | 127 | 423 | 163 | 176 | 36 | 6 | 3/3 | 2/2 |
| 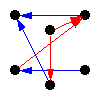 | 126 | 55 | 22 | 32 | 18 | 30 | 1/3 | 1/2 |
| 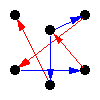 | 106 | 117 | 83 | 70 | 27 | 10 | 3/3 | 1/2 |
| 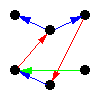 | 96 | 68 | 49 | 49 | 38 | 10 | 3/3 | 1/2 |
| 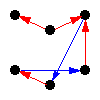 | 82 | 47 | 33 | 30 | 14 | 6 | 3/3 | 0/2^*^ |
| 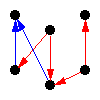 | 68 | 42 | 17 | 27 | 8 | 6 | 1/3 | 0/2 |
| 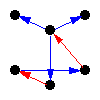 | 68 | 76 | 35 | 52 | 10 | 9 | 3/3 | 1/2 |
| 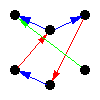 | 67 | 51 | 47 | 30 | 8 | 6 | 2/3 | 1/2 |
| 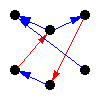 | 66 | 75 | 52 | 51 | 18 | 7 | 3/3 | 1/2 |
| 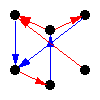 | 64 | 55 | 23 | 35 | 10 | 6 | 1/3 | 0/2 |
| 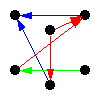 | 64 | 13 | 12 | 9 | 6 | 8 | 0/3 | 0/2 |
| 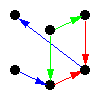 | 56 | 46 | 18 | 42 | 28 | 18 | 2/3 | 0/2 |
| 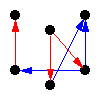 | 53 | 77 | 34 | 49 | 13 | 6 | 2/3 | 1/2 |
| 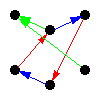 | 49 | 127 | 34 | 46 | 33 | 6 | 2/3 | 2/2 |
| 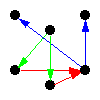 | 48 | 49 | 21 | 48 | 21 | 18 | 1/3 | 2/2 |
| 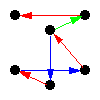 | 46 | 69 | 56 | 37 | 27 | 5 | 3/3 | 2/2 |
| 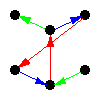 | 42 | 95 | 68 | 45 | 41 | 5 | 3/3 | 2/2 |
| 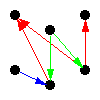 | 42 | 18 | 10 | 14 | 10 | 6 | 2/3 | 0/2 |
| 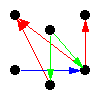 | 42 | 18 | 10 | 14 | 10 | 6 | 2/3 | 0/2 |
| 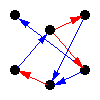 | 35 | 72 | 33 | 49 | 13 | 6 | 2/3 | 0/2 |
| 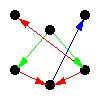 | 34 | 36 | 18 | 36 | 18 | 12 | 2/3 | 0/2 |
| 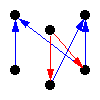 | 31 | 63 | 31 | 42 | 11 | 6 | 2/3 | 0/2 |
| 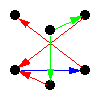 | 29 | 17 | 7 | 15 | 10 | 6 | 2/3 | 0/2 |
| 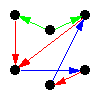 | 10 | 12 | 6 | 12 | 8 | 6 | 1/3 | 1/2 |
| 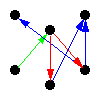 | 9 | 23 | 9 | 18 | 9 | 6 | 2/3 | 0/2 |
| 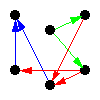 | 6 | 12 | 6 | 12 | 6 | 6 | 0/3 | 0/2 |

^a^Stimuli response pathways include mTOR signaling pathway, EGFR signaling pathway and TLR signaling pathway.

^b^Non-stimuli response pathways include influenza replication cycle and yeast cell cycle.

^*^Blue background color means that the motif did not appear in the category of maps.
