# Supplementary material for: Network analyses based on comprehensive molecular interaction maps reveal robust control structures in yeast stress response pathways
Source: NPJ Syst Biol Appl. 2016 Jan 7;2:15018–. doi: 10.1038/npjsba.2015.18 (PMC5516916; doi:10.1038/npjsba.2015.18)
Supplement: Supplementary Information S11 [file npjsba201518-s16.doc]

**Supplementary Information S11.** Instances of ‘reversible complex formation’ motif and ‘redundant reactions catalyzed by same molecule’ motif.

| **Reversible complex formation motif** | |
| --- | --- |
| Component molecules | **Maps in which the instance is included** |
| V1 subunit, V0(Stv1) subunit, V-ATPase(Stv1) | Ion |
| V1 subunit, V0(Vph1) subunit, V-ATPase(Vph1) | Ion |
| Bmh2, Msn2, Bmh2-Msn2 complex | Ion, Nutrient, Osmotic, Oxidative, Heat |
| Bmh2, Msn4, Bmh2-Msn4 complex | Ion, Nutrient, Osmotic, Oxidative, Heat |
| Bcy1, Tpk1, Bcy1-Tpk1 complex | Ion, Nutrient, Osmotic, Oxidative, Heat, Pheromone |
| Bcy1, Tpk2, Bcy1-Tpk2 complex | Ion, Nutrient, Osmotic, Oxidative, Heat, Pheromone |
| Bcy1, Tpk3, Bcy1-Tpk2 complex | Ion, Nutrient, Osmotic, Oxidative, Heat, Pheromone |
| Bmh1, Yak1, Bmh1-Yak1 complex | Nutrient, Oxidative |
| Bmh2, Yak1, Bmh2-Yak1 complex | Nutrient, Oxidative |
| Bmh1, Mks1,Bmh1-Mks1 complex | Nutrient |
| Bmh2, Mks1,Bmh2-Mks1 complex | Nutrient |
| H^+^, Mg^2+^, MgHPO_4_ | Ion |
| H^+^, PO4^3-^, MgHPO_4_ | Ion |
| Mg^2+^, PO4^3-^, MgHPO_4_ | Ion |
| H^+^, Mn^2+^, MnHPO_4_ | Ion |
| H^+^, PO4^3-^, MnHPO_4_ | Ion |
| Mn^2+^, PO4^3-^, MnHPO_4_ | Ion |
| glyceraldehyde 3-phosphate, dihydroxyacetone phosphate, fructose 1,6-biphosphate | Osmotic |
| Sit4, Tap42-TORC1 complex, Sit4-Tap42-TORC1 complex | Nutrient, Osmotic, Oxidative |
| Pph21, Tap42-TORC1 complex, Pph21-Tap42-TORC1 complex | Nutrient, Osmotic, Oxidative |
| Pph22, Tap42-TORC1 complex, Pph22-Tap42-TORC1 complex | Nutrient, Osmotic, Oxidative |
| Tap42, TORC1, Tap42-TORC1 complex | Nutrient, Osmotic, Oxidative |
| Rgt1, Med8, Med8-Rgt1 complex | Nutrient |
| ATP, fructose 6-phosphate, fructose 1,6-biphosphate | Nutrient |
| H^+^, NH_3_, NH_4_^+^ | Nutrient |

| **Redundant reactions catalyzed by same molecule motif** | |
| --- | --- |
| **Component molecules** | **Maps in which the instance is included** |
| cAMP, Bcy1 | Ion, Nutrient, Osmotic, Oxidative, Heat, Pheromone |
| calcineurin(CN), Crz1 | Ion, Heat |
| Amiodarone, Ca^2+^ | Ion |
| Vnx1, H^+^ | Ion |
| Nhx1, H^+^ | Ion |
| Mn^2+^, Smf2 | Ion |
