# Supplementary material for: Network analyses based on comprehensive molecular interaction maps reveal robust control structures in yeast stress response pathways
Source: NPJ Syst Biol Appl. 2016 Jan 7;2:15018–. doi: 10.1038/npjsba.2015.18 (PMC5516916; doi:10.1038/npjsba.2015.18)
Supplement: Supplementary File Legends [file npjsba201518-s17.doc]

**Legends for Supplementary information**

**Supplementary Information S1.** List of references used for the curation of the yeast stress response maps in this paper.

**Supplementary Information S2.** The SBML map files including IonHomeostasis.xml, NutrientAdapation.xml, OsmoticStressResponse.xml, OxidativeStressResponse.xml, HeatShockResponse.xml and PheromoneResponse.xml can be browsed using CellDesigner (downloadable at http://www.celldesigner.org/). For usage of the software, see the documentation provided at the CellDesigner website (http://www.celldesigner.org/documents.html).

**Supplementary Information S3.** PDF map files of each yeast stress response pathway.

**Supplementary Information S4.** A poster version PDF file of yeast stress response map.

**Supplementary Information S5.** Biological features of 6 stress response maps.

**Supplementary Information S6.** A list of the entities (proteins, genes, RNAs, and simple molecules) in all 6 yeast stress response maps. The appearance in each map and the number of occurrence throughout the 6 maps were summarized.

**Supplementary Information S7.** The visualization of bow-tie cores and driver/critical nodes of controllability in the maps. Molecules with high bow-tie scores are boxed in blue with the scores. Driver and critical nodes identified by the controllability analysis are indicated in green and red respectively.

**Supplementary Information S8.** The result summaries of the bow-tie analysis and the controllability analysis for all 6 yeast stress response pathways. Bow-tie scores using shortest paths (bowtie_short) and simple paths (bowtie_simple30) are represented in different column. Betweenness centrality of each molecular species are also shown in betweenness column.

**Supplementary Information S9.** The summary of the bow-tie analysis and the controllability analysis combined with experimental results. In the bow-tie column, core-associated genes with bow-tie score of >0.20 at least one map of the yeast stress response are represented as ‘core’. In the Controllablity column, genes whose correspondent proteins are included in at least one of critical node (protein or complex) were indicated as ‘Critical’ and others as ‘Non-critical’. Viability column represents whether single null mutant of a gene is viable or inviable. The number of synthetic lethal, negative genetic and positive genetic interactions are represented in Synthetic Lethality, Negative Genetic and Positive Genetic columns respectively. These viability and genetic interaction data were obtained from SGD database. The copy number limits measured with genetic tug-of-war (gTOW) method are shown in gTOW6000 column. We also performed controllability analysis using PPI network constructed from SGD physical interaction data. In the PPI controllability column, we can see whether correspondent protein of a gene is a critical node or not. Whether the criticality determined by PPI controllability analysis is consistent with the criticality based on the molecular interaction maps is summarized in consistency column. 62.3% of criticalities were consistent between the maps and the PPI.

**Supplementary Information S10.** The list of motifs common in the 6 yeast stress response maps.

**Supplementary Information S11.** The instances of ‘reversible complex formation’ motif and ‘redundant reactions catalyzed by same molecule’ motif among the yeast stress response maps.

Supplementary information is available at npj Sytstems Biology and Applications’s website.
