# Supplementary material for: Network analyses based on comprehensive molecular interaction maps reveal robust control structures in yeast stress response pathways
Source: NPJ Syst Biol Appl. 2016 Jan 7;2:15018–. doi: 10.1038/npjsba.2015.18 (PMC5516916; doi:10.1038/npjsba.2015.18)
Supplement: Supplementary Materials and Methods [file npjsba201518-s18.doc]

**Supplementary Materials and Methods**

**Building processes of comprehensive map**

The map was built following several distinct steps using CellDesigner™ 4.3.0 (http://celldesigner.org) software complying with standards SBML and SBGN . A top-down approach was followed for network construction focusing first on reviews and then on articles containing experimental evidence. Extensive literature scoping was performed using a comprehensive search tool called BioGyan ([www.biogyan.com](http://www.biogyan.com/)).

First, each signal transduction pathway was drawn roughly by using information obtained from review publications. Next, the preliminary maps were further modified and refined using data contained in the primary literature cited in the review publications. Third, additional data gathered from the SGD database and from publications obtained using PubMed keyword searches were incorporated.

The level of evidence obtained from publications varies from putative indirect effect to concrete direct molecular interaction. We gave top priority to the direct molecular interaction level evidence to represent a reaction. Each reaction was annotated with references of relevant papers and sentences therein that provide direct support for the molecular interaction. In case only indirect or unspecified evidence is available for a reaction, “Unknown Transition” was used to represent the reaction. For unspecified catalysis or inhibition, dotted lines (“Unknown Catalysis” or “Unknown Inhibition”) were used. It should be noted that another dotted line, “Unknown Reduced Trigger”, was used to represent omitted reactions from mRNAs to phenotypes. In this paper, we focused mainly on signal transduction of the stress response pathways, so other common biological processes including cell cycle and metabolism induced as a consequent of the gene regulation were represented as phenotypes ignoring details.

Due to the limitation of the CellDesigner™ software, we could not draw all of the genes and mRNAs (>2000) regulated by various transcription factors. For bow-tie and controllability analysis, the regulatory relationships between transcription factors and stress-related genes were obtained from YEASTRACT database ([http://www.yeastract.com](http://www.yeastract.com/)) and were appended to the directed graphs.

**Curation and Annotation**

A systematic and consistent workflow for curation and annotation is imperative for the construction of a large scale molecular interaction maps. We employed a top-down approach focusing first on review papers and next on detailed original experimental references. With conflicting and often controversial interpretation of reaction mechanisms, ambiguity in functions of molecules and knowledge gap in linking reactions, the process of curation and annotation needs to be defined *a priori* for a map. We delineate the key annotation schema employed in the construction of the signaling map,

1. *Naming convention*: To avoid confusion in cases where a factor has been assigned more than one name in the literature, we referred to the “Standard Name” as found in the *Saccharomyces Genome Database* (<http://www.yeastgenome.org/>).
2. *Compartmentalization*: Cell organelles are represented as compartments with various shapes as detailed below. Nucleus and the cytoplasm are represented as straight lines across the map. Endosome, Golgi complex, mitochondria and vacuole are represented as ovals while endoplasmic reticulum and peroxisome are denoted as squares. Due to space constraint caused by many factors concentrating on the membrane, not all reactions are reflected on it. Instead, such molecules and interactions are placed close to the membrane.
3. *Species representation:* DNA, RNA, proteins, molecular complexes, ions, ion channels, receptors, truncated proteins, drugs and phenotypes are represented by the notations available in CellDesigner™. For all other molecule types, simple molecule notation is used. Certain molecular complexes in the map are formed by association of two or more complexes. In such cases, the reactant complexes are denoted by the ‘compact’ view feature provided by CellDesigner™. On changing the complex view to ‘normal’, individual species that form the complex under consideration can be viewed. We used “Complex” only when components stably exist in the complex form which has the specific biological function. “Complex” was not used to represent transient binding in modification process such as phosphorylation.
4. *Reaction representation*: Reactions with direct biochemical evidence are represented by solid reaction arrows, while those with genetic but no biochemical evidence or with unclear mechanism are represented by dotted reaction arrows. Protein translation reactions are represented either in the nucleus or cytoplasm depending on the site of action of translated proteins (<http://celldesigner.org/>).
5. *Reaction and Species Notes:* In case of conflicting data and supplementation of the reaction’s representation, the specific reference paper as well as comments from the reference are annotated to the reaction notes or species notes on the model file.

For community-based annotation and curation, the map is posted under http://www.yeast-maps.org/yeast-stress-response/. Using iPathways+ (<https://ipathwaysplus.unit.oist.jp/>) and Payao, a web-based platform for sharing and curation of pathways, researchers can browse the map and provide further updates and improvements . The protocol for the curation using Payao is provided in “Curation” section in the website (http://www.yeast-maps.org/yeast-stress-response/Curation.html).

**Controllability analysis**

The controllability analysis considers linear dynamics


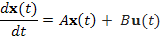


where the vector **x**(*t*) expresses the state of nodes at time *t*, the *N*
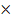
 *N* matrix *A* describes the interaction strength between the components and *B* is the *N*
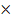
 *M* input matrix. The controllability analysis was applied to the maps as described previously . We define “control” as affecting concentration of a molecule. For example, if we consider a reaction in which A phosphorylates B, both concentration of A and non-phosphorylated B can affect the concentration of phosphorylated B. Thus, in this case, both A and non-phosphorylated B “control” phosphorylated B. The maps were converted into simple bipartite graphs by ignoring the type of reaction (e.g. In such reaction as ‘A is converted into B catalyzed or inhibited by C’, A and C are connected to B with arrows pointing towards B). The minimum set of driver nodes was determined that are necessary to gain full control of the network by using Hopcroft-Karp “maximum matching” algorithm . Then we identified critical node by examining whether the absence of the node requires an increase in the number of driver nodes. For the comparison with experimental phenotypes, all bipartite graphs of 6 maps were integrated together. Controllability analysis was applied to the integrated graphs and proteins included in at least one of critical node (protein or complex) were assigned as ‘critical’. Experimental phenotypes including viability and synthetic lethality of each genes were obtained from SGD database (<http://www.yeastgenome.org/>). Statistical differences between ratio of viable, synthetic lethal and non-lethal phenotypes of critical proteins and those of non-critical proteins were determined using Pearson’s chi-square test. Mann-Whitney two-tailed U test was employed to calculate statistical differences in number of negative genetic interaction between critical and non-critical proteins.

**Network motif analysis**

For network motif identification, the maps were converted into bipartite directed graphs treating both molecules and reactions as nodes. Edges were labeled with 3 colors: blue arrow means ‘reactant’ edge from reactant molecule node to reaction node, red arrow means ‘product’ edge from reaction node to product molecule node, and green arrow means ‘catalysis’ edge from enzymatic molecule node to reaction node. Six-node network motifs with labeled directed edge were extracted using FANMOD in full enumeration mode and with parameters of the random networks generating algorithm: number of networks = 100, exchanges per edge = 3, exchange attempts = 3, and regarding edge color. The randomization was performed by switching edges between nodes regarding the edge colors. Thus, degree distribution of the network is preserved. Motifs without labeled edge were extracted in the same manner not regarding edge colors.

**Bow-tie analysis**

A bow-tie score (b(*m*) ∊ [0, 1]) was defined to determine how ‘central’ a molecules *m* is in the signaling pathways as described previously with some modifications. Firstly, in a directed graph *G*, the source *S* and target *T* molecules (*S, T*
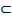
 *V*(*G*)) are assigned according to the property of the signaling network. In our case, we defined external stimuli as source *S* and mRNAs as target *T*. With given *S* and *T*, bow-tie score of a node *m* is given by the expression:


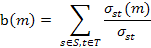


where
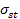
 is the total number of shortest paths from node *s* in the source *S* to node *t* in the target *T* and
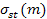
 is the number of those paths that pass through node *m*. As is the case for the betweenness centrality, the bow-tie score also scales with the number of pairs of nodes. Therefore the score was rescaled by dividing through by the number of pairs of source *s* and target *t* that have at least one connecting path, so that b(*m*) ∊ [0, 1].

Considering that biological signaling pathways have a lot of redundant paths with different length, calculating b(*m*) taking into account only shortest paths would miss molecules in alternative pathways. Therefore, we alternatively used simple paths, paths with no repeating vertices, within 30 lengths, where
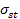
 is the total number of simple paths within 30 length from node *s* in the source *S* to node *t* in the target *T* and
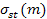
 is the number of those paths that pass through node *m*. With this modification, importance of alternative pathways was reflected.

For the weighted bow-tie analysis, transcriptome data was obtained from the NCBI Gene Expression Omnibus (GEO, www.ncbi.nlm.nih.gov/geo) through GEO Series accession numbers GSE4584, GSE54528, and GSE60613. Each connecting path was weighted using log fold change of target mRNA as a weight. Therefore, the weighted bow-tie score of a node *m* is given by the expression:


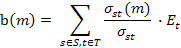


where *E_t_* is the log fold change of the target mRNA.

1. Hucka M, Finney A, Sauro HM, Bolouri H, Doyle JC, Kitano H, et al. The systems biology markup language (SBML): a medium for representation and exchange of biochemical network models. Bioinformatics. 2003 Mar 1;19(4):524-31.

2. Le Novere N, Hucka M, Mi H, Moodie S, Schreiber F, Sorokin A, et al. The systems biology graphical notation. Nature biotechnology. 2009;27(8):735-41.

3. Teixeira MC, Monteiro PT, Guerreiro JF, Gonçalves JP, Mira NP, dos Santos SC, et al. The YEASTRACT database: an upgraded information system for the analysis of gene and genomic transcription regulation in Saccharomyces cerevisiae. Nucleic acids research. 2013:gkt1015.

4. Matsuoka Y, Ghosh S, Kikuchi N, Kitano H. Payao: a community platform for SBML pathway model curation. Bioinformatics. 2010;26(10):1381-3.

5. Matsuoka Y, Matsumae H, Katoh M, Eisfeld AJ, Neumann G, Hase T, et al. A comprehensive map of the influenza A virus replication cycle. BMC systems biology. 2013;7(1):97.

6. Hopcroft JE, Karp RM. An n^5/2 algorithm for maximum matchings in bipartite graphs. SIAM Journal on computing. 1973;2(4):225-31.

7. Wernicke S, Rasche F. FANMOD: a tool for fast network motif detection. Bioinformatics. 2006;22(9):1152-3.

8. Supper J, Spangenberg L, Planatscher H, Dräger A, Schröder A, Zell A. BowTieBuilder: modeling signal transduction pathways. BMC systems biology. 2009;3(1):67.
